# Genome-wide identification and characterization of ABA receptor *PYL* gene family in rice

**DOI:** 10.1186/s12864-020-07083-y

**Published:** 2020-09-30

**Authors:** Shashank Kumar Yadav, Vinjamuri Venkata Santosh Kumar, Rakesh Kumar Verma, Pragya Yadav, Ankit Saroha, Dhammaprakash Pandhari Wankhede, Bhupendra Chaudhary, Viswanathan Chinnusamy

**Affiliations:** 1 Division of Plant Physiology, ICAR-Indian Agricultural Research Institute, Pusa Campus, New Delhi, 110012 India; 2 School of Biotechnology, Gautam Buddha University, Greater Noida, UP 201310 India; 3 ICAR-National Bureau of Plant Genetic Resources, Pusa Campus, New Delhi, 110012 India

**Keywords:** ABA receptors (ABARs), Abiotic stresses, Collinearity, miRNAs, Single amino acid polymorphism (SAP), Single nucleotide polymorphism (SNP), Stress responsive *cis*-elements, Synteny

## Abstract

**Background:**

Abscisic acid (ABA), a key phytohormone that controls plant growth and stress responses, is sensed by the pyrabactin resistance 1(PYR1)/PYR1-like (PYL)/regulatory components of the ABA receptor (RCAR) family of proteins. Comprehensive information on evolution and function of *PYL* gene family in rice (*Oryza sativa*) needs further investigation. This study made detailed analysis on evolutionary relationship between PYL family members, collinearity, synteny, gene structure, protein motifs, *cis*-regulatory elements (CREs), SNP variations, miRNAs targeting *PYLs* and expression profiles in different tissues and stress responses.

**Results:**

Based on sequence homology with *Arabidopsis* PYL proteins, we identified a total of 13 PYLs in rice (BOP clade) and maize (PACCMAD clade), while other members of BOP (wheat – each diploid genome, barley and *Brachypodium*) and PACCMAD (sorghum and foxtail millet) have 8-9 PYLs. The phylogenetic analysis divided PYLs into three subfamilies that are structurally and functionally conserved across species. Gene structure and motif analysis of *OsPYL*s revealed that members of each subfamily have similar gene and motif structure. Segmental duplication appears be the driving force for the expansion of *PYLs*, and the majority of the *PYLs* underwent evolution under purifying selection in rice. 32 unique potential miRNAs that might target *PYLs* were identified in rice. Thus, the predicted regulation of *PYLs* through miRNAs in rice is more elaborate as compared with *B. napus*. Further, the miRNAs identified to in this study were also regulated by stresses, which adds additional layer of regulation of *PYLs*. The frequency of SAPs identified was higher in *indica* cultivars and were predominantly located in START domain that participate in ABA binding. The promoters of most of the *OsPYL*s have *cis-*regulatory elements involved in imparting abiotic stress responsive expression. In silico and q-RT-PCR expression analyses of *PYL* genes revealed multifaceted role of ABARs in shaping plant development as well as abiotic stress responses.

**Conclusion:**

The predicted miRNA mediated regulation of *OsPYLs* and stress regulated expression of all *OsPYLs*, at least, under one stress, lays foundation for further validation and fine tuning ABA receptors for stress tolerance without yield penalty in rice.

## Background

Abscisic acid (ABA) plays a pivotal role in plant growth and development including cell elongation and division, embryo maturation, desiccation tolerance of seeds, seed dormancy, germination, leaf senescence, induction of root growth and fruit ripening. In addition ABA regulates stomatal aperture [1–6] and is the primary hormone imparting cellular tolerance to various biotic and abiotic stresses in plants [7–10].

Over the past one decade mammoth advancements have been made in unravelling the mechanism of ABA signalling including the discovery of ABA receptors [11]. The PYLs are currently the largest plant hormone receptor family known [12]. ABA is perceived by soluble cytosolic pyrabactin resistance 1 (PYR1)/PYR1-like (PYL)/ Regulatory component of ABA receptor (RCAR) protein family in Arabidopsis [13–15]. Binding of ABA to PYL leads to conformational change in PYL enabling it to bind to clade A type 2C protein phosphatases (PP2Cs). Binding of PYL to PP2C releases class III sucrose non-fermenting 1-related protein kinase 2 s (SnRK2s) from inhibition by PP2Cs [4, 11–13, 15–22]. Activated SnRK2s phosphorylate downstream targets like Abscisic acid responsive element (ABREs)/Abscisic acid binding factor (ABF)/ABI5 clade of bZIP transcription factors and other regulatory proteins promoting ABA induced physiological responses [16–19]. Thus, trinity of PYLs, PP2Cs and SnRKs constitute the core ABA signalling modules which are highly conserved in land plants which need abiotic stress tolerance for survival [15–26].

Voluminous efforts have been made in characterization of PYL receptors from model plant *Arabidospsis* which encodes for 14 PYL members that are highly conserved in amino acid sequence as well as in functional domain structure [13, 14, 27]. AtPYR1, AtPYL1 and AtPYL2 are dimeric, while AtPYL4 to AtPYL10 are monomeric in apo-receptor state. AtPYL3 exists in both monomeric and dimeric state. PYL receptors negatively regulate PP2Cs in an ABA independent manner [20]. Based on sequence similarity, ABA receptors of *Arabidopsis* have been broadly classified into 3 subfamilies [13]. ABA receptors belonging to subfamily I and II are monomeric, while subfamily III are dimeric in nature. The overall PYL structure exhibits the helix-grip fold, a hallmark of START (star-related lipid transfer) domain/Bet v 1-fold proteins, which is characterised by the presence of a central β-sheet surrounded by N- and C-termini α-helices, with a long C terminal α-helix packing tightly against the β-sheet. The helix-grip fold creates a large cavity constituting the ABA binding pocket [20, 23].

Extensive studies with PYLs in *Arabidopsis* have shown that PYL family ABA receptors play a diverse role in plant development and combating abiotic stresses. *AtPYR1, AtPYL1, AtPYL2, AtPYL4, AtPYL5, AtPYL8* and *AtPYL9* have been shown to promote ABA induced seed germination, stomatal closure and root growth; *AtPYL6* and *AtPYL13* have been shown to inhibit seed germination [13, 28–31]. Apart from playing key role in growth and development, *AtPYL5* and *AtPYL9* were found to provide drought tolerance [15, 32].

Since the discovery of PYL family of ABA receptors, a lot of efforts have been made to unravel the function of PYL members in diverse plant species and agriculturally important crops, including *Arabidopsis* [15, 22, 28, 32–37], *Artemisia annua* [38], *Vitis vinifera* [39, 40], *Oryza sativa* [41–47], *Triticum aestivum* [48, 49], *Zea mays* [50, 51], *Solanum lycopersicum* [52], *Glycine max* [53], *Populus* [54], *Hevea brasiliensis* [55], strawberry [56], *Gossypium hirsutum* [57–59], *Brassica rapa* [60] and *Brachypodium distachyon* [61, 62]. As compared with dicotyledonous model plant *Arabidopsis*, signalling modules in the monocot rice are similar in type and number which proves the conserved nature of functional ABA signalling pathway across plant species [49, 63–65].

Abiotic stresses such as cold, drought and salinity have detrimental effect on the agricultural crops leading to yield losses worldwide [66]. Despite the advancements achieved towards the comprehension of the role of *PYL* gene family in Arabidopsis, functional diversity and redundancy of PYLs in development and stress responsive processes in agronomically important crop like rice is relatively less investigated. In rice *OsPYL2*, *OsPYL8*, *OsPYL9*, *OsPYL10* and *OsPYL11* receptors have been functionally characterized by overexpressing the *PYL* genes while CISPR/Cas9 knockout mutants of PYL genes in rice has been found to moderate abiotic stress tolerance and yield [41–47]. Most of these works were focused in japonica rice while very less information is available in *indica* rice. Most of the mentioned approaches involved characterization using either overexpressing or knocking down individual *OsPYL* gene. Moreover previous expression studies of *PYL* genes were confined to a particular stage and stress. A detailed genome wide analysis of ABARs in terms of evolution, promoter analysis, miRNA targets and functional characterization has not been reported in rice. Functional validation of ABA receptor is pivotal for plant genetic engineering towards improving important agricultural traits such as plant biomass, yield and tolerance to abiotic stresses.

In the present study, genome wide identification and characterization of *PYL* gene family in *Oryza sativa spp. indica* was carried out using comparative genomic tools and experimental verification. A total of 13 *OsPYL* genes were identified from rice genome. Further we discerned *PYL* gene family in other agriculturally imperative crops and phylogenetic relationship amongst them. Genomic organization, gene structure, motif composition, subcellular localization and miRNA targets and expression analysis were characterized using in silico approaches. Collinearity and syntenic relationship of *PYL* gene across different taxas was studied. Non synonymous SNPs were identified across popular rice accessions to study polymorphism amongst them. We also carried out a detailed investigation of *cis*-regulatory elements in promoter region of ABA receptors in relation to their role in stress responsiveness and development. A comprehensive differential gene expression profiling of *OsPYL* gene family in spatiotemporal manner was carried out under different stresses (drought, ABA, low temperature, salinity, high temperature) and tissues using in silico data and quantitative PCR analysis. Our results provide a foothold in understating functions to further illuminate *OsPYL* genes under different stresses and development and identification of targets for improving abiotic stress tolerance in rice.

## Results

### Genome-wide identification and phylogenetic analyses of the *OsPYL* gene family in rice

To identify all the *OsPYL* gene members in rice, Hidden Markov model and BLASTp (e-value <=1e-10) searches were carried out to search rice genome annotation project (RGAP) (http://rice.plantbiology.msu.edu/) using 14 *Arabidopsis* PYL amino acid sequence as queries [67, 68]. A total of 13 *OsPYL* genes were identified in the genome of rice. Nomenclature of the identified *OsPYL* genes was done in accordance with previous study of *OsPYLs* [45]. Among these, two (OsPYL7 and OsPYL12) are thought to be non-functional ABA receptors as they have large deletion in the N and C terminal of the gene, respectively [26]. The identified rice *OsPYL* genes encode protein with size ranging from 125 (OsPYL12) to 229 (OsPYL6) amino acid residues. The other characteristics of the *OsPYL* genes, including gene length, open reading frame (ORF) length, the isoelectric point (pI), molecular weight (MW), and exons, are presented in (Table 1, Additional files 1, 2).

**Table 1:** Basic information of *OsPYL* family genes and their proteins in *Oryza sativa spp. indica*

Gene	Locus ID	^**a**^Accession No.	Gene length (bp)	ORF length (bp)	No. of Exon	Predicted Protein		
						Size (aa)	MW (kDa)	pI
OsPYL1	LOC_Os10g42280	KJ634481	639	639	1	212	23,083.91	5.45
OsPYL2	LOC_Os06g36670	KJ634482	931	624	1	207	22,344.15	6.31
OsPYL3	LOC_Os01g13330	KM371729	1069	633	1	210	22,760.58	6.45
OsPYL4	LOC_Os01g61210	KJ634480	627	627	1	208	22,295.33	8.26
OsPYL5	LOC_Os05g39580	KJ634479	1476	654	1	217	22,721.74	8.29
OsPYL6	LOC_Os03g18600	KJ634478	1286	690	1	229	23,815.95	6.89
OsPYL7	LOC_Os06g33480	3 K genome	3010	441	2	146	16,691.17	9.26
OsPYL8	LOC_Os06g33640	KJ634477	3411	621	1	206	23,321.71	5.99
OsPYL9	LOC_Os06g33690	KM371729	2062	621	3	206	23,397.74	6.45
OsPYL10	LOC_Os02g15640	KF925265	4129	615	3	204	23,068.25	6.46
OsPYL11	LOC_Os05g12260	KJ634476	2468	630	3	209	22,229.04	5.69
OsPYL12	LOC_Os02g15620	3 K genome	2049	378	2	125	13,682.57	5.16
OsPYL13	LOC_Os06g33490	3 K genome	2517	477	3	158	17,721.26	5.37

^a^Sequences with Accession numbers are cloned and sequenced from drought tolerant rice cv. Nagina 22 in our lab and are available in the NCBI https://www.ncbi.nlm.nih.gov/nucleotide/; Sequences where 3K genome is given in place of accession number, are the sequences of respective PYLs of Nagina 22 downloaded from rice 3000 genome database https://snp-seek.irri.org/

**Fig. 1:**
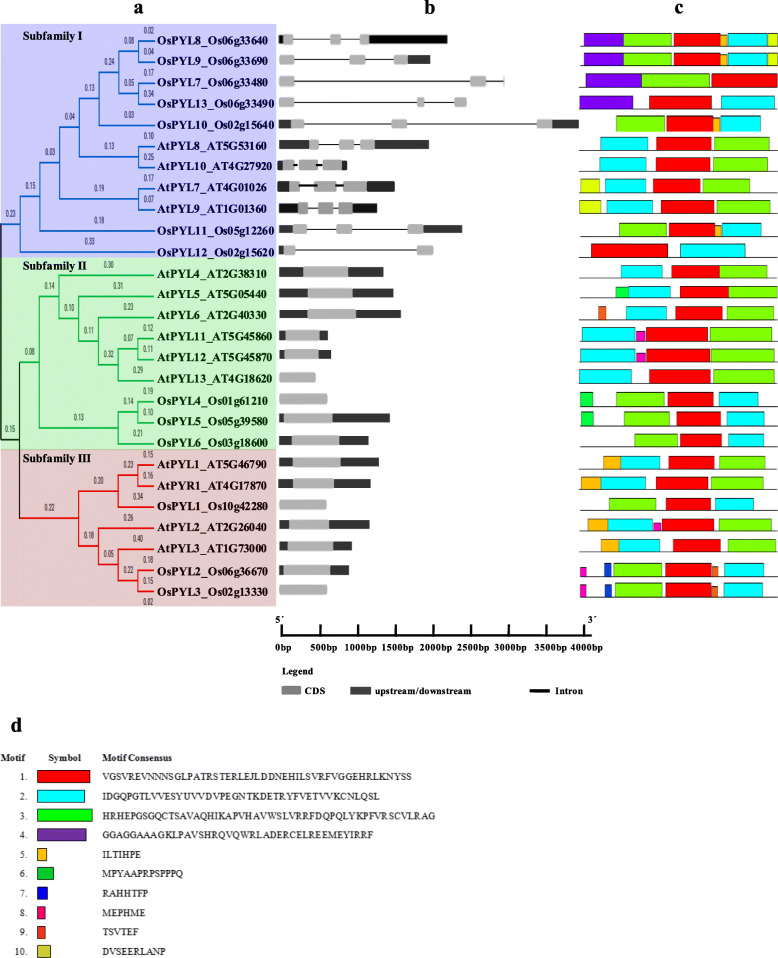
Phylogenetic relationship, gene architecture and conserved motifs of *PYL* genes. **a** Phylogenetic relationship of PYLs from in *Arabidopsis* and rice. Tree was constructed by the Maximum likelihood method. The blue, green, and red boxes depict the subfamily I, II and III, respectively. **b** Exon/intron architectures of PYLs. Grey colour boxes indicate exons and lines represent introns. The lengths of the exons and introns for each *PYL* gene can be calculated following the scale at the bottom. **c** Distributions of conserved motifs in PYL proteins. Motifs are indicated by 10 different colour boxes. **d** Legend depicting the protein sequence of the corresponding motifs

To understand the phylogenetic relationship of the OsPYL proteins, maximum likelihood method was used and tree was constructed using MEGA X [69, 70]. The OsPYL proteins could be grouped into three major subfamilies viz., sub-family I, II and III based on sequence similarity (Fig. 1a). Among the 13 OsPYL proteins, OsPYL7 to OsPYL13 (seven PYLs) belong to subfamily I; OsPYL4 to OsPYL6 belong to subfamily II (three PYLs), while OsPYL1 to OsPYL3 belong to subfamily III (three PYLs).

### Gene structure of *OsPYL* genes

Gene Structure Display Server (GSDS v2.0) [71] analysis of intron/exon structure of *AtPYL*s and *OsPYL*s (Fig. 1b) showed that the *AtPYLs* and *OsPYLs* can be distinctly grouped into intronless clade and a clade having introns. No introns were detected in the *PYL* genes of subfamilies II and III, whereas all of the members in subfamily I consists of two introns except *OsPYL7* and *OsPYL12*. Different *OsPYL* genes were grouped along with their homologous *AtPYL* genes in each subfamily depicting similar exon-intron structures, which affirms their close evolutionary relationships and the classification of subfamilies.

### Conserved motifs of *OsPYL*s

Among 13 OsPYLs, motif 1 harbouring the trademark Gate–Latch domain was conserved across all PYLs (Fig. 1c and Fig. 1d), while motif 2 and 3 were found to be conserved among all receptors except OsPYL7 and OsPYL12 (Table 1). OsPYLs have two (OsPYL12) to six (OsPYL2, 3, 8 and 9) motifs (Fig. 1c). Apart from sharing conserved motifs, each subfamily members have unique motifs. Putative functions of these motifs are given in Additional file 3 Table S1. These results indicate that *OsPYL* members clustered in the same subfamily show similar motif characteristics, suggesting functional similarities among members, while presence of unique motifs might carry out unique/specialized biological functions.

### Chromosomal distribution of *OsPYLs* across rice genome

**Fig. 2:**
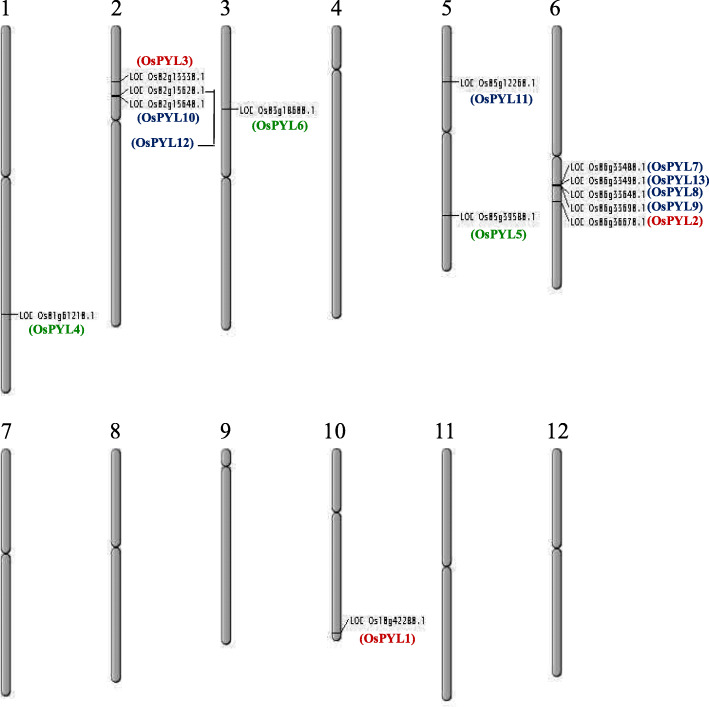
Chromosomal distribution of *PYL* genes on rice chromosomes. Chromosome numbers are showed at the top of each chromosome. The names of each *OsPYL* gene are shown on the right side of each chromosome. Subfamily I, II and III members are indicated in blue, green and red font, respectively. The bars on the chromosomes indicate the positions of the PYL genes. The figure was generated using Map Tool from Oryzabase

*OsPYL* genes were found to be unevenly distributed across 12 rice chromosomes (Fig. 2). Rice chromosome 6 harbours 5 *PYLs* (*OsPYL2*, *OsPYL7*, *OsPYL8*, *OsPYL9* and *OsPYL13*), chromosome 2 harbours 3 *PYLs*, (*OsPYL3*, *OsPYL10* and *OsPYL12*), and chromosome 5 harbours *2 PYLs* (*OsPYL5* and *OsPYL12*). Six of the 12 chromosomes do not harbour *PYL* genes.

### Identification of PYL members in other species

To study the evolutionary relationships between rice OsPYLs and PYLs of other grass family members, amino acid sequence OsPYLs were used as queries to search genome of wheat, maize, brachypodium, sorghum, foxtail millet and barley (Additional file 4). We identified 26 *TaPYLs* as previously reported [49]. Nine *TaPYL*s were identified in each diploid genome except *TaPYL2* which was absent in B genome. In maize, *Brachpodium*, foxtail millet, barley and sorghum 13, 10, 9, 9 and 8 PYLs, respectively, were identified. In OsPYL7, 8, 9 and 13 typical latch residues (HRL) are replaced by HML (Additional file 4).

**Fig. 3:**
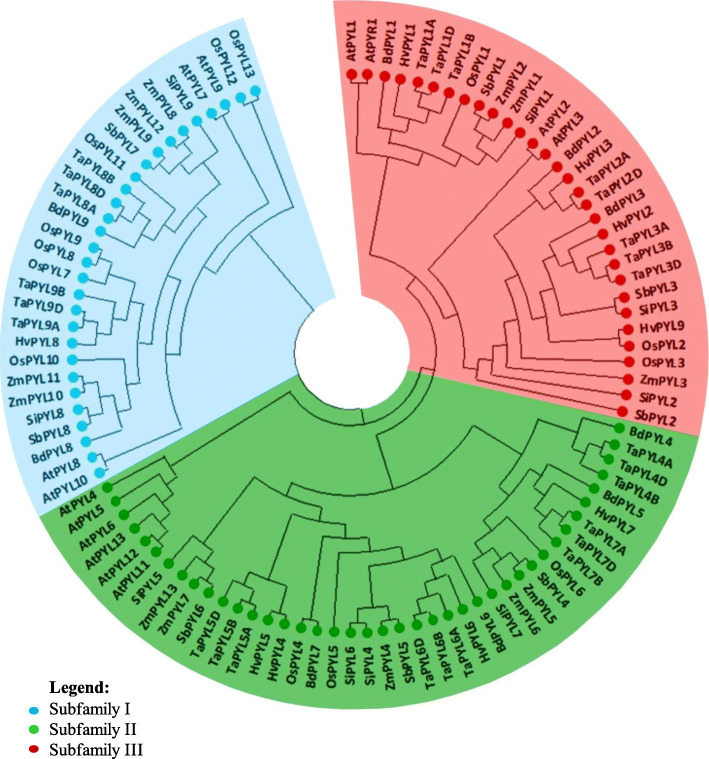
Phylogenetic analysis of PYL proteins from Arabidopsis (14 AtPYL), Brachpodium (9 BdPYL), Rice (13 OsPYL), Wheat (26 TaPYL), Maize (13 ZmPYL), Sorghum (8 SbPYL), Barley (9 HvPYL) and Foxtail millet (9 SiPYL) were used using the maximum likelihood method. The PYL proteins are classified into 3 subfamilies: I, II and III depicted by blue, green and red colour respectively

Based on the phylogenetic analysis, PYLs could be broadly classified to 3 subfamilies. There are 29, 41 and 31 members in Subfamily I, II and III, respectively in the eight species analysed. Therefore, PYL subfamily I and II have the lowest and highest PYL members, respectively, in grass family (Fig. 3).

### Genome wide synteny analysis of *PYL* gene family

**Fig. 4:**
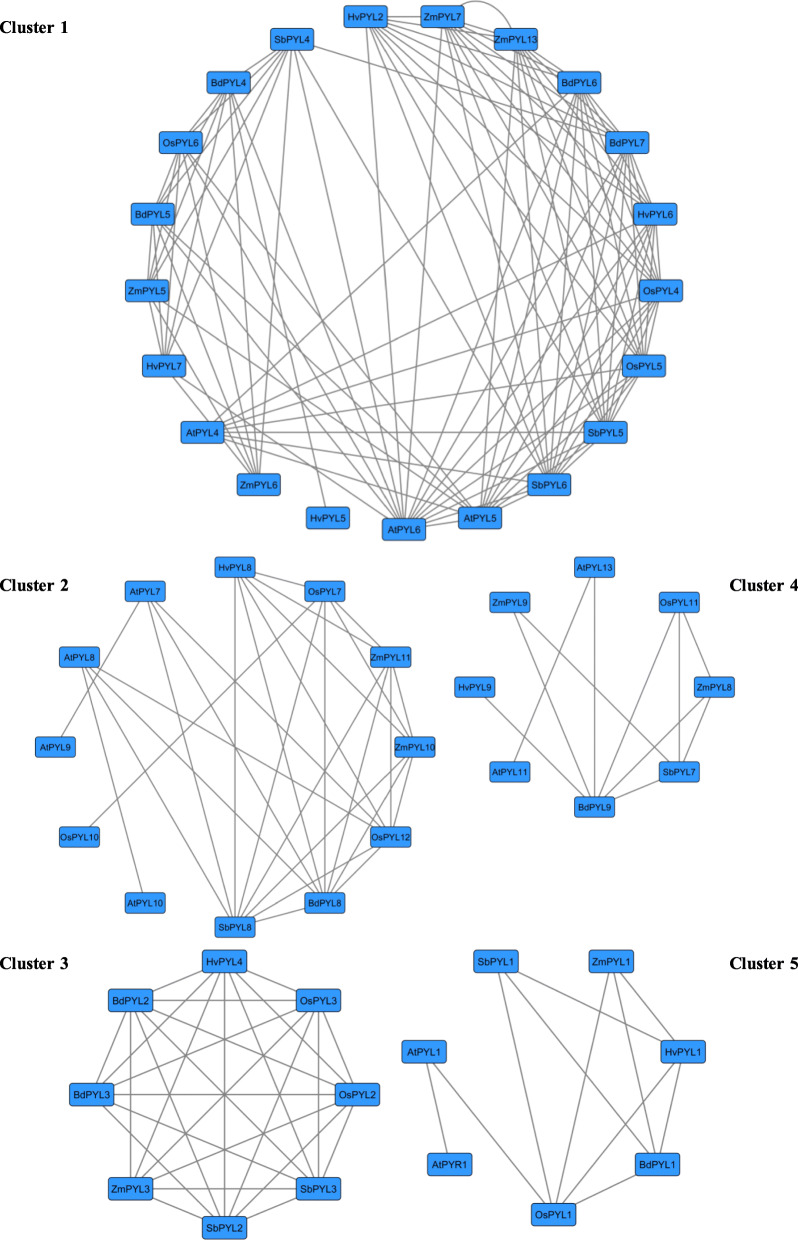
Synteny network of *PYL* genes across six different plant species Nodes represent syntenic genes and edges (lines) represent a syntenic connection between two nodes. 181 homologous gene pairs exist among PYL from *A.thaliana*, *B. distachyon*, *O. sativa*, *Z. mays*, *S. bicolor* and *H. vulgare* at genome wide scale. Each cluster represents genes with high sequence similarity

**Fig. 5:**
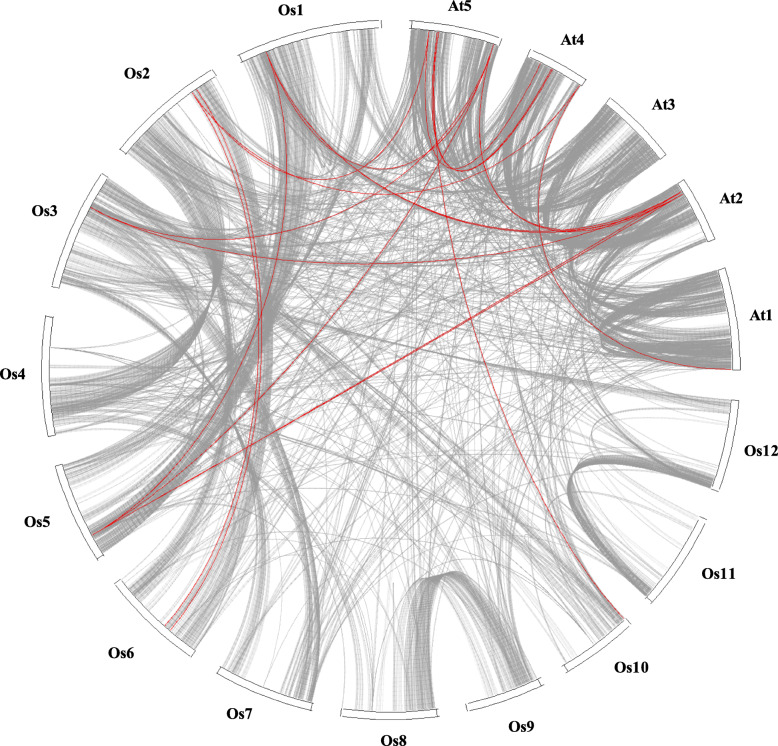
Circle plot showing collinearity in the *PYL* gene family. Collinearity in Arabidopsis and rice is highlighted with red curved lines over the gray background (genomic collinearity). Chromosomes are labelled in the format ‘species abbreviation’ + ‘chromosome ID’. At, *Arabidopsis thaliana*; Os, *Oryza sativa*

Gene duplication events including tandem and segmental duplications play an important role in broadening gene family during the evolutionary process [72]. Therefore, to gain insight into the genetic origins and evolution of the *PYL* gene family across six species, genome wide collinearity analysis was performed. Collinearity network grouped *PYL* genes across different taxas into five clusters where nodes represent individual *PYL* gene, while edges (lines between points) represent syntenic relationship amongst them (Fig. 4). Each cluster depicts high sequence similarity which might be a result of tandem gene duplication in the course of evolution. Interestingly, all *PYL* genes in cluster 1 belonged to subfamily II based on phylogenetic classification (Fig. 3). Notably cluster 3 comprising of PYL2 and PYL3 of different taxas was found to be least conserved as all the *PYL* genes form a closed interconnected network. On the other hand, *PYL* genes in cluster 1 were found to be most conserved. Further, we analysed whole genome duplication (WGD) events for *PYL* gene family between Arabidopsis and rice genomes by drawing whole genome Synteny blocks using CIRCOS (Fig. 5). Out of total 27 genes queried (14 Arabidopsis *PYLs* and 13 rice *PYLs*), only 20 *PYL* gene pairs formed collinearity blocks (11 Arabidopsis *PYLs* and 9 rice *PYLs*). Arabidopsis *PYL* genes were highly duplicated on chromosome two and four, while collinear gene pair between Arabidopsis and rice *PYL* genes was highest between Arabidopsis chromosome 2, 4, 5 and rice chromosome 1, 2 and 5. Some of the *PYL* genes exhibited multiple collinearity. We also found that collinearity of *PYL4*, *PYL5* and *PYL6* genes belonging to subfamily II was highest between the genome of two species. Moreover *AtPYL2*, and *AtPYL3* of subfamily III, *AtPYL12* of subfamily II*,* and *OsPYL8*, *9*, *11* and *13* of subfamily I do not form any collinearity blocks (Additional file 5 Table S2). These results further fortify our findings that *PYL* genes belonging to subfamily II and III have been evolutionarily conserved, while genes of subfamily I are least conserved. Collinearity blocks at genome scale of Arabidopsis and rice was also constructed which showed gene duplication was high within the species (Additional file 6 Fig. S1). Further to infer extent of selection pressure in the divergence of *PYL* genes, the non synonymous (Ka) and synonymous (Ks) values were evaluated for the orthologous gene pairs (Table 2). A total of 65 ortholog pairs were formed for which the average Ka/Ks value was 0.070, suggesting that PYL family across the species were under purifying or stabilizing selection during evolution (Additional file 7).

**Table 2:** The Ka/Ks values of the homologous PYL gene family in *Oryza sativa* among genome of *A.thaliana*, *B. distachyon*, *S. bicolor*, *Z. mays* and *H. vulgare*

PYL (***Oryza sativa***)	PYL Ortholog	S	N	Ka	Ks	Ka/Ks
OsPYL1	AtPYL1	108.60	455.40	66.4125	0.2662	0.0040
OsPYL1	BdPYL1	64.60	547.40	1.1968	0.0989	0.0826
OsPYL1	HvPYL1	63.30	539.70	0.8162	0.0679	0.0832
OsPYL1	SbPYL1	65.60	543.40	0.7075	0.0779	0.1101
OsPYL1	ZmPYL1	57.10	536.90	0.6550	0.0766	0.1169
OsPYL2	BdPYL2	90.70	521.30	84.1710	0.1534	0.0018
OsPYL2	BdPYL3	83.20	501.80	1.7773	0.0621	0.0349
OsPYL2	HvPYL2	79.30	502.70	0.4656	0.0539	0.1157
OsPYL2	OsPYL3	86.20	534.80	1.1002	0.1543	0.1402
OsPYL2	SbPYL3	69.40	524.60	1.1691	0.0812	0.0694
OsPYL2	SbPYL2	77.20	507.80	1.0677	0.1279	0.1198
OsPYL2	ZmPYL3	64.80	499.20	0.7683	0.1144	0.1489
OsPYL3	BdPYL2	85.30	535.70	0.9195	0.0863	0.0938
OsPYL3	BdPYL3	81.70	506.30	1.8213	0.1321	0.0725
OsPYL3	HvPYL2	70.90	505.10	1.0597	0.0933	0.0880
OsPYL3	SbPYL3	68.80	525.20	1.3384	0.1073	0.0802
OsPYL3	SbPYL2	67.50	535.50	0.6936	0.0961	0.1385
OsPYL3	ZmPYL3	57.10	503.90	0.5076	0.0532	0.1048
OsPYL4	AtPYL4	137.50	462.50	55.6376	0.3280	0.0059
OsPYL4	AtPYL5	109.20	442.80	64.5965	0.2737	0.0042
OsPYL4	AtPYL6	115.00	449.00	62.4790	0.3296	0.0053
OsPYL4	BdPYL6	85.30	502.70	1.7956	0.1362	0.0759
OsPYL4	BdPYL7	86.00	514.00	1.5294	0.1325	0.0866
OsPYL4	HvPYL2	79.60	502.40	1.1115	0.0729	0.0656
OsPYL4	HvPYL6	82.60	511.40	2.5193	0.1279	0.0508
OsPYL4	OsPYL5	80.20	522.80	2.3641	0.1013	0.0428
OsPYL4	SbPYL6	90.90	530.10	1.6537	0.2132	0.1289
OsPYL4	SbPYL5	82.40	511.60	3.1779	0.1080	0.0340
OsPYL4	ZmPYL7	82.30	523.70	2.3808	0.1514	0.0636
OsPYL4	ZmPYL13	90.40	506.60	1.3615	0.1732	0.1272
OsPYL5	AtPYL4	121.70	436.30	58.5149	0.2987	0.0051
OsPYL5	AtPYL5	110.70	453.30	65.0593	0.2808	0.0043
OsPYL5	AtPYL6	120.10	473.90	62.9904	0.3365	0.0053
OsPYL5	BdPYL6	76.10	505.90	0.9116	0.0703	0.0772
OsPYL5	BdPYL7	88.50	520.50	1.2382	0.1170	0.0945
OsPYL5	HvPYL4	90.70	515.30	1.2614	0.0797	0.0632
OsPYL5	HvPYL6	77.80	525.20	0.6748	0.0590	0.0874
OsPYL5	SbPYL6	82.50	529.50	2.5775	0.1714	0.0665
OsPYL5	SbPYL5	79.80	547.20	0.6968	0.0664	0.0953
OsPYL5	ZmPYL7	86.80	525.20	9.7639	0.1419	0.0145
OsPYL5	ZmPYL13	90.50	524.50	2.5277	0.1514	0.0599
OsPYL6	AtPYL5	109.10	463.90	66.9917	0.2994	0.0045
OsPYL6	AtPYL6	116.70	480.30	65.0515	0.3476	0.0053
OsPYL6	BdPYL4	100.60	520.40	2.6849	0.1572	0.0585
OsPYL6	BdPYL5	87.50	578.50	1.0429	0.0763	0.0732
OsPYL6	HvPYL7	88.60	577.40	0.7845	0.0630	0.0804
OsPYL6	SbPYL4	87.20	572.80	1.4762	0.0709	0.0480
OsPYL6	ZmPYL6	76.70	577.30	1.3192	0.0616	0.0467
OsPYL6	ZmPYL5	76.70	577.30	1.3192	0.0616	0.0467
OsPYL7	BdPYL8	104.80	297.20	2.4005	0.2484	0.1035
OsPYL7	HvPYL8	105.70	296.30	1.1841	0.2365	0.1997
OsPYL7	OsPYL10	99.10	311.90	1.9137	0.2234	0.1167
OsPYL7	SbPYL8	106.80	313.20	1.4210	0.2666	0.1876
OsPYL7	ZmPYL11	106.30	319.70	2.0012	0.2953	0.1475
OsPYL7	ZmPYL10	109.80	310.20	1.3024	0.2482	0.1906
OsPYL11	BdPYL9	130.50	475.50	0.8664	0.0327	0.0377
OsPYL11	SbPYL7	111.10	479.90	1.1054	0.0939	0.0850
OsPYL11	ZmPYL8	102.00	489.00	1.7432	0.0996	0.0572
OsPYL12	AtPYL7	104.30	258.70	44.4530	0.3180	0.0072
OsPYL12	AtPYL8	107.10	252.90	18.1991	0.3106	0.0171
OsPYL12	BdPYL8	90.60	272.40	7.1185	0.2313	0.0325
OsPYL12	HvPYL8	89.20	246.80	5.3474	0.3427	0.0641
OsPYL12	SbPYL8	75.80	287.20	9.5204	0.2396	0.0252
OsPYL12	ZmPYL11	66.30	206.70	16.3826	0.4896	0.0299
OsPYL12	ZmPYL10	80.20	282.80	8.0420	0.2375	0.0295

### Identification of miRNAs targeting *OsPYL* genes in rice

**Fig. 6:**
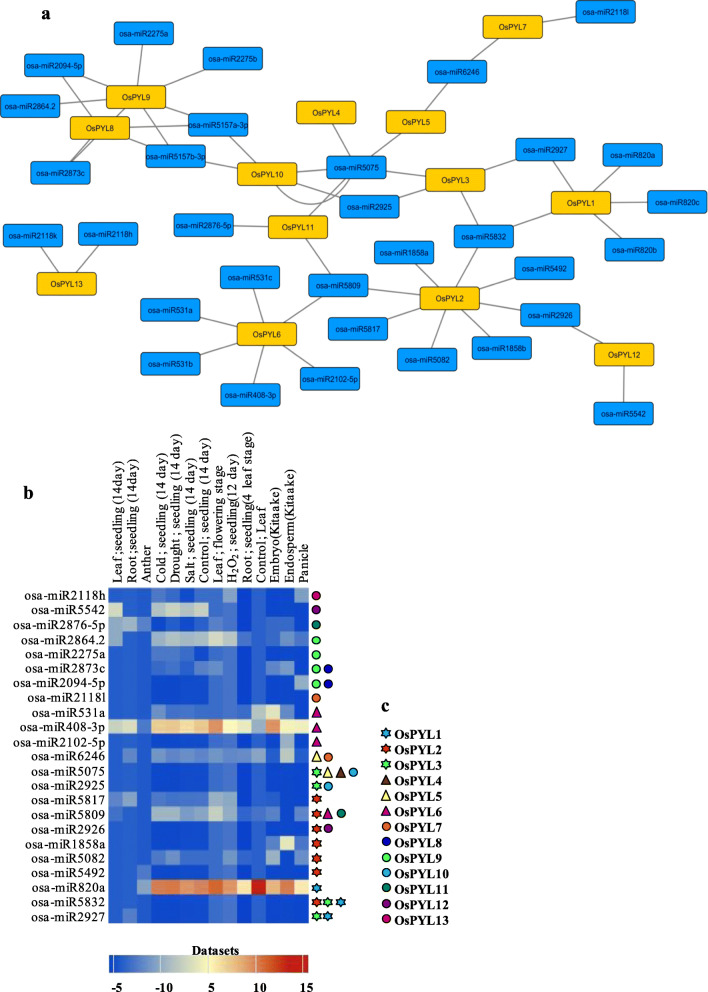
Identification potential miRNAs targeting PYL genes. **a** Schematic representation of targeted regulatory relations between miRNAs and their target PYLs using Cytoscape. Black line represents the interaction, and the blue and yellow box represents the miRNAs and its target *OsPYL* genes, respectively. **b** In silico expression analysis of putative PYL-targeting *miRNAs*. Log TPM (Transcript per million) values calculated after adding pseudocount of one to each miRNA count (Heat map generated by PmiRExAt http;//pmirexat.nabi.res.in/). **c** Different coloured circle, triangle and star represent PYL genes belonging to subfamily I, II and II respectively, targeted by the corresponding miRNAs

In order to predict miRNAs that may target *OsPYLs*, the cDNA sequences of *OsPYL* genes of rice were used as input in psRNATarget [73] against all the rice mature miRNAs available in miRbase [74] (Additional file 8). Total of 32 unique potential miRNAs targeting the *OsPYL* family members of rice were identified with mature miRNAs of 21–23 nucleotide long, Watson-Crick or G/U base pairing and stable minimal folding free energy (MFE). Some of the miRNAs were found to target specific subfamily. For example osa-miR5832 targets *OsPYL1*-*OsPYL3* of subfamily III, while osa-miR5075 targets *OsPYL4* and *OsPYL5* of subfamily-II and *OsPYL3* of subfamily I. Two members of osa-miR5157-3p family (osa-miR5157a-3p and osa-miR5157b-3p) were predicted to target *OsPYL8*, *OsPYL9* and *OsPYL10* of subfamily I. The miRNAs that potentially target *OsPYLs* ranged from minimum one (*OsPYL4*) to maximum of eight (*OsPYL2*) (Fig. 6a). The prominent inhibitory action by most of the miRNAs predicted to target *OsPYL* members across the three subfamilies was through cleavage (Additional file 8). Interestingly, most of the miRNAs were found to play a key role in stress responsiveness and development (Additional file 8). Microarray expression analysis of the identified miRNAs showed that majority of them are downregulated in different tissues and under stress conditions, while osa-miR820a and osa-miR408-3p targeting *OsPYL1* and *OsPYL6,* respectively, showed the highest expression level in all tissues and under different abiotic stresses (Fig. 6b). Thus, regulation of *OsPYL* genes through miRNA mediated sequence specific interaction might play a decisive role for plants to respond to growth and environmental stimuli.

### Identification of SNPs in *OsPYL* members

To get a deeper insight into allelic variation of *OsPYL* members across 12 rice varieties selected based on stress responses [75]. Rice SNP-Seek database [76] (https://snp-seek.irri.org/) was queried SNPs in *OsPYL* genes using Nipponbare reference genome. Among *13 PYL* genes in rice, non-synonymous SNPs were identified in 10 *OsPYL*s, but not in *OsPYL1*, *OsPYL5* and *OsPYL10* (Table 3)*.* This shows that *PYL1*, *PYL5* and *PYL10* genes are highly conserved across different rice genotypes. Interestingly, on protein structural basis, we found that majority of the Single Amino acid Polymorphisms (SAPs) were located across the helix grip fold domains, while some were located on CL loops surrounding ABA binding pocket [20, 23] (Additional file 9).

**Table 3:** Single Amino acid Polymorphisms (SAPs) in PYL proteins of selected rice genotypes

	Nipponbare	NERICA-1	NERICA-L-27	Vandana	GIZA-159	Azucena	Rasi	Swarna	Pokkali	IRAT-109	Nagina 22	IR-64	Pusa Basmati 1
**OsPYL2**	G_125_	G_125_	G_125_	G_125_	G_125_	G_125_	**A**_**125**_	G_125_	G_125_	G_125_	G_125_	G_125_	G_125_
	M_191_	M_191_	M_191_	M_191_	M_191_	M_191_	**K**_**191**_	M_191_	M_191_	M_191_	**K**_**191**_	M_191_	M_191_
**OsPYL3**	**–**	**–**	**–**	**–**	**–**	**–**	**S150_S154** **ins SPS**	**–**	**S150_S154** **ins SPS**	**–**	**S150_S154** **ins SPS**	**S150_S154** **ins SPS**	**–**
**OsPYL4**	F_49_	F_49_	**C**_**49**_	F_49_	F_49_	F_49_	**C**_**49**_	**C**_**49**_	**C**_**49**_	F_49_	**C**_**49**_	F_49_	F_49_
**OsPYL6**	A_86_	A_86_	A_86_	A_86_	A_86_	**P**_**86**_	A_86_	A_86_	A_86_	A_86_	A_86_	A_86_	A_86_
	K_215_	**N**_**215**_	**N**_**215**_	K_215_	K_215_	**N**_**215**_	K_215_	K_215_	K_215_	K_215_	**N**_**215**_	**N**_**215**_	**N**_**215**_
**OsPYL7**	V_132_	V_132_	V_132_	V_132_	V_132_	V_132_	**G**_**132**_	**G**_**132**_	**G**_**132**_	V_132_	**G**_**132**_	**G**_**132**_	V_132_
**OsPYL8**	R_21_	R_21_	R_21_	R_21_	R_21_	R_21_	R_21_	R_21_	R_21_	R_21_	R_21_	R_21_	**Q**_**21**_
	R_22_	R_22_	R_22_	R_22_	R_22_	R_22_	**Q**_**22**_	**Q**_**22**_	**Q**_**22**_	**Q**_**22**_	**Q**_**22**_	**Q**_**22**_	**Q**_**22**_
	V_23_	V_23_	V_23_	V_23_	V_23_	V_23_	**M**_**23**_	**M**_**23**_	**M**_**23**_	**M**_**23**_	V_23_	V_23_	V_23_
	C_25_	C_25_	C_25_	C_25_	C_25_	C_25_	**W**_**25**_	**W**_**25**_	**W**_**25**_	**W**_**25**_	**W**_**25**_	**W**_**25**_	**W**_**25**_
	K_30_	K_30_	K_30_	K_30_	K_30_	K_30_	**E**_**30**_	**E**_**30**_	**E**_**30**_	**E**_**30**_	**E**_**30**_	**E**_**30**_	**E**_**30**_
	V_99_	V_99_	V_99_	V_99_	V_99_	V_99_	**A**_**99**_	**A**_**99**_	**A**_**99**_	**A**_**99**_	**A**_**99**_	**A**_**99**_	**A**_**99**_
	D_173_	D_173_	D_173_	D_173_	D_173_	D_173_	**E**_**173**_	**E**_**173**_	**E**_**173**_	**E**_**173**_	**E**_**173**_	**E**_**173**_	**E**_**173**_
	V_187_	V_187_	V_187_	V_187_	V_187_	V_187_	**I**_**187**_	**I**_**187**_	**I**_**187**_	**I**_**187**_	**I**_**187**_	**I**_**187**_	**I**_**187**_
**OsPYL9**	G_3_	G_3_	G_3_	G_3_	G_3_	G_3_	G_3_	G_3_	G_3_	G_3_	G_3_	G_3_	**D**_**3**_
	A_11_	A_11_	A_11_	A_11_	A_11_	A_11_	A_11_	A_11_	A_11_	A_11_	A_11_	A_11_	**S**_**11**_
	N_133_	N_133_	N_133_	N_133_	N_133_	N_133_	**K**_**133**_	**K**_**133**_	**K**_**133**_	**K**_**133**_	**K**_**133**_	**K**_**133**_	N_133_
	V_150_	V_150_	V_150_	V_150_	V_150_	V_150_	V_150_	V_150_	V_150_	V_150_	**I**_**150**_	V_150_	V_150_
	P_172_	P_172_	P_172_	P_172_	P_172_	P_172_	**L**_**172**_	**L**_**172**_	**L**_**172**_	**L**_**172**_	**L**_**172**_	**L**_**172**_	P_172_
	V_187_	V_187_	V_187_	V_187_	V_187_	V_187_	V_187_	V_187_	V_187_	V_187_	V_187_	V_187_	**I**_**187**_
**OsPYL11**	V_144_	V_144_	V_144_	V_144_	V_144_	V_144_	V_144_	V_144_	**I**_**144**_	V_144_	**I**_**144**_	V_144_	V_144_
	D_152_	D_152_	D_152_	D_152_	D_152_	D_152_	D_152_	D_152_	**G**_**152**_	D_152_	D_152_	D_152_	D_152_
**OsPYL12**	P_22_	P_22_	**S**_**22**_	P_22_	P_22_	P_22_	P_22_	P_22_	P_22_	P_22_	P_22_	**S**_**22**_	P_22_
	V_79_	V_79_	I_79_	V_79_	V_79_	V_79_	V_79_	V_79_	V_79_	V_79_	V_79_	**I**_**79**_	V_79_
**OsPYL13**	N_62_	N_62_	N_62_	N_62_	N_62_	N_62_	**S**_**62**_	**S**_**62**_	**S**_**62**_	**S**_**62**_	**S**_**62**_	**S**_**62**_	**S**_**62**_
	D_99_	D_99_	D_99_	D_99_	D_99_	D_99_	**N**_**99**_	**N**_**99**_	**N**_**99**_	D_99_	D_99_	D_99_	D_99_

### Identification of *cis*-regulatory elements (CREs) in promoters of *OsPYLs*

**Fig. 7:**
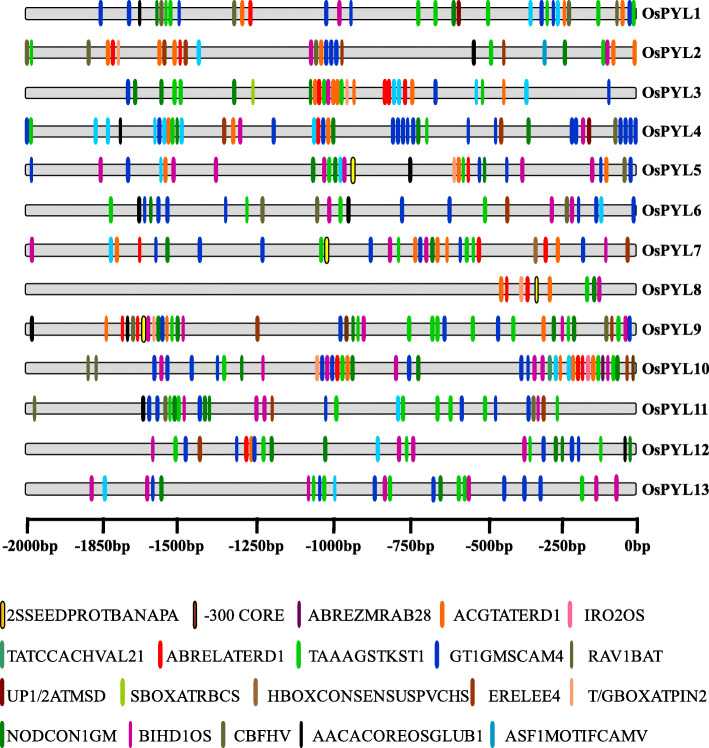
*cis*-regulatory Elements (CREs) in the promoter of *PYL* genes. Positional distribution of predicted CREs on *OsPYL* promoters are shown as vertical bars. Promoter sequences (− 2000 bp) of thirteen *OsPYL* genes were analyzed by using NewPLACE. Legend depicting the colour of individual *cis* elements

**Fig. 8:**
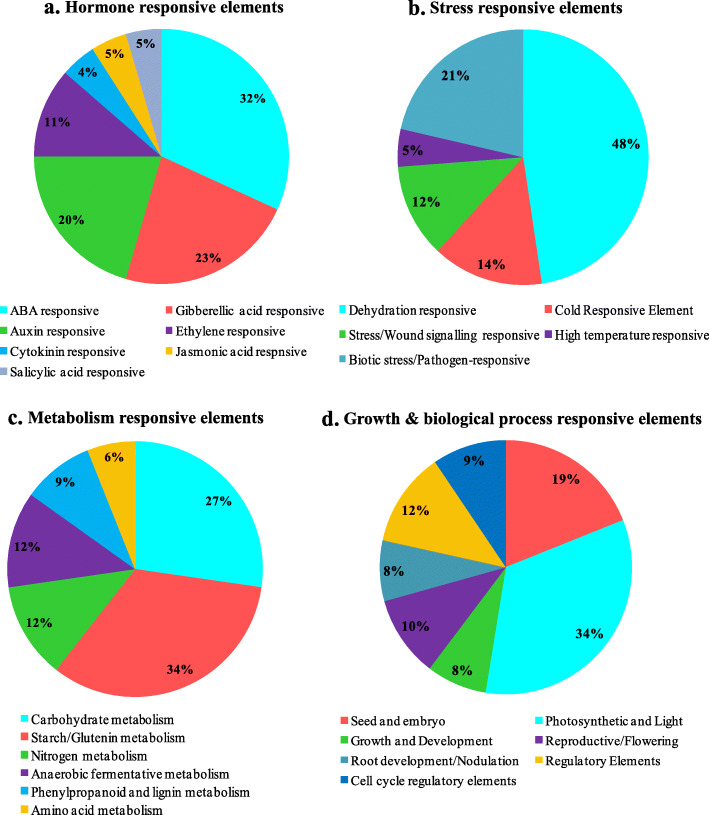
Percentage wise distribution of *cis*-regulator elements (CREs) in the promoters of *OsPYL* genes based upon the putative functions. **a** Hormone responsive CREs. **b** Stress responsive CREs. **c** Metabolic responsive CREs. **d** Growth and biological process responsive CREs

Rice, being a sensitive crop to various biotic and abiotic stresses, needs to adapt swiftly to frequently changing stresses [66]. The control of gene transcription via CREs in the promoter remains a pivotal mode of regulation of gene expression. To investigate the potential CREs in *OsPYL* gene family in rice, the promoter sequences of approximately 2.0-kb upstream from the translation start sites of individual PYL receptors, were taken from RGAP [68] and searched against New PLACE [77] (https://www.dna.affrc.go.jp/PLACE/?action=newplace). A total of 193 putative CREs were predicted across thirteen *OsPYL* genes with a range from 62 to 105 CREs (Additional file 10). The prevalence distribution of 20 key CREs are schematically depicted (Fig. 7). Some of these elements are conserved across *OsPYL* family, and might be critical in imparting stress and developmental regulation. We have identified certain CREs that can be functionally attributed to individual *OsPYL* members or subfamily. For an example *OsPYL*8 and *OsPYL*9 has endosperm and seed specific CREs like -300CORE [78], and 2SSEEDPROTBANAPA [79]. Previously these elements have been reported to be associated with endosperm specific activity of *OsPYL8* and *OsPYL9* [46]. Likewise ABREZMRAB28 is induced by ABA and is a binding site for CBFs [80]. It has been recently reported that overexpression of *OsPYL*10 imparts dehydration and freezing tolerance in transgenic rice [47]. We have identified *cis*-element TAAAGSTKST1 [81] which is a target site for transcription factor governing guard cell specific gene regulation in stomata. All the identified CREs have been grouped into four major categories based upon function and their response to stimuli (Fig. 8). In this study, hormone responsive elements like abscisic acid responsive elements (ABRE) form the major proportion of CREs. Interestingly gibberellic acid responsive elements (GARE) and auxin responsive elements (ARE) were also found in abundance in the promoters, suggesting potential hormonal cross talk at the expression level of ABA receptors (Fig. 8a). Amongst stress responsive elements, dehydration responsive elements (DRE) forms the major group followed by low temperature responsive elements (LTRE) (Fig. 8b). On the basis of metabolic functions, the proportion of amylose/starch responsive elements (34%) followed by carbohydrate responsive elements (27%) was notably highest, while elements involved in amino acid metabolism comprised least (6%) of the CREs identified (Fig. 8c). The proportion of elements that might be involved in photosynthetic machinery and light responsive elements (LRE) was found to be highest (34%) followed by seed and endosperm specific elements (19%) (Fig. 8d). All *OsPYL* promoters had more than one stress-response-related CREs. CREs associated with hormonal regulation, including ABRE, AuxRR-core, CGTCA motif, P-box, TCA-element, and TGA-element were identified in most of the *OsPYL* genes promoter. TC-rich repeats, which are involved in low temperature, drought inducibility and defence responsiveness, were also found in many *OsPYLs* (Additional file 11). This suggests that *OsPYL* genes are regulated by diverse development and stress responses.

### In silico expression analysis of *OsPYL* genes

**Fig. 9:**
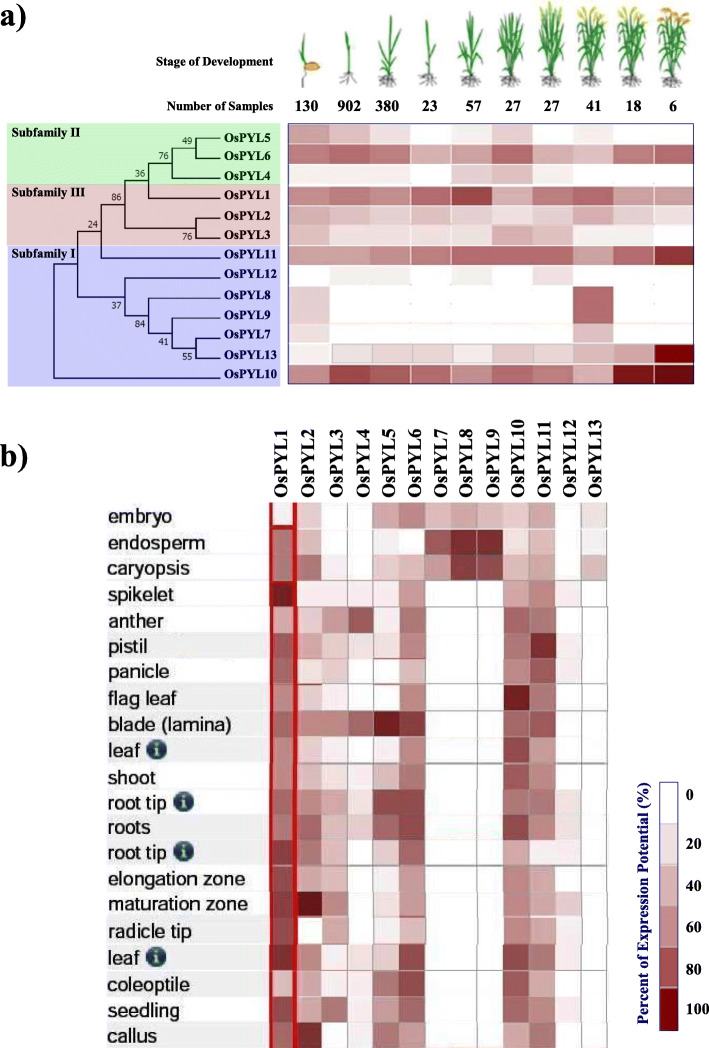
Expression potential of *PYL* genes in different tissues and developmental stages of rice. **a** Developmental stages. Stage development shown in the picture are Germination, seedling; tiller initiation; stem elongation, booting, heading, anthesis, milk stage of grain development, dough stage of grain development and mature grain stage (top panel). **b** Different tissues. Expression analysis was carried out with mRNAseq datasets using Genevestigator

**Fig. 10:**
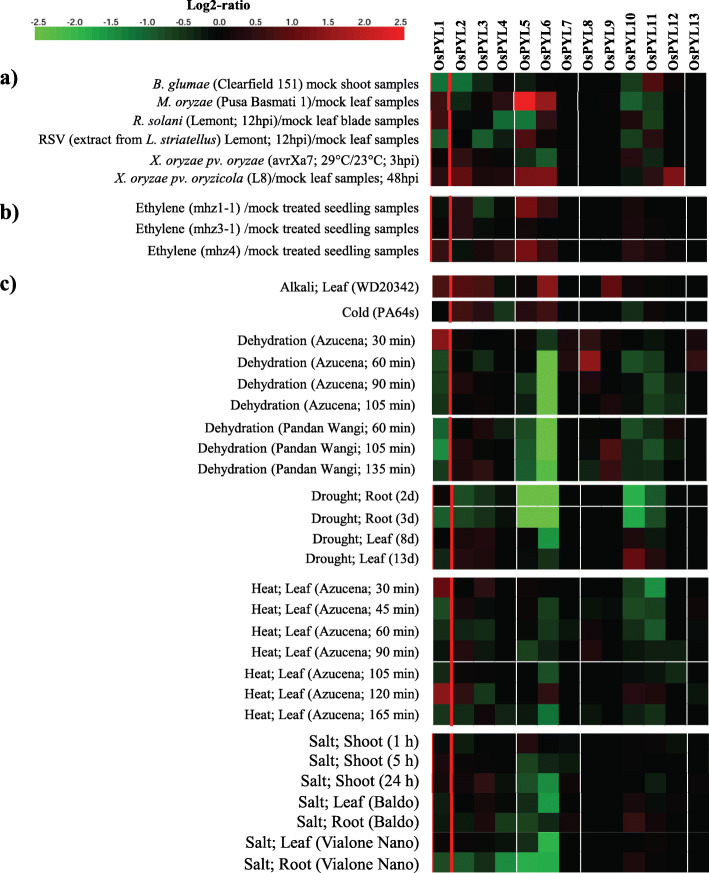
Abiotic stress regulated expression of *PYL* genes in rice. Expression analysis was carried out with mRNAseq datasets using Genevestigator. **a** Biotic stress. **b** Ethylene. **c** Abiotic stresses. The colour scale represents Log2 of average signal values

Spatiotemporal and stress responsive expression analysis of *OsPYL* genes was carried out using GENEVESTIGATOR database [82] (https://genevestigator.com/gv/). *OsPYL1*, *OsPYL2*, *OsPYL6*, *OsPYL10* and *OsPYL11* showed expression in most tissues across developmental stages (Fig. 9a and b). *OsPYL7*, *OsPYL8* and *OsPYL9* were found to be specifically expressed in endosperm and embryo suggesting their potential role in seed development in rice. In response to rice blast fungus (*Magnaporthe oryzae*) infection, *OsPYL5* and *OsPYL6* showed upregulation in leaf of indica cv. Pusa Basmati 1. Bacterial leaf streak pathogen (*Xanthomonas oryzae pv. oryzicola*) inoculation also induced these two PYLs and *OsPYL12* (Fig. 10a). Ethylene moderately upregulated only *OsPYL5* (Fig. 10b). Alkali treatment induced the expression of *OsPYL6* to > 1.5 folds in leaves Drought stress induced the expression of *PYL1*, *PYL8* and *PYL10* by > 1.5 fold. Interestingly, drought stress upregulated the expression of *PYL10* by > 1.5 fold leaf but it was downregulated in the roots (Fig. 10c). Heat stress upregulated *OsPYL1* and downregulated *OsPYL6* in leaf. Both drought and salt stresses downregulated *OsPYL5* and *OsPYL6* (Fig. 10c).

### Real time qRT-PCR expression profiling of *OsPYL* genes in different tissues

**Fig. 11:**
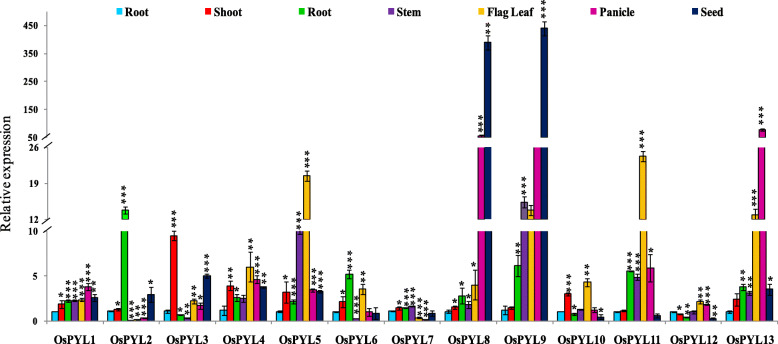
Real-time qRT-PCR expression analysis of tissue specific expression of *OsPYL* genes in rice. Tissue specific expression of *OsPYLs* was analyzed by qRT-PCR in seed, root and shoot at seedling stage, and root, stem, flag leaf and panicle at reproductive stage of indica rice cv. Nagina22. The expression level in the root of seedling was taken as calibrator (1) and the fold change was analyzed via the 2^-ΔΔCT^ method using the rice Ubiquitin gene as an internal control. Values represent the mean + SD of three biological replicates

Tissue and stress responsive expression of *PYL* genes were analysed to understand their role in development and stress tolerance. *OsPYL8* and *OsPYL9* showed with highest expression in seeds, while *OsPYL1*, *OsPYL11* and *OsPYL13* showed highest expression in panicle among the tissues (Fig. 11). Among the PYLs, *OsPYL2* showed highest expression in roots of the plants at reproductive stage as compared with that in other tissues. Interestingly, many *PYLs* (*OsPYL1*, *OsPYL4*, *OsPYL5*, *OsPYL8*, *OsPYL9*, *OsPYL11*, *OsPYL12* and *OsPYL13*) also showed high levels of expression in stem tissue at reproductive stage as compared with that in seedling roots (Fig. 11).

### Real time qRT-PCR expression profiling of *OsPYL* genes in responses to abiotic stresses

**Fig. 12:**
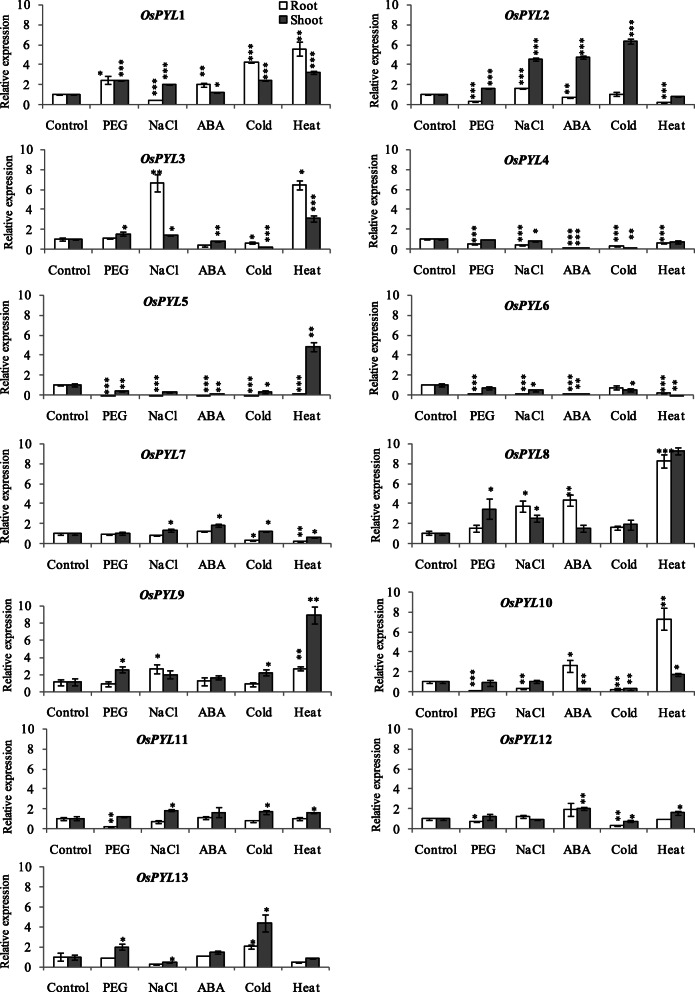
Real-time qRT-PCR expression analysis of *OsPYL* genes under abiotic stresses at seedling stage in rice. Total RNA isolated from 14 days old seedlings of rice cv. Nagina22 treated with osmotic stress (PEG), salt, cold, heat and ABA treatments for 6 h and control plants were used for qRT-PCR analysis. The expression level in control sample was taken as calibrator (1) and the fold change was analyzed via the 2^-ΔΔCT^ method using the rice ubiquitin gene *(UBQ)* was used as an internal control. Values represent the mean + SD of three biological replicates

All the subfamily III *OsPYLs* were significantly upregulated by all the abiotic stresses at least in one tissue at seedling stage in drought tolerant rice cv. Nagina 22 (Fig. 12). *OsPYL1* was significantly upregulated in both root and shoot at seedling stage by abiotic stresses except NaCl which downregulated its expression in root (Fig. 12). Similarly contrasting tissue specific regulation was observed for *OsPYL2* under osmotic (PEG) stress and ABA, where it was upregulated in shoot and downregulated in root at seedling stage. *OsPYL3* was upregulated only in shoot by PEG stress, while it was upregulated both in root and shoot by NaCl and heat stress, and downregulated in the both the tissue by cold stress (Fig. 12). The subfamily II *OsPYLs* (*PYL4*, *PYL5* and *PYL6*) were mostly downregulated both in root and shoot by all stresses and none were upregulated by stress at seedling stage (Fig. 12). Among subfamily I *OsPYLs* (*PYL7-PYL13*), *OsPYL8*, *OsPYL9* and *OsPYL13* were significantly upregulated by osmotic stress (PEG) in shoot. Salt stress significantly upregulated *OsPYL7*, *OsPYL8* and *OsPYL11* in shoot, and *OsPYL8* and *OsPYL9* in root. Cold stress significantly upregulated *OsPYL7*, *OsPYL9* and *OsPYL11* in shoot, and *OsPYL13* in both root and shoot at seedling stage. Heat stress significantly upregulated *OsPYL8*, *OsPYL9* and *OsPYL10* in both root and shoot, and *OsPYL11* and *OsPYL12* only in shoot at seedling stage (Fig. 12). ABA regulated the expression of all *OsPYLs* except *OsPYL9*, *OsPYL11* and *OsPYL13* at least in one tissue at seedling stage in rice ABA significantly upregulated *OsPYL2*, *OsPYL7* and *OsPYL12* in shoot, and *OsPYL1*, *OsPYL8* and *OsPYL10* in root at seedling stage (Fig. 12).

**Fig. 13:**
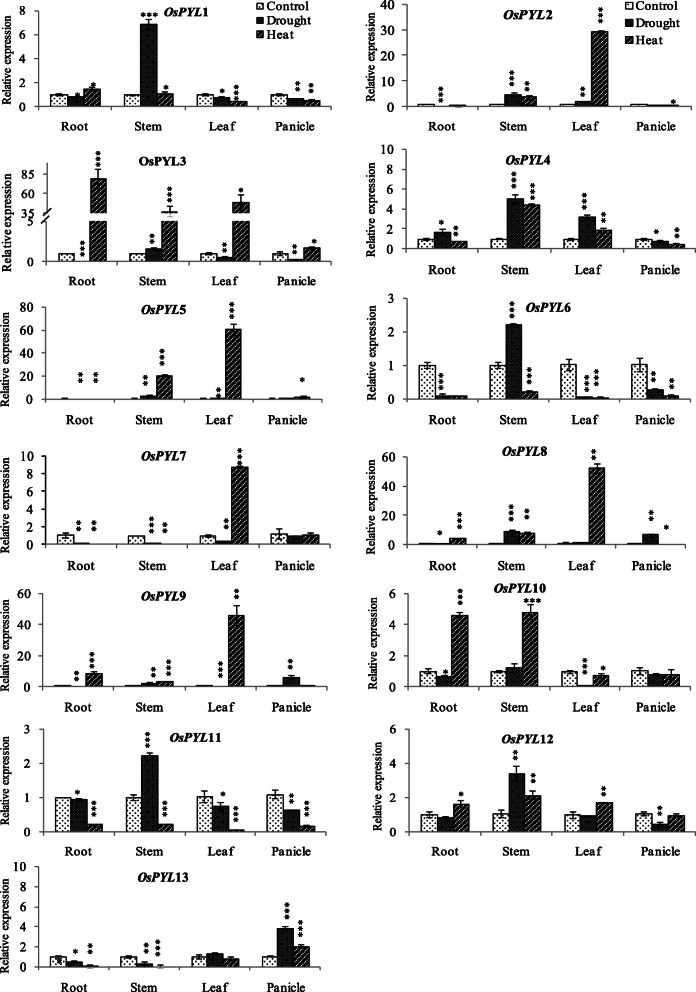
Real-time qRT-PCR expression analysis of *OsPYL* genes under drought and heat stress at flowering stage in rice. Total RNA isolated from rice cv. Nagina22 at anthesis stage was subjected to drought (− 80 kPa) and heat stress, and control plants were used for qRT-PCR analysis. The expression level in control sample was taken as calibrator (1) and the fold change was analyzed via the 2^-ΔΔCT^ method using the rice ubiquitin gene *(UBQ)* was used as an internal control. Values represent the mean + SD of three biological replicates

At reproductive stage, tissue specific regulation of *PYLs* was observed under drought stress. In root tissue, drought stress significantly upregulated the expression of *OsPYL4*, while it downregulated rest of the *OsPYLs* except *OsPYL12*. In contrast, in the stem tissue, drought stress significantly upregulated most *OsPYLs*, except *OsPYL7* and *OsPYL13* which were downregulated, and *OsPYL10* which was unaltered. Drought stress significantly upregulated *OsPYL2* and *OsPYL4* in flag leaf and *OsPYL8, OsPYL9* and *OsPYL13* in panicles (Fig. 13). Thus, at reproductive stage, drought stress either up- or down-regulated the expression all the *OsPYLs* in most of the four different tissues examined (Fig. 13).

Heat stress at reproductive stage differentially regulated the expression of all *OsPYLs* in vegetative tissues (root, stem, leaf) except that of *OsPYL1* in stem, while in panicle it regulated most *PYLs* except *OsPYL7, OsPYL10* and *OsPYL12*. Among the subfamily III members, *OsPYL3* was significantly upregulated under heat stress in all four tissues (Fig. 13). Among the subfamily II members, *OsPYL6* was downregulated in all four tissues, *OsPYL4* was upregulated in stem and leaf but downregulated in root and panicle, and *OsPYL5* was downregulated in roots and upregulated in rest of the three tissues under heat stress (Fig. 13). Among the subfamily I members, *OsPYL8, OsPYL9*, *OsPYL10* and *OsPYL12* were significantly upregulated in both root and stem, *OsPYL7*, *OsPYL8* and *OsPYL9* in flag leaf and *OsPYL13* in panicle under heat stress (Fig. 13).

## Discussion

### Gene structure and evolution

ABA is a key phytohormone that governs various plant development and stress response processes. ABA is perceived by PYL family of receptors, which are the largest plant hormone receptor family known [12]. Despite arduous efforts investigation on *PYL* gene family has largely been confined to characterization of *PYL* genes mainly in Arabidopsis, while only limited efforts have been made on genome wide characterization of *PYL* gene family for elucidating their role in multiple stresses and their evolutionary relationship in major crops. In the present study, a genome wide comprehensive analysis of *PYL* genes in rice and its potential role in development and abiotic stress responses was investigated using bioinformatic and experimental approaches. A total of 13 *OsPYLs* were identified from rice genome using Arabidopsis PYL protein sequence as queries as previously reported [44]. The sequences of all 13 *OsPYL* genes from upland indica rice variety Nagina22 were used in the present study. Based on phylogenetic relationship with Arabidopsis, *OsPYL* family can be broadly classified into three subfamilies: I, II and III (Fig. 1a). Subfamily II and III PYLs of rice and Arabidopsis are intronless, while all seven members of *OsPYLs* belonging to subfamily I have introns (Fig. 1b). The organizations of intron/exon and the number of exons in the surveyed rice ABA receptors were similar to those orthologs in maize, tomato, rubber, cotton, brachypodium and sorghum [54, 56, 59, 61, 63, 83]. These results imply that the exon/intron organizations of *OsPYL*s are closely related to the phylogenetic relationship of the genes (Fig. 1a). Introns play key role in post-transcriptional regulation gene expression by splicing-dependent and splicing-independent intron-mediated enhancement of mRNA accumulation [84]. All the PYL members of subfamily I has evolved introns to fine tune their regulation.

To further understand the diversification of *PYL* gene family at protein level, ten conserved motifs were acquired for each protein using MEME Suit (Fig. 1c). All the PYL proteins had motif 1 that has the conserved Gate and Latch domain of PYL receptor [23, 28]. Motif 2 and motif 3 were present in all PYL members except the non-functional OsPYL7 and OsPYL12. HHpred analysis to affirm if the motifs obtained from the MEME analysis are similar to any of the known protein motifs revealed that Motif1, 2 and 3 belongs to Polyketide cyclase/dehydrase and lipid transport superfamily protein. It was observed that these novel motifs did not show any significant similarity with the known motifs (Additional file 3). This indicates that all the identified OsPYLs have typical subfamily features and the proteins classified into the same subgroup share similar protein motifs.

ExPaSy analysis of physical properties of the OsPYLs from indica rice cultivar Nagina 22 showed that except OsPYL7, OsPYL12 and OsPYL13, all other receptors had similar protein length and physical properties (Table S1). Amino acid sequence of most ABA receptor were quite similar between japonica and indica baring few SNPs in *OsPYL*2, *OsPYL*3 and *OsPYL*9 (NCBI Accession no. KJ634482, KM371729, KM371729). Compared to japonica rice, *OsPYL3* in indica rice has an insertion of nine nucleotides, increasing the amino acid sequence by three residues.

Chromosomal distribution of *PYL* genes across 12 rice chromosomes showed uneven distribution of *PYLs* with as many as five PYLs located on six chromosomes. *OsPYL8* and *OsPYL9* having 95.5% sequence similarity at nucleotide levels are located in nearby positions on chromosome 6 suggesting a tandem gene duplication event that might have caused evolution of one these receptors. Unequal number of PYLs across seven different plant species ranging from 8 to 14 (sorghum and Arabidopsis) identified in the current study.

We analysed ABA receptors in seven members of grass family. Phylogenetic analysis of PYL protein sequences from rice, maize, sorghum, barley, brachypodium, wheat and foxtail millet showed that PYL gene family across seven species can be broadly grouped into three subfamilies similar to that in Arabidopsis (Fig. 3). Subfamily II has the maximum number of PYL members (41), followed by Subfamily III (31 PYL genes) and Subfamily I has the least members (29 PYL genes). These members of grass family analysed here have evolved from a common ancestor about 96 Ma, and BOP (Bambusoideae-Oryzoideae-Pooideae) and PACCMAD (Panicoideae, Arundinoideae, Chloridoideae, Centothecoideae, Aristidoideae, Danthonioideae) clades diversified about 70 Ma. Arabidopsis and rice which diverged from a common ancestor about 120–200 Ma have similar number of PYLs. It is interesting to note that rice (BOP clade) and maize (PACCMAD clade) have 13 PYLs each, while other members of BOP (wheat – each diploid genome, barley and *Brachypodium*) and PACCMAD (sorghum and foxtail millet) have only 8–9 PYLs, respectively. The sub-family III of all seven members of grass family analysed here have 3 PYLs (PYL1-PYL3), while it varied among grasses in sub-family II (4–7 PYLs) and family I (2–7 PYLs). The conservation of three subfamilies *albeit* with different number of members suggests non-redundant roles for each subfamily.

Collinearity results showed that approximate 181 (Additional file 12 Table S3) homologous gene pairs existed among PYLs from *A. thaliana*, *B. distachyon*, *O. sativa*, *Z. mays*, *S. bicolor* and *H. vulgare* at genome wide scale (Fig. 4), grouping PYL genes into 5 clusters with each cluster depicting high sequence similarity and might therefore share same functional domains. Surprisingly PYLs in cluster 5 belonged to subfamily III to phylogenetic classification. Synteny analysis of PYL family between Arabidopsis and rice showed that collinearity blocks between PYL members of subfamily II were highest, while members of subfamily III formed least collinearity pairs (Fig. 5). These results suggest that PYL family expanded through segmental duplication events during evolution. The evolutionary history subfamily II members might provide more clues to the origin and evolution of the *PYL* gene family.

Non synonymous SAPs in OsPYLs identified from 3 K SNP seek database showed that frequency of SAPs was high across *indica* rice cultivars as compared to landraces (Table 3). Moreover most of the SAPs were present across START domain that might modulate the ABA binding ability of ABA receptors in response to different types and magnitude of stresses.

### Regulation of gene expression

We carried out a systematic analysis of CREs in promoter regions of PYLs and identified various types of CREs such as stress responsive elements, hormone responsive elements, metabolic responsive elements and elements involved in growth and development (Additional file 10). The number of ABA and stress responsive elements was found to be highest in the promoters across *OsPYL* genes. Most *OsPYL* promoters contain CREs stress signalling and pathogen response. Each promoter of *OsPYL* genes possessed more than one *cis* elements involved in photosynthesis and light responsiveness, while elements involved in carbohydrate and starch metabolism was equally high across all promoters. This suggests that *OsPYL* genes might be regulated by photosynthesis and carbohydrate metabolism signalling. The knowledge of presence of regulatory elements in the promoters of *PYL* genes can further help in functional characterization and tailoring PYL receptors in rice and other commercially important crops.

We identified 32 candidate miRNAs that may potentially target *OsPYLs* mRNA to regulate their expression in rice (Fig. 6a). Most of these miRNAs identified have been implicated in stress responsiveness and development (Additional file 8). In an earlier study with *Brassica napus*, 26 miRNAs targeting 11 *BnPYL* genes were identified, and predicted that 10 members of miR169 target *BnPYL1–4* [85]. For better understanding, each *OsPYL* gene targeted by different miRNAs is depicted by different colour and shape (Fig. 6c). In our study, many *PYLs* were targeted by multiple miRNAs with maximum of 8 different miRNAs targeting *OsPYL2*. Although *OsPYL8* and *OsPYL9* have very high sequence similarity, *OsPYL9* is targeted by 7 miRNAs while *OsPYL8* is targeted by only 4 miRNAs. The miR5075 targets five *OsPYL* genes and thus may play a critical role in fine tuning the expression of PYLs. In silico expression analysis of the identified miRNAs under different tissues and abiotic stresses revealed that apart from osa-miR820a and osa-miR408-3p, other miRNAs were downregulated (Fig. 6b). It is important to note that ABA-activated SnRK2 kinases interact with and phosphorylate SERRATE (SE) and HYPONASTIC LEAVES (HYL1) proteins involved in miRNA biogenesis [86]. The predicted miRNA mediated regulation of PYLs suggests that miRNA mediated regulation of PYLs may be important not only for stress response of rice but may also play a key role in feedback regulation of overall miRNA biogenesis through PYL-mediated regulation of SnRK2.2/3/6. Further functional characterization of the predicted miRNAs would enable us to better understand the regulatory mechanism underlying ABA receptors and miRNA biogenesis.

Analyzing the spatiotemporal pattern of gene expression across the broad spectrum of different tissues, developmental stages and stress conditions would provide insight into the physiological and developmental functions of *OsPYLs*. In silico as well as q-RT-PCR expression analysis of *OsPYLs* different stress treatments and developmental stages showed that *PYL* genes are regulated in a tissue and developmental dependent manner and by multiple stresses (Fig. 10, 11, 12). Two members of subfamily II (*OsPYL5* and *OsPYL6*) and all 3 members of subfamily III showed higher expression potential in different tissues across developmental stages, while only *OsPYL10* and *OsPYL11* among the 7 members of subfamily I showed higher expression potential in different tissues across developmental stages (Fig. 9). This suggests that these PYLs have multiple roles throughout the growth and development of rice. The *PYL8* and *PYL9* orthologs in Arabidopsis have been shown to play important role in lateral root formation during seedling growth [5]. Interestingly, *OsPYL7*, *OsPYL8* and *OsPYL9* showed expression during germination stage and seed development (embryo, endosperm and caryopsis) (Fig. 9). Real-time qRT-PCR analysis also showed that *OsPYL8* and *OsPYL9* were highly expressed in panicles and seeds (Fig. 11). In a previous study also it was found that these two PYLs were highly expressed in seeds [42]. These results suggest that *OsPYL7*, *OsPYL8* and *OsPYL9* may have specific role in germination as well geed development. Although *OsPYL7* was predicted as non-functional due to lack of C-terminal sequences (motif2), it was found to be co-expressed with *OsPYL8* and *OsPYL9* in seeds (embryo, endosperm and caryopsis) (Fig. 9). Further studies may illuminate whether *OsPYL7* interact *OsPYL8* and *OsPYL9* to regulate rice seed development. In our qRT-PCR expression analysis also *OsPYL8* and *PYL9* showed very high levels of expression in panicle and seeds as compared with other tissues (Fig. 11). Notably we also identified CREs 2SSEEDPROTBANAPA and − 300 CORE, which are involved in seed and endosperm specific expression, specifically present in promoter region of *OsPYL8* and *OsPYL9*. This further strengthens the proposed roles of *OsPYL8* and *OsPYL9* in seed and endosperm specific activity. *OsPYL5, OsPYL9, OsPYL11* and *OsPYL13* may play an important role in regulation of source activity as their expression was higher in flag leaf which contributes to > 70% of current assimilation for grain development.

A previous study showed that only *OsPYL6* (their nomenclature, *OsPYL4*) was upregulated, while *OsPYL2*, *OsPYL3*, *OsPYL4* and *OsPYL10* were downregulated by 200 μM in 14 days old seedlings of *japonica* cv. Nipponbare [42]. In contrast, our analysis showed that 100 μM ABA significantly upregulated *OsPYL1*, *OsPYL8* and *OsPYL10* root, and *OsPYL1*, *OsPYL2*, *OsPYL5*, *OsPYL7* and *OsPYL12* in shoots of drought tolerant *indica* rice cv. Nagina22 (Fig. 12). In consistent with Tian et al. [42], in our study also ABA downregulated *OsPYL2*, *OsPYL3* and *OsPYL4* roots, *OsPYL4* and *OsPYL10* in shoots. Thus, ABA-mediated regulation of *OsPYLs* appears to be genotype and tissue dependent.

Previous functional validation studies have shown that constitutive/stress-inducible overexpression of *OsPYL2* [42], *OsPYL10* [42, 47, 87], and *OsPYL11* (=RCAR5) [44] conferred tolerance to abiotic stresses. In a previous study, expression analysis of *OsPYL11* (=*RCAR5*) [44] under salt, PEG and ABA treatments showed that it is significantly downregulated by ABA and salt stress. In our analysis PEG downregulated the expression of *OsPYL11* in root, while salt stress upregulated in shoots of drought and heat tolerant cv. Nagina 22. In a previous study, *OsPYL10* [47] expression was found to be downregulated by PEG, NaCl and cold stresses in the roots, but was found to be upregulated by ABA in roots. In this study also similar expression of *OsPYL10* was found under these treatments. However, in none of the previous studies expression of all *OsPYLs* under different stresses were examined.

In this study, qRT-PCR expression analysis showed that all the *OsPYLs* are regulated by one or more abiotic stresses (osmotic/PEG, drought, salt, heat and cold) in at least one tissue/development stage of rice plant (Fig. 12, 13). This suggests that all *PYLs* play important roles in abiotic stress responses of rice. In general subfamily I and III *PYLs* were upregulated by different abiotic stresses and ABA at seedling stage of drought and heat tolerant rice cv. Nagina 22, while subfamily II *PYLs* were downregulated (Fig. 12, 13). This suggests that subfamily-wise stress response role at seedling stage.

In consistent with the presence of CCAATBOX1 [88] CRE that act as heat responsive elements in the Promoters of PYLs, heat stress upregulated OsPYLs except OsPYL6 and OsPYL11, at least in one tissue (Fig. 13). Over all, drought stress downregulated all PYLs except *OsPYL4* which was upregulated and *OsPYL12* which was unaltered, while heat stress upregulated *OsPYL1*, *OsPYL3 OsPYL8*, *OsPYL9*, *OsPYL12* and *OsPYL12* in the roots at reproductive stage (Fig. 13). This suggests contrasting regulation and function of OsPYLs in roots under drought and heat stress. Both drought and heat stress upregulated *OsPYL2*, *OsPYL3 OsPYL4*, *OsPYL5*, *OsPYL8*, *OsPYL9* and *OsPYL12* in the stem at reproductive stage, while *OsPYL1*, *OsPYL6* and *OsPYL11* were upregulated only by drought and *OsPYL10* was upregulated only by heat in the stems (Fig. 13). Interestingly all the three OsPYLs (*OsPYL2*, *OsPYL4* and *OsPYL13*) upregulated in flag leaf in response to drought stress were also upregulated under heat stress. In the panicle only *OsPYL13* was commonly upregulated by both drought and heat at reproductive stage. *OsPYL8* and *OsPYL9* were specifically upregulated by drought but were downregulated by heat in panicles, while *OsPYL3* which was upregulated under heat but was downregulated under drought stress in panicle (Fig. 13). The diverse expression patterns of *OsPYL*s were indicative of their functional distinctiveness.

## Conclusion

The present study is a comprehensive functional identification and characterization of *PYL* gene family in indica rice at genomic level. Evolutionary relationship of *PYL* genes in rice and other cereal crops was established which grouped *PYL* genes into three subfamilies that are structurally and functionally evolutionarily conserved. Identified *cis* elements could help in understanding the diversified role of PYL receptors in response to different stresses and developmental stages. These data will provide the basis for understanding evolutionary history and the developmental roles of *OsPYL* genes in rice, and may be helpful for future exploration of the biological functions of *OsPYL* genes. These findings will also serve to extend our knowledge for identifying candidate genes that improve plant architecture under stress conditions and enable potential breeding and genetic improvements for other agriculture crops.

## Methods

### Identification of *PYL* genes from plant species

To identify ABA receptors of different species, Arabidopsis PYLs were used as query in EnsemblPlants database (https://plants.ensembl.org/index.html) against their respective species genome with a threshold of 10^− 4^ and Match/Mismatch score of 2 and − 3.

### Chromosomal distribution of *PYL* genes in rice

Chromosomal location of *OsPYL* genes was determined with respect to their position and information retrieved from rice genome sequences. The physical map information on chromosome number, length and gene loci were obtained from rice genome annotation project (RGAP) (http://rice.plantbiology.msu.edu/). Elementary physical map depicting the location and distribution of *OsPYL* gene family was drawn using Map Tool software from Oryza base (https://shigen.nig.ac.jp/rice/oryzabase/) with default parameters.

### Sequence retrieval and phylogenetic analysis

Gene sequences of rice *PYL*s were identified from the Rice Genome Annotation Project [68] using Arabidopsis PYL protein sequences from Arabidopsis Information Resource [89]. In the present study, Nagina22 sequence for *OsPYL7* and *OsPYL13* were retrieved from Rice SNP-Seek Database (https://snp-seek.irri.org/), while rest of the *PYLs* were coned and sequenced from Nagina 22. Sequences of PYLs from Brachypodium, Sorghum, barley, maize, foxtail millet and wheat genomes were retrieved from EnsemblPlants database (https://plants.ensembl.org/index.html). For phylogenetic analysis, amino acid sequences of putative PYL proteins of *H. vulgare*, *S. bicolor*, *O. sativa* and *A. thaliana, T. aestivum, Z. mays, S. italica* and *B. distachyon* were analyzed. The genes of *PYL*s were named based on numbering and sequence homology with *A. thaliana* orthologs genes. Multiple sequence alignment was executed by ClustalW 2.0 program [90]. Phylogenetic trees were constructed using MEGA X [70] by the maximum likelihood method [69].

### Analysis of gene structure, conserved motif and protein properties

Conserved motifs were also predicted for all 13 OsPYL proteins using MEME Suite v5.1.0 [91, 92]. Functional annotations of these motifs were performed using HHpred (http:// toolkit.tuebingen.mpg.de/hhpred) [93]. Maximum number of motifs was specified as 10. Parameters on minimum/maximum width were specified as 6 and 50, while the range for minimum and maximum sites per motif were kept as 2 and 13, respectively. The motifs were serially numbered according to their frequency of occurrence in MEME. Motifs were placed adjacent to their respective OsPYLs in accordance with their subfamily signatures based on phylogenetic relationship. Using the ExPASy database, the isoelectric points and molecular weights of the OsPYLs were predicted.

### miRNA identification and in silico expression analysis

miRNA targeting *PYL* genes in rice were predicted using psRNATarget [73] against all the rice mature miRNAs that were reported in miRbase [74] and the network was created using Cytoscape [94]. In silico expression analysis of identified miRNAs was carried out using miRid as query against rice datasets of plant miRNA expression atlas database PmiRExAt (http://pmirexat.nabi.res.in/index.html) [95].

### Genome wide collinearity and Ka/Ks analysis of PYLs

Analysis of homologous gene pairs of PYLs among *Arabidopsis thaliana, Oryza sativa, Zea mays, Brachypodium distachyon, Sorghum bicolor and Hordeum vulgare* at genome level was carried out. Whole genome primary transcript file of each species was obtained from Phytozome database (https://phytozome.jgi.doe.gov/pz/portal.html) and whole genome reciprocal protein to protein BLAST in all pair wise combinations (36 permutation combinations) was carried out. BLAST result of all the possible combinations was merged. Annotation file of all selected species were imported into Cytoscape and collinearity network was constructed. Similarly for Synteny block calculation of *PYL* gene family between Arabidopsis and rice at genome scale were used. *PYL* genes were filtered form whole genome collinearity data along with gene positional information of gene was detected using ‘collinearity with gene families’ were queried in MCScanX and visualized in CIRCOS [96, 97] using default parameters. The values of nucleotide substitution parameter Ka (non-synonymous) and Ks were calculated for PYL gene family in *Oryza sativa*. Orthologs of rice PYL gene family were noted from collinearity blocks table and calculated using MCScanX software [96]. Pairwise global alignment for all ortholog pairs (protein sequences) was done online by EMBOSS Needle Pairwise Sequence Alignment tool [98]. Online program PAL2NAL [99] was used to convert protein sequence alignment and the corresponding mRNA sequences into a codon alignment and calculating Ka/Ks value from the aligned codon.

### Identification of SNPs in *PYL* genes

Amongst available 3024 accessions, sequence of 13 *PYL* genes from 12 mega rice varieties comprising of contrasting genotypes in terms of abiotic stress sensitivity were fetched from Rice SNP-Seek database [76] against the reference Nipponbare sequence. Non-synonymous SNPs that could particularly translate to change in the protein sequence were identified across the selected genotypes. Domain based localization of SAPs was done on the basis of secondary structure of OsPYL proteins.

### Identification of putative *cis*-regulatory elements (CREs) in the promoters

The 2000 bp upstream sequences from the translation start site of all of the *OsPYL* genes were obtained from Rice genome annotation project database. The putative *cis*-acting regulatory elements in these sequences were predicted using the NewPlace web server [77] and then to identify the putative CREs. Functional annotation of individual CRE was manually curated from place.seq (https://www.dna.affrc.go.jp/PLACE/place_seq.shtml); (Additional file 10).

### Expression analysis of *OsPYL*s in rice

In silico expression analysis of ABA receptors at different developmental stages and stresses were analyzed using GENEVESTIGATOR database (https://genevestigator.com/gv/) [82]. The rice genotype Nagina 22 (*Oryza sativa ssp. indica* cv. Nagina 22) seeds, from our lab in Division of Plant Physiology, ICAR-IARI, New Delhi, was used for analysis of tissue specific and stress responsive expression of 13 *OsPYL* genes at seedling stage (14 day old) and reproductive stage using real time qRT-PCR. *OsPYL* expression was analyzed in different tissues from plants at anthesis stage exposed to control, drought and heat stress treatments. At seedling stage, plants grown in Yosida’s medium (YM) were treated with YM supplemented with 20% PEG 6000 (− 0.49 MPa), 200 mM NaCl (− 1.01 MPa; 20 dS/m) and cold (4 °C) stresses, Heat (42 °C) and 100 μM ABA for 6 h. Samples were collected from control, and treated plants and frozen in liquid nitrogen. Drought and heat stress was imposed at anthesis stage in Nagina22 grown in pot at green house, IARI, New Delhi conditions of 30 + 2 °C for control and drought while temperature of 42 + 2 °C for heat at a relative humidity (RH) of ~ 60–70%. Drought was imposed at reproductive stage by with-holding water till the soil matric potential (SMP) reached up to -80 kPa.

### Quantitative real-time RT-PCR

Total RNA was extracted using RNeasyMini Kit (Qiagen, Germany) following manufacturers protocol and treated with DNaseI. Quantification of RNA was carried out using NanoDrop (Thermo Fisher, US). cDNA was synthesized from 2 μg of total RNA using Superscript III Reverse Transcriptase (Invitrogen). Real-time PCR was performed with Hotstart SYBR Green master mix (KAPA SYBR FAST; Universal). PCR conditions were 95 °C for 30 s, followed by 40 cycles of 95 °C for 5 s and 60 °C for 40 s in StepOne Real-Time PCR system (Applied Biosystems). The relative expression levels of *OsPYL* genes were calculated based on the comparative Ct method using the 2^^ΔΔ^Ct method [100] and all expressions were normalized against the Ubiquitin5 gene [101]. Root tissue ΔCt of seedling stage was used as calibrator for tissue specific expression analysis, while expression level control tissue (ΔCt) was used as calibrator for stress responsive expression analysis. The primers used are listed in (Additional file 13 Table S4).

### Statistical analysis

The presented values are the means ± SE of three different experiments with three replicated measurements. Unpaired t-Test was used to compare significant differences based on Fisher’s LSD test at significance levels of *P* < 0.05 and *P* < 0.01 using GraphPad Prism software (GraphPad Software Inc., La Jolla, CA, USA).

## Supplementary information

**Additional file 1 **Complete CDS sequences of 13Nagina22 *OsPYLs*.

**Additional file 2.** Sequence of 13 Nagina22 OsPYL proteins.

**Additional file 3 : Table S1.** Putative function of Motifs identified.

**Additional file 4.** Protein sequence of identified PYLs of eight species.

**Additional file 5 : Table S2.** Pair wise collinearity among PYLs of Arabidopsis and rice.

**Additional file 6 : Figure S1.**Synteny blocks between Arabidopsis and rice at genomic level.

**Additional file 7.** Ka/Ks values of PYL orthologs.

**Additional file 8 **Sequence and detail of identified miRNAs targeting *OsPYLs*.

**Additional file 9 : Figure S2.** Sequence alignment of 13 OsPYL proteins depicting four conserved loops CL1–CL4.

**Additional file 10 **List of identified CRE and their putative function in 13 *OsPYL* promoter.

**Additional file 11 : Figure S3.** Frequency and distribution of identified CRE in individual *OsPYL* promoter.

**Additional file 12 : Table S3.** List of homologous gene pair between Arabidopsis and rice.

**Additional file 13 : Table S4.** List of primers used for q-RT expression analysis of 13 OsPYL genes.

## Data Availability

The sequences of ABA receptors cloned from rice cv. Nagina 22 were deposited in the NCBI and are available in the NCBI (https://www.ncbi.nlm.nih.gov/nucleotide/; Table 1; Additional file 1). The rice genome sequences, the physical map information on chromosome number and length and gene loci are available in RGAP (http://rice.plantbiology.msu.edu/), the Nagina22 sequence for OsPYL7, OsPYL12 and OsPYL13, the sequence of thirteen PYL genes from twelve mega rice varieties, the reference Nipponbare sequence are available SNP-seek (https://snp-seek.irri.org/, Table 1, Additional file 1), and the sequences of PYLs from Arabidopsis (https://www.arabidopsis.org) Brachypodium, Sorghum, barley, maize, foxtail millet and wheat genomes (https://plants.ensembl.org/index.html) are available in the respective database. The accession number and web links for the PYLs of these species are given in Additional file 4.

## Abbreviations

ABA: Abscisic acid
MW: Molecular weight
NJ: Neighbor-joining
ORF: Open reading frame
pI: isoelectric point
PYL: PYR1-Like
PYR: Pyrabactin Resistance
RCAR: Regulatory Component of ABA Receptor
SNP: Single nucleotide polymorphism
SAP: Single amino-acid polymorphism
CRE: *cis* regulatory elements
