# Supplementary material for: Genome-wide identification and characterization of ABA receptor PYL gene family in rice
Source: BMC Genomics. 2020 Sep 30;21:676. doi: 10.1186/s12864-020-07083-y (PMC7526420; doi:10.1186/s12864-020-07083-y)
Supplement: Supplementary file 1 — Additional file 1 Complete CDS sequences of 13Nagina22 OsPYLs. [file 12864_2020_7083_MOESM1_ESM.docx]

**Additional File 1.** **Nagina22 PYL receptor-CDS sequence**

The sequences of ABA receptors cloned from rice cv. Nagina 22 were deposited in the NCBI and are available in the NCBI https://www.ncbi.nlm.nih.gov/nucleotide/. The Nagina22 sequence for OsPYL7, OsPYL12 and OsPYL13 are available in the SNPseek data base https://snp-seek.irri.org/.

>OsPYL1_LOC_Os10g42280 (Gene Bank Acc. No. KJ634481)

ATGGAGCAGCAGGAGGAAGTGCCACCGCCGCCGGCGGGGCTGGGGCTGACGGCGGAGGAGTACGCGCAGGTGCGGGCGACGGTGGAGGCGCACCACCGCTACGCCGTGGGGCCGGGCCAATGCTCCTCCCTCCTCGCGCAGCGCATCCACGCGCCGCCCGCCGCCGTCTGGGCCGTCGTCCGCCGCTTCGACTGCCCCCAGGTGTACAAGCACTTCATCCGCAGCTGCGTCCTCCGGCCCGACCCCCACCACGACGACAACGGCAACGACCTCCGCCCCGGCCGCCTCCGCGAGGTCAGCGTCATCTCCGGCCTCCCCGCCAGCACCAGCACCGAGCGCCTCGACCTCCTCGACGACGCCCACCGCGTCTTCGGCTTCACCATCACCGGCGGCGAGCACCGCCTCCGCAACTACCGATCCGTCACCACCGTCTCCCAGCTCGACGAGATCTGCACCCTCGTCCTCGAGTCCTACATCGTCGACGTCCCCGACGGCAACACCGAGGACGACACCCGCCTCTTCGCCGACACCGTCATCAGGCTCAACCTCCAGAAGCTCAAGTCCGTCTCCGAGGCCAACGCCAACGCCGCTGCCGCCGCCGCCGCTCCTCCTCCTCCTCCACCGGCGGCGGCGGAATAG

>OsPYL2_LOC_Os06g36670 (Gene Bank Acc. No. KJ634482)

ATGGAGGCGCACGTGGAGAGGGCGCTCCGGGAGGGCCTGACGGAGGAGGAGAGGGCGGCGCTGGAGCCGGCGGTGATGGCGCACCACACGTTCCCGCCCTCCACCACCACCGCCACCACGGCGGCGGCAACGTGCACGTCGCTGGTGACGCAGCGCGTGGCGGCGCCGGTGCGCGCGGTGTGGCCCATCGTGCGCAGCTTCGGCAACCCGCAGCGGTACAAGCACTTCGTCCGCACCTGCGCCCTCGCCGCCGGCGACGGCGCCAGCGTCGGCAGCGTCCGCGAGGTCACCGTCGTGTCCGGCCTCCCGGCTTCCACCAGCACCGAGCGCCTCGAGATGCTCGACGACGACCGCCACATCATCAGCTTCCGCGTCGTCGGCGGCCAGCACCGCCTCCGCAACTACCGCTCCGTCACCTCCGTCACCGAGTTCCAGCCGCCCGCCGCCGGCCCCGCCCCCGCCCCGCCGTACTGCGTCGTCGTCGAGTCCTACGTCGTCGACGTCCCCGACGGGAACACGGCGGAGGACACCAGGATGTTCACCGACACCGTCGTCAAGCTCAACCTCCAGAAGCTCGCCGCCGTCGCCGAGGACTCCTCCTCTGCTTCGCGGCGGCGCGACTAG

>OsPYL3_LOC_Os02g13330 (Gene Bank Acc. No. KM371729)

ATGGAGCCCCACATGGAGAGGGCGCTGCGGGAGGCGGTGGCGTCGGAGGCGGAGCGGCGGGAGCTGGAGGGCGTGGTGCGCGCGCACCACACGTTCCCGGCGGCGGAGCGCGCGGCGGGGCCGGGGCGGCGGCCGACGTGCACGTCGCTGGTGGCGCAGCGGGTGGACGCGCCGCTCGCCGCCGTGTGGCCCATCGTGCGCGGGTTCGCCAACCCGCAGCGGTACAAGCACTTCATCAAGTCGTGCGAGCTCGCCGCCGGGGACGGCGCCACCGTGGGGAGCGTCCGGGAGGTCGCCGTCGTGTCGGGCCTCCCGGCGTCCACCAGCACCGAGCGCCTCGAGATCCTCGACGACGACCGCCACGTCCTCAGCTTCCGCGTCGTCGGCGGCGACCACCGCCTCCGCAACTACCGCTCCGTCACCTCCGTCACCGAGTTCTCCTCCCCCTCCTCGCCGCCGCGGCCGTACTGCGTCGTCGTCGAGTCCTACGTCGTCGACGTCCCCGAGGGGAACACCGAGGAGGACACGCGCATGTTCACCGACACCGTCGTCAAGCTCAACCTCCAGAAGCTCGCCGCCGTCGCCACCTCCTCCTCGCCGCCGGCCGCCGGCAACCACCATTAG

>OsPYL4_LOC_Os01g61210 (Gene Bank Acc. No. KJ634480)

ATGCCGTACGCCGCCGTACGTCCTTCGCCACCGCCGCAGCTCAGCCGGCCGATCGGCTCCGGAGCCGGCGGCGGTAAGGCGTGCCCGGCGGTGCCGTGCGAGGTGGCGCGGTACCACGAGCACGCGGTGGGCGCGGGGCAGTGCTTCTCCACCGTGGTGCAGGCGATCGCGGCGCCGGCGGACGCGGTGTGGTCGGTGGTGCGGAGGTTCGACCGGCCGCAGGCGTACAAGAAGTTCATCAAGAGCTGCCGCCTCGTGGACGGCGACGGCGGCGAGGTCGGGTCGGTGCGGGAGGTGCGCGTCGTGTCCGGGCTGCCGGCCACGAGCAGCCGCGAGCGGCTCGAGGTCCTGGACGACGACCGCCGCGTGCTCAGCTTCCGGATCGTCGGCGGCGAGCACCGCCTCGCCAACTACCGCTCGGTGACCACCGTCCACGAGGCGGCGGCGCCGGCGATGGCCGTGGTGGTCGAGTCGTACGTGGTGGACGTGCCGCCGGGGAACACCTGGGAGGAGACGCGCGTGTTCGTGGACACGATCGTGCGCTGCAACCTCCAGTCGCTTGCGCGAACGGTCGAGCGGCTCGCGCCGGAAGCGCCGCGCGCCAACGGATCGATCGATCATGCATGA

>OsPYL5_LOC_Os05g39580 (Gene Bank Acc. No. KJ634479)

ATGATGCCGTACACCGCTCCACGGCCGTCGCCGCCGCAGCACAGCCGGATCGGGGGCTGTGGCGGTGGGGGAGTGTTGAAGGCGGCGGGGGCGGCGGGGCACGCGGCGTCGTGCGTGGCGGTGCCGGCGGAGGTGGCGAGGCACCACGAGCACGCGGCGGGCGTGGGGCAGTGCTGCTCGGCGGTGGTGCAGGCGATAGCGGCGCCGGTGGACGCGGTGTGGTCGGTGGTGCGGCGGTTCGACCGGCCGCAGGCGTACAAGCACTTCATCCGGAGCTGCCGCCTCCTGGACGGCGACGGCGACGGCGGCGCGGTGGCGGTGGGGTCGGTGCGGGAGGTGCGGGTGGTGTCGGGCCTCCCCGCCACCAGCAGCCGCGAGCGGCTGGAGATCCTCGACGACGAGCGCCGCGTGCTCAGCTTCCGCGTCGTCGGCGGCGAGCACCGCCTCTCCAACTACCGGTCGGTCACCACCGTGCACGAGACGGCGGCCGGCGCCGCCGCCGCCGTCGTCGTGGAGTCCTACGTGGTGGACGTGCCCCACGGCAACACCGCCGACGAGACCCGCATGTTCGTCGACACCATCGTCCGGTGCAACCTCCAGTCGCTGGCACGCACCGCCGAGCAGCTCGCCCTCGCCGCGCCGCGCGCCGCCTAG

>OsPYL6_LOC_Os03g18600 (Gene Bank Acc. No. KJ634478)

ATGCCGTGCATCCCGGCGTCCAGCCCTGGCATCCCGCACCAGCACCAGCACCAGCACCACCGGGCGCTAGCAGGCGTCGGCATGGCGGTCGGGTGCGCGGCGGAGGCGGCCGTGGCCGCGGCGGGTGTCGCGGGGACGAGGTGCGGGGCGCACGACGGGGAGGTGCCTATGGAGGTGGCGCGGCACCACGAGCACGCGGAGCCAGGGTCGGGGCGGTGCTGCTCCGCGGTGGTCCAGCACGTAGCGGCGCCGGCGGCGGCGGTGTGGTCGGTGGTGCGGCGGTTCGACCAGCCCCAGGCGTACAAGCGGTTCGTCCGCAGCTGCGCGCTGCTCGCCGGGGACGGCGGCGTGGGCACGCTCCGCGAGGTGCGCGTCGTGTCGGGCCTCCCCGCGGCGTCCTCCCGCGAGCGCCTCGAGATCCTCGACGACGAGAGCCACGTCCTCAGCTTCCGCGTCGTCGGCGGCGAGCACCGCCTCAAGAACTACCTCTCGGTCACCACCGTCCACCCGTCCCCGTCCGCGCCGACGGCCGCCACCGTCGTGGTGGAGTCCTACGTCGTCGACGTGCCCCCGGGCAACACGCCCGAGGACACCCGCGTGTTCGTCGACACCATCGTCAAGTGCAACCTCCAGTCTCTCGCCAAGACCGCCGAGAAGCTCGCCGCCGGCGCGAGGGCCGCCGGCTCGTGA

>OsPYL7_LOC_Os06g33480

ATGAACAGTGGCGCTGGTGGTGCTGGGGGAGCAGCAGTAGGGAGGATGCCGGCGGGGAGCCTCCAGTGGGCACAGTGGAGGCTAGCGGATGAGCGATGTGAACTCCGGGAGGAGGAGATGGAGTACATGCGACGGTTCCACCGCCACGAAATCGGTAGCAACCAGTGCAACTCCTTCATTGCGAAGCACGTTAGGGCGCCCCTCCAAAATGTTTGGTCACTGGTGAGGAGGTTTGATCAGCCACAAATTTACAAACCTTTTGTGAGAAAGTGTGTAATGCGCGGAAATGTTGAGACCGGAAGTGTTAGGGAGATCATTGTTCAAAGTGGGCTCCCAGCCACAAGGAGCATTGAAAGGTTAGAGTTCCTTGATGATAATGAATACATCCTCCGTGTCAAGTTTATTGGTGGCGATCATATGTTGAAGAAATGTGGGCCTTAG

>OsPYL8_LOC_Os06g33640 (Gene Bank Acc. No. KJ634477)

ATGAACGGCGCTGGTGGTGCGGGAGGAGCAGCGGCAGGGAAGTTGCCAATGGTGAGCCACCGACAGGTGCAGTGGAGACTAGCGGACGAGCGGTGTGAGCTCCGGGAGGAAGAGATGGAGTATATCCGGCAGTTCCACCGCCACGAGCCCAGCAGCAACCAGTGCACCTCGTTCGTCGCCAAGCATATCAAGGCGCCCCTCCAAACCGTTTGGTCACTAGTGAGGAGGTTTGATCAGCCACAACTTTTCAAACCTTTTGTGAGAAAGTGCGTAATGCGAGAAAACATTATTGCGACCGGATGTGTTAGGGAGGTCAATGTTCAAAGCGGGCTTCCAGCCACAAGGAGCACTGAGAGGTTAGAGTTGCTTGATGATAACGAACATATCCTCAAAGTCAAGTTTATTGGGGGCGATCATATGTTGAAGAATTACTCATCCATCCTAACCATCCACTCTGAGGTCATCGATGGCCAACTTGGAACATTGGTGGTCGAATCATTTGTAGTGGATATTCCAGAAGGGAACACCAAAGACGACATATGCTATTTCATCGAGAACATTCTCAGGTGCAACCTTATGACCCTTGCTGATGTGTCAGAGGAGCGCCTTGCCAATCCTTGA

>OsPYL9_LOC_Os06g33690 (Gene Bank Acc. No. KM371729)

ATGAACGGCGTTGGTGGGGCGGGAGGAGCAGCGGCAGGGAAGTTGCCAATGGTGAGCCACCGACGGGTGCAGTGGAGGCTAGCGGACGAGCGGTGTGAGCTCCGGGAGGAAGAGATGGAGTATATCCGGCGGTTCCACCGCCATGAGCCTAGTAGCAACCAGTGCACCTCGTTCGCCGCCAAGCATATCAAGGCGCCCCTCCACACCGTTTGGTCACTAGTGAGGAGGTTTGATCAGCCACAACTTTTCAAACCTTTTGTGAGAAACTGTGTAATGCGAGAAAACATTATTGCGACCGGATGTATTAGGGAGGTCAATGTTCAAAGCGGGCTTCCAGCCACAAGGAGCACTGAGAGGTTAGAGTTGCTTGATGATAATGAACACATCCTCAAAGTCAATTTTATTGGGGGAGATCATATGTTGAAGAATTACTCATCCATCCTGACCGTCCACTCTGAGGTCATCGATGGCCAGCTTGGAACACTGGTGGTCGAATCATTTATTGTGGACGTTCCAGAAGGGAACACCAAAGATGACATAAGCTATTTCATCGAGAACGTTCTCAGGTGTAACCTTAGGACCCTTGCTGATGTGTCAGAGGAGCGCCTTGCCAATCCTTGA

>OsPYL10_LOC_Os02g15640 (Gene Bank Acc. No. KF925265)

ATGGTGGAGGTGGGAGGAGGAGCGGCGGAGGCGGCGGCGGGGAGGAGGTGGCGGCTGGCGGACGAGAGGTGCGACCTGCGCGCGGCGGAGACGGAGTACGTGAGGCGGTTCCACCGCCACGAGCCCCGCGACCACCAGTGCTCCTCCGCCGTCGCCAAGCACATCAAGGCCCCCGTCCACCTGGTTTGGTCTCTGGTGAGGCGTTTTGATCAGCCACAGCTTTTCAAGCCATTTGTGAGCCGGTGTGAGATGAAAGGGAACATTGAGATTGGCAGTGTAAGGGAGGTTAATGTTAAGTCTGGCCTGCCTGCCACAAGAAGCACTGAGAGGCTGGAGCTGTTAGATGACAATGAGCACATACTCAGTGTCAGGTTCGTGGGAGGTGATCATAGGCTCAAGAATTACTCCTCCATCCTGACCGTCCACCCGGAGGTGATCGACGGCCGGCCCGGGACGCTGGTGATCGAGTCGTTCGTCGTCGACGTCCCGGAGGGGAACACCAAGGATGAGACATGCTACTTCGTGGAGGCCCTGCTGAAATGCAACCTGAAATCTCTTGCAGAGGTTTCTGAACGCCTGGTTGTCAAGGACCAAACCGAGCCCCTCGACCGGTGA

>OsPYL11_LOC_Os05g12260 (Gene Bank Acc. No. KJ634476)

ATGGTGGGGCTTGTGGGAGGAGGAGGTTGGAGGGTCGGGGATGATGCGGCGGGTGGGGGTGGGGGAGGAGCGGTGGCGGCGGGGGCTGCGGCGGCGGCGGAGGCGGAGCACATGCGGAGGCTCCACAGCCACGCCCCCGGCGAGCACCAGTGCAGCTCCGCGCTCGTCAAGCACATCAAGGCTCCTGTTCACCTCGTGTGGTCGCTGGTGCGGAGCTTCGACCAGCCGCAGAGGTACAAGCCGTTCGTCAGCCGCTGCGTCGTGCGCGGCGGCGACCTCGAGATCGGCAGCGTGCGCGAGGTCAACGTCAAGACCGGCCTCCCGGCGACCACCAGCACGGAGAGGCTCGAGCTGCTCGACGACGACGAGCACATCCTCAGCGTCAAGTTCGTCGGCGGCGACCACCGCCTCAGGAACTACTCATCCATCGTAACTGTCCATCCGGAGAGCATCGATGGAAGACCAGGGACGCTTGTGATTGAATCATTTGTGGTGGACGTGCCTGATGGAAATACAAAGGACGAGACATGCTACTTTGTCGAGGCCGTGATCAAGTGCAACTTAACATCTCTCGCCGAGGTATCAGAGCGGCTAGCAGTTCAGTCACCCACCTCGCCACTTGAACAGTAG

>OsPYL12_LOC_Os02g15620

ATGCGAGGGAGTACTAGTCTCGCCGTTGGTTGTGTGCGAGAGGTTGATTTCAAGTCGGGCTTCCCTGCCAAGAGCAGTGTAGAAAGGCTTGAGATCCTTGACGACAAGGAGCACGTCTTCGGTGTCAGGATCATTGGAGGTGACCATAGGCTCAAGAATTACTCGTCAGTGTTGACGGCCAAGCCCGAGGTCATCGACGGCGAGCCGGCGACGCTGGTGTCGGAGTCCTTCGTCGTCGACGTCCCCGAAGGGAACACCGCCGACGAGACGCGCCACTTCGTCGAGTTCCTCATCCGGTGCAACCTCAGGTCACTCGCGATGGTTTCTCAACGTCTCCTGCTTGCCCAGGGGGATCTCGCCGAGCCTCCTGCACAATGA

>OsPYL13_LOC_Os06g33490

ATGAACGGCTGTACTGGTGGTGCTGGAGGAGTGGCAGCAGGGAGGCTCCCGGCGGTGAGCCTCCAGCAGGCACAGTGGAAGCTAGTAGATGAGCGATGTGAGCTCCGGGAGGAGGAGATGGAGTACGTGCGATGGTTCCACCGCTATGAGCTCGTTGCAACCGGTGCAACTCCTTCGTTGCCAAACACATCAGGGTGCCCCTCTAAACTTGGGCTCCCATCCACAAGGAGAATTGAAAGGTTAGGGTTCCCTGATGATAATGATCACACCCTCCGTGTCAAGTTTATTGGTGGTGATCACATGCTAAAGGACTACTCATCCACCTTGATTATCCACTTGGAGGTCATCGATGGCCAGCTAGTAACACTAGTGATTGAGTCATTTGTGGTGGACATTCTAGAGGGGAACACCAAAGATGAAATAAGCTATTTTATCGAGAACTTGCTCAAGTTTAACCTAAGGACCCTCCGTGTCTGA
