# Supplementary material for: Genome-wide identification and characterization of ABA receptor PYL gene family in rice
Source: BMC Genomics. 2020 Sep 30;21:676. doi: 10.1186/s12864-020-07083-y (PMC7526420; doi:10.1186/s12864-020-07083-y)
Supplement: Supplementary file 2 — Additional file 2. Sequence of 13 Nagina22 OsPYL proteins. [file 12864_2020_7083_MOESM2_ESM.docx]

**Additional File 2. Nagina22 PYL Protein sequence**

>OsPYL1

MEQQEEVPPPPAGLGLTAEEYAQVRATVEAHHRYAVGPGQCSSLLAQRIHAPPAAVWAVVRRFDCPQVYKHFIRSCVLRPDPHHDDNGNDLRPGRLREVSVISGLPASTSTERLDLLDDAHRVFGFTITGGEHRLRNYRSVTTVSQLDEICTLVLESYIVDVPDGNTEDDTRLFADTVIRLNLQKLKSVSEANANAAAAAAAPPPPPPAAAE*

>OsPYL2

MEAHVERALREGLTEEERAALEPAVMAHHTFPPSTTTATTAAATCTSLVTQRVAAPVRAVWPIVRSFGNPQRYKHFVRTCALAAGDGASVGSVREVTVVSGLPASTSTERLEMLDDDRHIISFRVVGGQHRLRNYRSVTSVTEFQPPAAGPAPAPPYCVVVESYVVDVPDGNTAEDTRMFTDTVVKLNLQKLAAVAEDSSSASRRRD*

>OsPYL3

MEPHMERALREAVASEAERRELEGVVRAHHTFPAAERAAGPGRRPTCTSLVAQRVDAPLAAVWPIVRGFANPQRYKHFIKSCELAAGDGATVGSVREVAVVSGLPASTSTERLEILDDDRHVLSFRVVGGDHRLRNYRSVTSVTEFSSPSSPPSPPRPYCVVVESYVVDVPEGNTEEDTRMFTDTVVKLNLQKLAAVATSSSPPAAGNHH*

>OsPYL4

MPYAAVRPSPPPQLSRPIGSGAGGGKACPAVPCEVARYHEHAVGAGQCCSTVVQAIAAPADAVWSVVRRFDRPQAYKKFIKSCRLVDGDGGEVGSVREVRVVSGLPATSSRERLEVLDDDRRVLSFRIVGGEHRLANYRSVTTVHEAAAPAMAVVVESYVVDVPPGNTWEETRVFVDTIVRCNLQSLARTVERLAPEAPRANGSIDHA*

>OsPYL5

MMPYTAPRPSPPQHSRIGGCGGGGVLKAAGAAGHAASCVAVPAEVARHHEHAAGVGQCCSAVVQAIAAPVDAVWSVVRRFDRPQAYKHFIRSCRLLDGDGDGGAVAVGSVREVRVVSGLPATSSRERLEILDDERRVLSFRVVGGEHRLSNYRSVTTVHETAAGAAAAVVVESYVVDVPHGNTADETRMFVDTIVRCNLQSLARTAEQLALAAPRAA*

>OsPYL6

MPCIPASSPGIPHQHQHQHHRALAGVGMAVGCAAEAAVAAAGVAGTRCGAHDGEVPMEVARHHEHAEPGSGRCCSAVVQHVAAPAPAVWSVVRRFDQPQAYKRFVRSCALLAGDGGVGTLREVRVVSGLPAASSRERLEILDDESHVLSFRVVGGEHRLKNYLSVTTVHPSPSAPTAATVVVESYVVDVPPGNTPEDTRVFVDTIVKCNLQSLANTAEKLAAGARAAGS*

>OsPYL7

MNSGAGGAGGAAVGRMPAGSLQWAQWRLADERCELREEEMEYMRRFHRHEIGSNQCNSFIAKHVRAPLQNVWSLVRRFDQPQIYKPFVRKCVMRGNVETGSVREIIVQSGLPATRSIERLEFLDDNEYILRVKFIGGDHMLKKCGP

>OsPYL8

MNGAGGAGGAAAGKLPMVSHRQVQWRLADERCELREEEMEYIRQFHRHEPSSNQCTSFVAKHIKAPLQTVWSLVRRFDQPQLFKPFVRKCVMRENIIATGCVREVNVQSGLPATRSTERLELLDDNEHILKVKFIGGDHMLKNYSSILTIHSEVIDGQLGTLVVESFVVDIPEGNTKDDICYFIENILRCNLMTLADVSEERLANP*

>OsPYL9

MNGVGGAGGAAAGKLPMVSHRRVQWRLADERCELREEEMEYIRRFHRHEPSSNQCTSFAAKHIKAPLHTVWSLVRRFDQPQLFKPFVRNCVMRENIIATGCIREVNVQSGLPATRSTERLELLDDNEHILKVKFIGGDHMLKNYSSILTIHSEVIDGQLGTLVVESFIVDVLEGNTKDDISYFIENVLRCNLRTLADVSEERLANP*

>OsPYL10

MVEVGGGAAEAAAGRRWRLADERCDLRAAETEYVRRFHRHEPRDHQCSSAVAKHIKAPVHLVWSLVRRFDQPQLFKPFVSRCEMKGNIEIGSVREVNVKSGLPATRSTERLELLDDNEHILSVRFVGGDHRLKNYSSILTVHPEVIDGRPGTLVIESFVVDVPEGNTKDETCYFVEALLKCNLKSLAEVSERLVVKDQTEPLDR*

>OsPYL11

MVGLVGGGGWRVGDDAAGGGGGGAVAAGAAAAAEAEHMRRLHSHAPGEHQCSSALVKHIKAPVHLVWSLVRSFDQPQRYKPFVSRCVVRGGDLEIGSVREVNVKTGLPATTSTERLELLDDDEHILSVKFVGGDHRLRNYSSIITVHPESIDGRPGTLVIESFVVDVPDGNTKDETCYFVEAVIKCNLTSLAEVSERLAVQSPTSPLEQ*

>OsPYL12

MRGSTSLAVGCVREVDFKSGFPAKSSVERLEILDDKEHVFGVRIIGGDHRLKNYSSVLTAKPEVIDGEPATLVSESFVVDVPEGNTADETRHFVEFLIRCNLRSLAMVSQRLLLAQGDLAEPPAQ*

>OsPYL13

MNGCTGGAGGVAAGRLPAVSLQQAQWKLVDERCELREEEMEYVRWFHRYELVATGATPSLPNTSGCPSKLGLPSTRRIERLGFPDDNDHTLRVKFIGGDHMLKDYSSTLIIHLEVIDGQLVTLVIESFVVDILEGNTKDEISYFIENLLKFNLRTLRV*
