# Supplementary material for: Genome-wide identification and characterization of ABA receptor PYL gene family in rice
Source: BMC Genomics. 2020 Sep 30;21:676. doi: 10.1186/s12864-020-07083-y (PMC7526420; doi:10.1186/s12864-020-07083-y)
Supplement: Supplementary file 3 — Additional file 3 : Table S1. Putative function of Motifs identified. [file 12864_2020_7083_MOESM3_ESM.docx]

**Additional File 3: Table S1** Similarity of motifs identified by MEME analysis in 10 *OsPYL*s with the known protein domains as analyzed by HHPred analysis.

| **Motif** | **Hit (PDB id)** | **Prob.** | **E-value** | **SS** | **Target Length** |
| --- | --- | --- | --- | --- | --- |
| **Motif 1** | MLP-like protein 28 [Arabidopsis thaliana] | 98.2 | 1.3e-7 | 5.7 | 288 |
|  | SlPYL1; ABA, RECEPTOR, SIGNALING, STRESS, Signaling; 1.65A {Solanum lycopersicum} | 97.5 | 3.1e-5 | 6.5 | 232 |
|  | Polyketide cyclase/dehydrase and lipid transport superfamily protein [Arabidopsis thaliana] | 97.3 | 2.7e-5 | 3.7 | 215 |
| **Motif 2** | SlPYL1; ABA, RECEPTOR, SIGNALING, STRESS, Signaling; 1.65A {Solanum lycopersicum}; Related PDB entries: 5MOB_A 5MMQ_B 5MMQ_A 5MMX_B 5MMX_A 5MN0_A | 95.8 | 0.0056 | 4.2 | 232 |
|  | Abscisic acid receptor PYL5; abscisic acid receptor, PP2C, HORMONE; HET: GOL; 2.65A {Arabidopsis thaliana}; Related PDB entries: 4JDL_B 4JDL_C | 94.6 | 0.082 | 4.8 | 223 |
|  | Polyketide cyclase/dehydrase and lipid transport superfamily protein [Arabidopsis thaliana] | 92.8 | 0.52 | 4.7 | 203 |
| **Motif 3** | SlPYL1; ABA, RECEPTOR, SIGNALING, STRESS, Signaling; 1.65A {Solanum lycopersicum}; Related PDB entries: 5MOB_A 5MMQ_B 5MMQ_A 5MMX_B 5MMX_A 5MN0_A | 98.7 | 1.1e-9 | 6.9 | 232 |
|  | Regulatory components of ABA receptor 3 [Arabidopsis thaliana] | 98.49 | 1.3e-8 | 6.6 | 157 |
|  | Protein phosphatase 2C 37 (E.C.3.1.3.16); ABA receptor/phosphatase, HYDROLASE-RECEPTOR complex; HET: MG; 2.382A {Arabidopsis thaliana}; Related PDB entries: 4N0G_C | 98.3 | 9.4e-8 | 6.6 | 164 |
| **Motif 4** | Hypothetical protein AT4G23885 [Arabidopsis thaliana] | 48.0 | 42 | 1.5 | 77 |
|  | Ribonuclease III family protein [Arabidopsis thaliana] | 22.6 | 160 | 1.1 | 170 |
|  | Alginate lyase A1-II'; alginate lyase, polysaccharide lyase family-7; HET: SO4; 1.0A {Sphingomonas sp. A1} SCOP: b.29.1.0, l.1.1.1; Related PDB entries: 2Z42_A 2ZA9_A 2ZAA_A 2ZAB_A 2ZAC_A | 20.2 | 78 | -0.7 | 237 |
| **Motif 5** | Ribosome; FUSIDIC ACID GDP, CHIMERIC HYBRID; HET: 5OH, GDP, UAL, KBE, FUA, DPP; 3.5A {Thermus thermophilus}; Related PDB entries: 4V9M_D4 4V9M_B4 4V9J_D4 4V9J_B4 4V9K_D4 4V9K_B4 4W29_D4 4V9L_B4 4W29_B4 | 62.4 | 10 | 0.4 | 35 |
|  | DNA replication licensing factor MCM6; DNA replication, Pre-RC, Mcm6, Cdt1; NMR {Homo sapiens}; Related PDB entries: 2LE8_A | 61.4 | 6.9 | -0.1 | 114 |
|  | 50S ribosomal protein L2, chloroplastic; Chloroplast, translation, ribosome; HET: MG; 3.0A {Spinacia oleracea} | 49.3 | 24 | 0.4 | 66 |
| **Motif 6** | Late embryogenesis abundant (LEA) hydroxyproline-rich glycoprotein family [Arabidopsis thaliana] | 71.1 | 9.6 | 1.4 | 227 |
|  | Protein kinase superfamily protein [Arabidopsis thaliana] | 64.0 | 18 | 1.5 | 683 |
|  | Downstream target of AGL15-4 [Arabidopsis thaliana] | 64.0 | 18 | 1.6 | 319 |
| **Motif 7** | Fatty acid/sphingolipid desaturase [Arabidopsis thaliana] | 70.8 | 5 | 0.4 | 449 |
|  | Fatty acid desaturase family protein [Arabidopsis thaliana] | 70.5 | 4.5 | 0.2 | 299 |
|  | Delta 9 desaturase 1 [Arabidopsis thaliana] | 68.4 | 3.5 | -0.4 | 305 |
| **Motif 8** | Proline iminopeptidase [Arabidopsis thaliana] | 58.1 | 13 | 0.3 | 329 |
|  | hypothetical protein AT5G40981 [Arabidopsis thaliana] | 26.9 | 110 | 0.5 | 31 |
|  | low-molecular-weight cysteine-rich 61 [Arabidopsis thaliana] | 25.0 | 120 | 0.5 | 78 |
| **Motif 9** | senescence-associated family protein, putative (DUF581) [Arabidopsis thaliana] | 21.4 | 130 | 0.3 | 162 |
| **Motif 10** | ankyrin repeat family protein [Arabidopsis thaliana] | 32.5 | 60 | 0.4 | 683 |
|  | hypothetical protein AT5G37715 [Arabidopsis thaliana] | 22.6 | 120 | 0.4 | 130 |
|  | TYPE III EXPORT PROTEIN PSCE; VIRULENCE, CHAPERONES, COILED COIL, NEEDLE; 2.0A {PSEUDOMONAS AERUGINOSA} | 22 | 150 | 0.6 | 70 |
