# Supplementary material for: Genome-wide identification and characterization of ABA receptor PYL gene family in rice
Source: BMC Genomics. 2020 Sep 30;21:676. doi: 10.1186/s12864-020-07083-y (PMC7526420; doi:10.1186/s12864-020-07083-y)
Supplement: Supplementary file 4 — Additional file 4. Protein sequence of identified PYLs of eight species. [file 12864_2020_7083_MOESM4_ESM.docx]

**Additional file 4: Protein sequence of identified PYL members across 8 species**

The sequences of PYLs from Arabidopsis are available in the Arabidopsis Information Resource (<https://www.arabidopsis.org>), Brachypodium, Sorghum, barley, maize, foxtail millet and wheat genomes are available in the Ensembl plants <https://plants.ensembl.org/index.html>.

| Plant Name | Protein Name | Acession No./ Locus ID |
| --- | --- | --- |
| Arabidopsis | AtPYR1 | AT4G17870 |
| Arabidopsis | AtPYL1 | AT5G46790 |
| Arabidopsis | AtPYL2 | AT2G26040 |
| Arabidopsis | AtPYL3 | AT1G73000 |
| Arabidopsis | AtPYL4 | AT2G38310 |
| Arabidopsis | AtPYL5 | AT5G05440 |
| Arabidopsis | AtPYL6 | AT2G40330 |
| Arabidopsis | AtPYL7 | AT4G01026 |
| Arabidopsis | AtPYL8 | AT5G53160 |
| Arabidopsis | AtPYL9 | AT1G01360 |
| Arabidopsis | AtPYL10 | AT4G27920 |
| Arabidopsis | AtPYL11 | AT5G45860 |
| Arabidopsis | AtPYL12 | AT5G45870 |
| Arabidopsis | AtPYL13 | AT4g18620 |
| Rice | OsPYL1 | Os10g42280 |
| Rice | OsPYL2 | Os06g36670 |
| Rice | OsPYL3 | Os02g13330 |
| Rice | OsPYL4 | Os01g61210 |
| Rice | OsPYL5 | Os05g39580 |
| Rice | OsPYL6 | Os03g18600 |
| Rice | OsPYL7 | Os06g33480 |
| Rice | OsPYL8 | Os06g33640 |
| Rice | OsPYL9 | Os06g36690 |
| Rice | OsPYL10 | Os02g15640 |
| Rice | OsPYL11 | Os05g12260 |
| Rice | OsPYL12 | Os02g15620 |
| Rice | OsPYL13 | Os06g33490 |
| Brachypodium | BdPYL1 | Bradi3g34070 |
| Brachypodium | BdPYL2 | Bradi3g08580 |
| Brachypodium | BdPYL3 | Bradi1g37810 |
| Brachypodium | BdPYL4 | Bradi1g16710 |
| Brachypodium | BdPYL5 | Bradi1g65130 |
| Brachypodium | BdPYL6 | Bradi2g22510 |
| Brachypodium | BdPYL7 | Bradi2g53840 |
| Brachypodium | BdPYL8 | Bradi3g09580 |
| Brachypodium | BdPYL9 | Bradi2g32250 |
| Wheat | TaPYL1-A | TraesCS1A01G191700 |
| Wheat | TaPYL1-B | TraesCS1B01G206600 |
| Wheat | TaPYL1-D | TraesCS1D01G195300 |
| Wheat | TaPYL2-A | TraesCS3A01G154400 |
| Wheat | TaPYL2-D | TraesCS3D01G161500 |
| Wheat | TaPYL3-A | TraesCS7A01G358200 |
| Wheat | TaPYL3-B | TraesCS7B01G269600 |
| Wheat | TaPYL3-D | TraesCS7D01G364600 |
| Wheat | TaPYL4-A | TraesCS2A01G089400 |
| Wheat | TaPYL4-B | TraesCS2B01G105300 |
| Wheat | TaPYL4-D | TraesCS2D01G087500 |
| Wheat | TaPYL5-A | TraesCS3A01G348400 |
| Wheat | TaPYL5-B | TraesCS3B01G380300 |
| Wheat | TaPYL5-D | TraesCS3D01G342000 |
| Wheat | TaPYL6-A | TraesCS1A01G297600 |
| Wheat | TaPYL6-B | TraesCS1B01G306800 |
| Wheat | TaPYL6-D | TraesCS1D01G293600 |
| Wheat | TaPYL7-A | TraesCS4A01G114400 |
| Wheat | TaPYL7-B | TraesCS4B01G189800 |
| Wheat | TaPYL7-D | TraesCS4D01G191200 |
| Wheat | TaPYL8-A | TraesCS1A01G126800 |
| Wheat | TaPYL8-B | TraesCS1B01G145800 |
| Wheat | TaPYL8-D | TraesCS1D01G126900 |
| Wheat | TaPYL9-A | TraesCS7A01G350800 |
| Wheat | TaPYL9-B | TraesCS7B01G232100 |
| Wheat | TaPYL9-D | TraesCS7D01G328000 |
| Maize | ZmPYL1 | GRMZM2G134731 |
| Maize | ZmPYL2 | AC194914.3_FG002 |
| Maize | ZmPYL3 | GRMZM2G154987 |
| Maize | ZmPYL4 | GRMZM2G047677 |
| Maize | ZmPYL5 | GRMZM2G057959 |
| Maize | ZmPYL6 | GRMZM2G144224 |
| Maize | ZmPYL7 | GRMZM2G141382 |
| Maize | ZmPYL8 | GRMZM2G165567 |
| Maize | ZmPYL9 | GRMZM2G133631 |
| Maize | ZmPYL10 | GRMZM2G063882 |
| Maize | ZmPYL11 | GRMZM2G048733 |
| Maize | ZmPYL12 | GRMZM2G405064 |
| Maize | ZmPYL13 | GRMZM2G169695 |
| Barley | HvPYL1 | HORVU1Hr1G050110 |
| Barley | HvPYL2 | HORVU7Hr1G088140 |
| Barley | HvPYL3 | HORVU3Hr1G031380 |
| Barley | HvPYL4 | HORVU3Hr1G088100 |
| Barley | HvPYL5 | HORVU3Hr1G039010 |
| Barley | HvPYL6 | HORVU1Hr1G070420 |
| Barley | HvPYL7 | HORVU4Hr1G055220 |
| Barley | HvPYL8 | HORVU7Hr1G079270 |
| Barley | HvPYL9 | HORVU3Hr1G107030 |
| Sorghum | SbPYL1 | Sobic.001G289100 |
| Sorghum | SbPYL2 | Sobic.004G097800 |
| Sorghum | SbPYL3 | Sobic.010G169100 |
| Sorghum | SbPYL4 | Sobic.001G403300 |
| Sorghum | SbPYL5 | Sobic.009G170700 |
| Sorghum | SbPYL6 | Sobic.003G342000 |
| Sorghum | SbPYL7 | Sobic.009G080200 |
| Sorghum | SbPYL8 | Sobic.004G113800 |
| Foxtail millet | SiPYL1 | LOC101776342 |
| Foxtail millet | SiPYL2 | LOC101754623 |
| Foxtail millet | SiPYL3 | XP_004966082 |
| Foxtail millet | SiPYL4 | XP_004961771 |
| Foxtail millet | SiPYL5 | LOC101762326 |
| Foxtail millet | SiPYL6 | XP_012700774 |
| Foxtail millet | SiPYL7 | XP_004984885 |
| Foxtail millet | SiPYL8 | XP_004951286 |
| Foxtail millet | SiPYL9 | XP_004960624 |

Gate (SGLPA) and Latch (HRL) morifs are shaded in fluorescent green and magenta color, respectively.

**Arabidopsis ABA Receptors**

>AtPYR1

MPSELTPEERSELKNSIAEFHTYQLDPGSCSSLHAQRIHAPPELVWSIVRRFDKPQTYKHFIKSCSVEQNFEMRVGCTRDVIVISGLPANTSTERLDILDDERRVTGFSIIGGEHRLTNYKSVTTVHRFEKENRIWTVVLESYVVDMPEGNSEDDTRMFADTVVKLNLQKLATVAEAMARNSGDGSGSQVT

>AtPYL1

MANSESSSSPVNEEENSQRISTLHHQTMPSDLTQDEFTQLSQSIAEFHTYQLGNGRCSSLLAQRIHAPPETVWSVVRRFDRPQIYKHFIKSCNVSEDFEMRVGCTRDVNVISGLPANTSRERLDLLDDDRRVTGFSITGGEHRLRNYKSVTTVHRFEKEEEEERIWTVVLESYVVDVPEGNSEEDTRLFADTVIRLNLQKLASITEAMNRNNNNNNSSQVR

>AtPYL2

MSSSPAVKGLTDEEQKTLEPVIKTYHQFEPDPTTCTSLITQRIHAPASVVWPLIRRFDNPERYKHFVKRCRLISGDGDVGSVREVTVISGLPASTSTERLEFVDDDHRVLSFRVVGGEHRLKNYKSVTSVNEFLNQDSGKVYTVVLESYTVDIPEGNTEEDTKMFVDTVVKLNLQKLGVAATSAPMHDDE

>AtPYL3

MNLAPIHDPSSSSTTTTSSSTPYGLTKDEFSTLDSIIRTHHTFPRSPNTCTSLIAHRVDAPAHAIWRFVRDFANPNKYKHFIKSCTIRVNGNGIKEIKVGTIREVSVVSGLPASTSVEILEVLDEEKRILSFRVLGGEHRLNNYRSVTSVNEFVVLEKDKKKRVYSVVLESYIVDIPQGNTEEDTRMFVDTVVKSNLQNLAVISTASPT

>AtPYL4

MLAVHRPSSAVSDGDSVQIPMMIASFQKRFPSLSRDSTAARFHTHEVGPNQCCSAVIQEISAPISTVWSVVRRFDNPQAYKHFLKSCSVIGGDGDNVGSLRQVHVVSGLPAASSTERLDILDDERHVISFSVVGGDHRLSNYRSVTTLHPSPISGTVVVESYVVDVPPGNTKEETCDFVDVIVRCNLQSLAKIAENTAAESKKKMSL

>AtPYL5

MRSPVQLQHGSDATNGFHTLQPHDQTDGPIKRVCLTRGMHVPEHVAMHHTHDVGPDQCCSSVVQMIHAPPESVWALVRRFDNPKVYKNFIRQCRIVQGDGLHVGDLREVMVVSGLPAVSSTERLEILDEERHVISFSVVGGDHRLKNYRSVTTLHASDDEGTVVVESYIVDVPPGNTEEETLSFVDTIVRCNLQSLARSTNRQ

>AtPYL6

MPTSIQFQRSSTAAEAANATVRNYPHHHQKQVQKVSLTRGMADVPEHVELSHTHVVGPSQCFSVVVQDVEAPVSTVWSILSRFEHPQAYKHFVKSCHVVIGDGREVGSVREVRVVSGLPAAFSLERLEIMDDDRHVISFSVVGGDHRLMNYKSVTTVHESEEDSDGKKRTRVVESYVVDVPAGNDKEETCSFADTIVRCNLQSLAKLAENTSKFS

>AtPYL7

MEMIGGDDTDTEMYGALVTAQSLRLRHLHHCRENQCTSVLVKYIQAPVHLVWSLVRRFDQPQKYKPFISRCTVNGDPEIGCLREVNVKSGLPATTSTERLEQLDDEEHILGINIIGGDHRLKNYSSILTVHPEMIDGRSGTMVMESFVVDVPQGNTKDDTCYFVESLIKCNLKSLACVSERLAAQDITNSIATFCNASNGYREKNHTETNL

>AtPYL8

MEANGIENLTNPNQEREFIRRHHKHELVDNQCSSTLVKHINAPVHIVWSLVRRFDQPQKYKPFISRCVVKGNMEIGTVREVDVKSGLPATRSTERLELLDDNEHILSIRIVGGDHRLKNYSSIISLHPETIEGRIGTLVIESFVVDVPEGNTKDETCYFVEALIKCNLKSLADISERLAVQDTTESRV

>AtPYL9

MMDGVEGGTAMYGGLETVQYVRTHHQHLCRENQCTSALVKHIKAPLHLVWSLVRRFDQPQKYKPFVSRCTVIGDPEIGSLREVNVKSGLPATTSTERLELLDDEEHILGIKIIGGDHRLKNYSSILTVHPEIIEGRAGTMVIESFVVDVPQGNTKDETCYFVEALIRCNLKSLADVSERLASQDITQ

>AtPYL10

MNGDETKKVESEYIKKHHRHELVESQCSSTLVKHIKAPLHLVWSIVRRFDEPQKYKPFISRCVVQGKKLEVGSVREVDLKSGLPATKSTEVLEILDDNEHILGIRIVGGDHRLKNYSSTISLHSETIDGKTGTLAIESFVVDVPEGNTKEETCFFVEALIQCNLNSLADVTERLQAESMEKKI

>AtPYL11

METSQKYHTCGSTLVQTIDAPLSLVWSILRRFDNPQAYKQFVKTCNLSSGDGGEGSVREVTVVSGLPAEFSRERLDELDDESHVMMISIIGGDHRLVNYRSKTMAFVAADTEEKTVVVESYVVDVPEGNSEEETTSFADTIVGFNLKSLAKLSERVAHLKL

>AtPYL12

MKTSQEQHVCGSTVVQTINAPLPLVWSILRRFDNPKTFKHFVKTCKLRSGDGGEGSVREVTVVSDLPASFSLERLDELDDESHVMVISIIGGDHRLVNYQSKTTVFVAAEEEKTVVVESYVVDVPEGNTEEETTLFADTIVGCNLRSLAKLSEKMMELT

>AtPYL13

MESSKQKRCRSSVVETIEAPLPLVWSILRSFDKPQAYQRFVKSCTMRSGGGGGKGGEGKGSVRDVTLVSGFPADFSTERLEELDDESHVMVVSIIGGNHRLVNYKSKTKVVASPEDMAKKTVVVESYVVDVPEGTSEEDTIFFVDNIIRYNLTSLAKLTKKMMK

**Rice ABA Receptors**

>OsPYL1

MEQQEEVPPPPAGLGLTAEEYAQVRATVEAHHRYAVGPGQCSSLLAQRIHAPPAAVWAVVRRFDCPQVYKHFIRSCVLRPDPHHDDNGNDLRPGRLREVSVISGLPASTSTERLDLLDDAHRVFGFTITGGEHRLRNYRSVTTVSQLDEICTLVLESYIVDVPDGNTEDDTRLFADTVIRLNLQKLKSVSEANANAAAAAAAPPPPPPAAAE

>OsPYL2

MEAHVERALREGLTEEERAALEPAVMAHHTFPPSTTTATTAAATCTSLVTQRVAAPVRAVWPIVRSFGNPQRYKHFVRTCALAAGDGASVGSVREVTVVSGLPASTSTERLEMLDDDRHIISFRVVGGQHRLRNYRSVTSVTEFQPPAAGPAPAPPYCVVVESYVVDVPDGNTAEDTRMFTDTVVKLNLQKLAAVAEDSSSASRRRD

>OsPYL3

MEPHMERALREAVASEAERRELEGVVRAHHTFPAAERAAGPGRRPTCTSLVAQRVDAPLAAVWPIVRGFANPQRYKHFIKSCELAAGDGATVGSVREVAVVSGLPASTSTERLEILDDDRHVLSFRVVGGDHRLRNYRSVTSVTEFSSPSSPPSPPRPYCVVVESYVVDVPEGNTEEDTRMFTDTVVKLNLQKLAAVATSSSPPAAGNHH

>OsPYL4

MPYAAVRPSPPPQLSRPIGSGAGGGKACPAVPCEVARYHEHAVGAGQCCSTVVQAIAAPADAVWSVVRRFDRPQAYKKFIKSCRLVDGDGGEVGSVREVRVVSGLPATSSRERLEVLDDDRRVLSFRIVGGEHRLANYRSVTTVHEAAAPAMAVVVESYVVDVPPGNTWEETRVFVDTIVRCNLQSLARTVERLAPEAPRANGSIDHA

>OsPYL5

MMPYTAPRPSPPQHSRIGGCGGGGVLKAAGAAGHAASCVAVPAEVARHHEHAAGVGQCCSAVVQAIAAPVDAVWSVVRRFDRPQAYKHFIRSCRLLDGDGDGGAVAVGSVREVRVVSGLPATSSRERLEILDDERRVLSFRVVGGEHRLSNYRSVTTVHETAAGAAAAVVVESYVVDVPHGNTADETRMFVDTIVRCNLQSLARTAEQLALAAPRAA

>OsPYL6

MPCIPASSPGIPHQHQHQHHRALAGVGMAVGCAAEAAVAAAGVAGTRCGAHDGEVPMEVARHHEHAEPGSGRCCSAVVQHVAAPAPAVWSVVRRFDQPQAYKRFVRSCALLAGDGGVGTLREVRVVSGLPAASSRERLEILDDESHVLSFRVVGGEHRLKNYLSVTTVHPSPSAPTAATVVVESYVVDVPPGNTPEDTRVFVDTIVKCNLQSLANTAEKLAAGARAAGS

>OsPYL7

MNSGAGGAGGAAVGRMPAGSLQWAQWRLADERCELREEEMEYMRRFHRHEIGSNQCNSFIAKHVRAPLQNVWSLVRRFDQPQIYKPFVRKCVMRGNVETGSVREIIVQSGLPATRSIERLEFLDDNEYILRVKFIGGDHMLKKCGP

>OsPYL8

MNGAGGAGGAAAGKLPMVSHRQVQWRLADERCELREEEMEYIRQFHRHEPSSNQCTSFVAKHIKAPLQTVWSLVRRFDQPQLFKPFVRKCVMRENIIATGCVREVNVQSGLPATRSTERLELLDDNEHILKVKFIGGDHMLKNYSSILTIHSEVIDGQLGTLVVESFVVDIPEGNTKDDICYFIENILRCNLMTLADVSEERLANP

>OsPYL9

MNGVGGAGGAAAGKLPMVSHRRVQWRLADERCELREEEMEYIRRFHRHEPSSNQCTSFAAKHIKAPLHTVWSLVRRFDQPQLFKPFVRNCVMRENIIATGCIREVNVQSGLPATRSTERLELLDDNEHILKVKFIGGDHMLKNYSSILTIHSEVIDGQLGTLVVESFIVDVLEGNTKDDISYFIENVLRCNLRTLADVSEERLANP

>OsPYL10

MVEVGGGAAEAAAGRRWRLADERCDLRAAETEYVRRFHRHEPRDHQCSSAVAKHIKAPVHLVWSLVRRFDQPQLFKPFVSRCEMKGNIEIGSVREVNVKSGLPATRSTERLELLDDNEHILSVRFVGGDHRLKNYSSILTVHPEVIDGRPGTLVIESFVVDVPEGNTKDETCYFVEALLKCNLKSLAEVSERLVVKDQTEPLDR

>OsPYL11

MVGLVGGGGWRVGDDAAGGGGGGAVAAGAAAAAEAEHMRRLHSHAPGEHQCSSALVKHIKAPVHLVWSLVRSFDQPQRYKPFVSRCVVRGGDLEIGSVREVNVKTGLPATTSTERLELLDDDEHILSVKFVGGDHRLRNYSSIITVHPESIDGRPGTLVIESFVVDVPDGNTKDETCYFVEAVIKCNLTSLAEVSERLAVQSPTSPLEQ

>OsPYL12

MRGSTSLAVGCVREVDFKSGFPAKSSVERLEILDDKEHVFGVRIIGGDHRLKNYSSVLTAKPEVIDGEPATLVSESFVVDVPEGNTADETRHFVEFLIRCNLRSLAMVSQRLLLAQGDLAEPPAQ

>OsPYL13

MNGCTGGAGGVAAGRLPAVSLQQAQWKLVDERCELREEEMEYVRWFHRYELVATGATPSLPNTSGCPSKLGLPSTRRIERLGFPDDNDHTLRVKFIGGDHMLKDYSSTLIIHLEVIDGQLVTLVIESFVVDILEGNTKDEISYFIENLLKFNLRTLRV

**Wheat ABA Receptors**

>TaPYL1A
MEQQPVAAAEPEVPAGLGLTAAEYAQLLPTVEAYHRYAVGPGQCSSLVAQRIEAPPAAVWAIVRRFDCPQVYKHFIRSCALRPDPEAGDELRPGRLREVSVISGLPASTSTERLDLLDDARRAFGFTITGGEHRLRNYRSVTTVSELSPAAPAEICTVVLESYVVDVPDGNSEEDTRLFADTVVRLNLQKLKSVAEANAAAAAATPAPPAE

>TaPYL1B

MEQQPVAAAAAAEPEVPAGLGLTAAEYAQLLPTVEAYHRYAVGPGQCSSLVAQRIEAPPAAVWAIVRRFDCPQVYKHFIRSCALRPDPEAGDELRPGRLREVSVISGLPASTSTERLDLLDDARRAFGFTITGGEHRLRNYRSVTTVSELSPAAPAEICTVVLESYVVDVPDGNSEEDTRLFADTVVRLNLQKLKSVAEANAAAAATTAPPAE

>TaPYL1D

MEQQPVAAAATEPEVPAGLGLTAAEYAQLLPTVEAYHRYAVGPGQCSSLVAQRIEAPPAAVWAIVRRFDCPQVYKHFIRSCALRPDPEAGDELRPGRLREVSVISGLPASTSTERLDLLDDARRAFGFTITGGEHRLRNYRSVTTVSELSPAAPAEICTVVLESYVVDVPDGNSEEDTRLFADTVVRLNLQKLKSVAEANAAAAAATPAPPAE

>TaPYL2A

MESALRQGLTEPERREVEGVVEEHHTFPGRASGTCTSLVTQRVQAPLAAVWDIVRGFANPQRYKHFIKSCALAAGDGATVGSVREVTVVSGLPASTSTERLEILDDDRHILSFRVVGGEHRLRNYRSVTSVTEFTDQPSGPPYCVVVESYVVDVPEGNTEEDTRMFTDTVVKLNLQKLAAIATTTSSSSPPPSDEQS

>TaPYL2D

MESALRQGLTEPERREVEGVVEEHHTFPGRASGTCTSLVTQRVQAPLAAVWDIVRGFANPQRYKHFIKSCALAAGDGATVGSVREVTVVSGLPASTSTERLEILDDDRHILSFRVVGGEHRLRNYRSVTSVTEFADEPSGPSYCVVVESYVVDVPEGNTEEDTRMFTDTVVKLNLQKLAAIATTTTSSSPPPSDEQS

>TaPYL3A

MEAHMERALREGVTEAERAALEGTVRAHHTFPGRAPGGTCTSLVAQRVAAPVRAVWPIVRSFGNPQRYKHFVRTCALAAGDGASVGSVREVTVVSGLPASTSTERLEILDDDRHILSFSVVGGDHRLRNYRSVTSVTEFQPGPYCVVVESYVVDVPDGNTEEDTRMFTDTVVKLNLQKLASVAEDSAAAPGSRRRD

>TaPYL3B

MMEAHMERALQEGVTEAERAALEGTVRAHHTFPGRVPGATCTSLVAQRVAAPVRAVWPIVRSFGNPQRYKHFVRTCALAAGDGASVGSVREVTVVSGLPASTSTERLEILDDDRHILSFSVVGGEHRLRNYRSVTSVTEFQPGPYCVVVESYVVDVPDGNTEEDTRMFTDTVVKLNLQKLASVAEETAAAPGSRRRD

>TaPYL3D

MMEAHMEQALREGVTEAERAALEGTVRAHHTFPGRAPGATCTSLVAQRVAAPVRAVWPIVRSFGNPQRYKHFVRTCALAAGDGASVGSVREVTVVSGLPASTSTERLEILDDDRHILSFSVVGGEHRLRNYRSVTSVTEFQPGPYCVVVESYVVDVPEGNTEEDTRMFTDTVVKLNLQKLASVAEESAAAPGSRRRD

>TaPYL4A

MPRRLAATGSALLPGEPQVLRTIYIVRLAPPTFNLLPNQAKRSSAAARSAPIPPNKQLAGEATRSALEPMPTPYSAAALQQHHRLVSSSGGLATAAAAGAHRCGEHDGTVPPEVARHHEHAAPGGRCCCSAVVQRVAAPAADVWAVVRRFDQPQAYKSFVRSCALLDGDGGVGTLREVRVVSGLPAASSRERLEILDDERHVLSFSVVGGEHRLRNYRSVTTVHPAPGESASATLVVESYVVDVPPGNTPEDTRVFVDTIVKCNLQSLARTAEKLAGRGPAYGALP

>TaPYL4B

MPTPYSAAALQQHHRLVSSSGGLAVAAATGAHRCGEHDGTVPPEVARHHEHAAPGGRCCCSAVVQRVAAPAADVWAVVRRFDQPQAYKSFVRSCALLDGDGGVGTLREVRVVSGLPAASSRERLEILDDERHVLSFSVVGGEHRLRNYRSVTTVHPAPGGSASATLVVESYVVDVPPGNTPEDTRVFVDTIVKCNLQSLARTAEKLAGRGAAYGALP

>TaPYL4D
MPTPYSAAALQQHQRLVSSSGGLAATGAHRCGEHDGTVPPEVARHHEHAAPGGRCCCSAVVQRVAAPAADVWAVVRRFDQPQAYKSFVRSCALLDGDGGVGTLREVRVVSGLPAASSRERLEILDDERHVLSFSVVGGEHRLRNYRSVTTVHPAPGESASATLVVESYVVDVPPGNTPEDTRVFVDTIVKCNLQSLARTAEKLAGRGAAYGALP

>TaPYL5A
MPYAAARPSPQQHSRISSGCKALVAHGAAVPGEVARYHEHAAGAGQCCSAVVQAIAAPVEAVWSVVRRFDRPQAYKRFIKSCRMVDGDGGAVGSVREVRVVSGLPGTSSRERLEILDDERRVLSFRIVGGEHRLANYRSVTTVSEVASTVAGAPRVTLVVESYVVDVPPGNTSDETRLFVDTIVRCNLQSLARTAEQLALAVPHVN

>TaPYL5B
MPYAAARPSLQQHSRISSGCKALVAHGAAVPGEVALYHEHAAGAGQCCSAVVQAIAAPVEAVWSVVRRFDRPQAYKRFIKSCRVVDGDGGAVGSVREVRVVSGLPGTSSRERLEILDDERRVLSFRIVGGEHRLANYRSVTTVNEVASTVAAGAPRVTLVVESYVVDVPPGNTSDETRLFVDTIVRCNLQSLARTAEQLALAVPHVN

>TaPYL5D

MPYAAARPSPQQHSRISAGCKALVAHGAAVPGEVARYHEHAAGAGQCCSAVVQAIAAPVEAVWSVVRRFDRPQAYKRFIKSCRLVDGDGGAVGSVREVRVVSGLPGTSSRERLEILDDERRVLSFRIVGGEHRLANYRSVTTVSEVASTVAGAPRVTLVVESYVVDVPPGNTSDETRMFVDTIVRCNLQSLARTAEQLALAVPHVN

>TaPYL6A
MPYTASRPSAPQRARVAAGGGGWKAAAHAASCGAVPGEVARHHEHAAGAGQCCSAVVQAIEAPVGAVWAVVRRFDRPQAYKHFIRSCRVVDGDGGAVGSVREVRVVSGLPATSSRERLEILDDERRVLSFRVVGGEHRLSNYRSVTTVHEAASAGAVVVESYVVDVPPGNTADETRTFVDTIVRCNLQSLARTAQQLALAA

>TaPYL6B
MPYTASRPSAPQRARVAAVGAGWKAAAHAASCGAVPGEVARHHEHAAGTGQCCSAVVQAIEAPVGAVWAVVRRFDRPQAYKHFIRSCRLVDGDGGAVGSVREVRVVSGLPATSSRERLEILDDERRVLSFRVVGGEHRLSNYRSVTTVHEAASAGAVVVESYVVDVPPGNTADETRTFVDTIVRCNLQSLARTA

>TaPYL6D
MPYTASRPSAPQRARVAAVGAGWKAAAHAASCGAVPGEVARHHEHAAGAGQCCSAVVQAIEAPVGAVWAVVRRFDRPQAYKHFIRSCRVVDGDGGAVGSVREVRVVSGLPATSSRERLEILDDERRVLSFRVVGGEHRLSNYRSVTTVHEAAPAGAVVVESYVVDVPPGNTADETRTFVDTIVRCNLQSLARTAQQLAVPA

>TaPYL7A
MRCREHDCEVPAEVARHHEHAEPGSGQCCSAVVQHVAAPAAAVWSVVRRFDQPQAYKRFVRSCALVAGDGGVGTLREVHVVSGLPAASSRERLEILDDESHVLSFRVVGGEHRLKNYLSVTTVHPSPAAPSSATVVVESYVVDVPAGNTTEDTRVFIDTIVKCNLQSLAKTAEKVAAVS

>TaPYL7B

MPCIPVSSPSIQHHNHNHHHRVLAGVGVGMGCGAEAVVAAAGTAGMRCREHDCEVPAEVARHHEHAEPGSGQCCSAVVQHVAAPAAAVWSVVRRFDQPQAYKRFVRSCALVAGDGGVGTLREVHVVSGLPAASSRERLEILDDESHVLSFRVVGGEHRLKNYLSVTTVHPSPAAPSSATVVVESYVVDVPAGNTIEDTRVFIDTIVKCNLQSLAKTAEKLAAVS

>TaPYL7D
MPCIPASSPSIQHHNHNHHHRVLAGVGVGMGCGAEAVVAAAGTAGMRCREHDCEVPAEVARHHEHAEPGSGQCCSAVVQHVAAPAAAVWSVVRRFDQPQAYKRFVRSCALVAGDGGVGTLREVHVVSGLPAASSRERLEILDDESHVLSFRVVGGEHRLKNYLSVTTVHPSPAAPSSATVVVESYVVDVPAGNTIEDTRVFIDTIVKCNLQSLAKTAEKVAAVS

>TaPYL8A

MVGLVGGGARAWRLSDEAANGAGGGGAATEADYMRRLHGHAPGENQCTSALVKHIKAPVHLVWSLVRSFDQPQRYKPFVSRCVVRGGDLEIGSVREVNVKTGLPATTSTERLEQLDDDEHILSVKFVGGDHRLRNYSSIITVHPQSIDGRPGTLVIESFVVDVPDGNTKDETCYFVEAVIKCNLTSLAEVSERLAVQSPTSPLEQ

>TaPYL8B

MVGLLGGGARAWRLSDEAANGAVGGGAATEADYMRRLHGHAPGENQCTSALVKHIKAPVHLVWSLVRSFDQPQRYKPFVSRCVVRGGDLEIGSVREVNVKTGLPATTSTERLEQLDDDEHILSVKFVGGDHRLRNYSSIITVHPQSIDGRPGTLVIESFVVDVPDGNTKDETCYFVEAVIKCNLTSLAEVSERLAVQSPTSPLEQ

>TaPYL8D

MVGLVGGGARAWRLSDEAANGAGGGGVATEADYMRRLHGHAPGENQCTSALVKHIKAPVHLVWSLVRSFDQPQRYKPFVSRCVVRGGDLEIGSVREVNVKTGLPATTSTERLEQLDDDEHILSVKFVGGDHRLRNYSSIITVHPQSIDGRPGTLVIESFVVDVPDGNTKDETCYFVEAVIKCNLTSLAEVSERLAVQSPTSPLEQ

>TaPYL9A

MDGGSSGVGADGIWRPWDEHTVLRPEEMEYVRRFHQHVPGANQCTSFIAKHIKAPLQTVWSVVRRFDKPQVYKRFVENCVMQGNIEPGCVREVTLKSGLPGKWSIERLELLDDNEHILSVMFIDGDHPLKNYSSILTVHHEVADGHPGALVIESFVVDIPKENTENEIFYLVGNFLKFNHKLLADVSEGQIDRRALN

>TaPYL9B

MDGGSSGVGADEIWRPWDEHTVLRPEEMEYVRQFHQHEPGANQCTSFIAKHIKAPLQTVWSLVRRFDEPQVFKPFVEKCVMQGNIEPGCVREVTIKSGLPGTWSTERLELLDDNEHILSVKFIDGDHPLKNYSSILTVHHEVIGGHPGALVIESFVVDIPEENTENEIFYLVGNFIKINHNLLADVSERRNRALN

>TaPYL9D

MDGGSSGVGADGIWRPWDEHTVLRPEEMEYVRRFHQHEPGANQCTSFIAKHIKAPLQTVWSVVRRFDKPQVYKRFVENCVMQGNIEPGCVREVTLKSGLPGKWSIERLELLDDNEHILSVKFIDGDHPLKNYSSILTVHHEVIDGHPGALVIESFVVDIPEENTKNEIFYLVGNFLKFNHKLLADVSEGRIDRRALN

**Maize ABA Receptors**

>ZmPYL1

MDQQGAGGDVEVPAGLGLTAAEYEQLRPTVDAHHRYAVGEGQCSSLLAQRIHAPPAAVWAIVRRFDCPQVYKHFIRSCAVRPDPDAGDALRPGRLREVCVISGLPASTSTERLDHLDDAARVFGFSITGGEHRLRNYRSVTTVSELAGPGICTVVLESYAVDVPDGNTEDDTRLFADTVIRLNLQKLKSVAEASTSSSAPPPPSE

>ZmPYL2

MDQQGAGGDAEVPAGLGLTAAEYEQLRSTVDAHHRYAVGEGQCSSLLAQRIHAPPEAVWAVVRRFDCPQVYKHFIRSCALRPDPEAGDALCPGRLREVSVISGLPASTSTERLDLLDDAARVFGFSITGGEHRLRNYRSVTTVSELADPAICTVVLESYVVDVPDGNTEDDTRLFADTVIRLNLQKLKSVAEANAAEAAATTNSVLLPRPAE

>ZmPYL3

MEPHMESALRQGLSEAEQRELEGVVRAHHTFPGRAPGTCTSLVTQRVDAPLAAVWPIVRGFGSPQRYKHFIKSCDLKAGDGATVGSVREVTVVSGLPASTSTERLEILDDHRHILSFRVVGGDHRLRNYRSVTSVTEFQPGPYCVVLESYVVDVPDGNTEEDTRMFTDTVVKLNLQKLAAIATSSSAN

>ZmPYL4

MPYTAPRPSPQQHSRVLSGGGAKAASHGASCAAVPAEVARHHEHAARAGQCCSAVVQAIAAPVGAVWSVVRRFDRPQAYKHFIRSCRLVGGGDVAVGSVREVRVVSGLPATSSRERLEILDDERRVLSFRVVGGEHRLANYRSVTTVHEAGAGAGTGTVVVESYVVDVPHGNTADETRVFVDTIVRCNLQSLARTAERLA

>ZmPYL5

MPCLQASSPGSMPYQHHGRGVGCAAEAGAAVGASAGTGTRCGAHDGEVPAEAARHHEHAAPGPGRCCSAVVQRVAAPAEAVWSVVRRFDQPQAYKRFVRSCALLAGDGGVGTLREVRVVSGLPAASSRERLEVLDDESHVLSFRVVGGEHRLQNYLSVTTVHPSPAAPDAATVVVESYVVDVPPGNTPEDTRVFVDTIVKCNLQSLATTAEKLALAAV

>ZmPYL6

MPCIQASSPGGMPHQHGRGRVLGGGVGCAAEVAAAVAASAGGMRCGAHDGEVPAEAARHHEHAAAGPGRCCSAVVQHVAAPAAAVWSVVRRFDQPQVYKRFVRSCALLAGDGGVGTLREVRVVSGLPAASSRERLEVLDDESHVLSFRVVGGEHRLRNYLSVTTVHPSPAAPDAATVVVESYVVDVPPGNTPEDTRVFVDTIVKCNLQSLATTAEKLAAV

>ZmPYL7

MLLYMCSTSPPNPISTSPLQRETTRSIDQEDRRGSSSRPTMPYAATRTSPQQHSRVASNGRAVAACAGHAGVPDEVARHHEHAVAAGQCCSVMVQSIAAPADAVWSLVRRFDQPQGYKRFIRSCHLVDGDGVEVGSVRELLVVSGLPAENSRERLEIRDDERRVISFRILGGDHRLANYRSVTTVHEAASEGGPLTMVVESYVVDVPPGNTVEETRIFVDTIVRCNLQSLEDTVIRQQAMAAPAAPHNDHNHS

>ZmPYL8

MVGLVGGSTARAEHVVANAGGEAEYVRRMHRHAPTEHQCTSTLVKHIKAPVHLVWELVRRFDQPQRYKPFVRNCVVRGDQLEVGSLRDVNVKTGLPATTSTERLEQLDDDLHILGVKFVGGDHRLQVRAAAARLLRPCKNALFHAPSLLVSVLQIIFGGMLALLPFLFFLN

>ZmPYL9

MVGLVGGSTARAEHVVANAGGETEYVRRLHRHAPAEHQCTSTLVKHIKAPVHLVWELVRSFDQPQRYKPFVRNCVVRGDQLEVGSLRDVNVKTGLPATTSTERLEQLDDDLHILGVKFVGGDHRLQNYSSIITVHPESIDGRPGTLVIESFVVDVPDGNTKDETCYFVEAVIKCNLKSLAEVSEQLAVESPTSPIDQ

>ZmPYL10

MVMVEMDGGVGGGGGGGQTPAPRRWRLADERCDLRAMETDYVRRFHRHEPREHQCSSAVAKHIKAPVHLVWSLVRRFDQPQLFKPFVSRCEMKGNIEIGSVREVNVKSGLPATRSTERLELLDDNEHILSVRFVGGDHRLQNYSSILTVHPEVIDGRPGTLVIESFVVDVPDGNTKDETCYFVEALLKCNLKSLAEVSERQVVKDQTEPLDR

>ZmPYL11

MVVEMDGGVGVAAAGGGGAQTPAPPPPRRWRLADERCDLRAMETDYVRRFHRHEPRDHQCSSAVAKHIKAPVHLVWSLVRRFDQPQLFKPFVSRCEMKGNIEIGSVREVNVKSGLPATRSTERLELLDDDERILSVRFVGGDHRLQVCSVLHLSIFCAAHARYFAHHLKCVLEFLCQMHLDVLPCDDAILE

>ZmPYL12

MVGLVGGSTARAEHVVANAGGEAEYVRRMHRHAPTEHQCTSTLVKHIKAPVHLVWELVRRFDQPQRYKPFVRNCVVRGDQLEVGSLRDVNVNPGLPATTSTERLEQLDDDLHILGVKFVGGDHRLQLAGVVAVEVTGGPDVPFHPGREFKQIVMNMLDCFRLTVEYGVRSSQNLGDEQPQACEIKAQLVRTAEAARQLALMLEVERPSYQGRNSVYQSSSKMMSAISVAHLGCKDMDAVDVGVVGMVDSQSLSSALEKLYFWERKLYAEVK

>ZmPYL13

MSIPTSHIPIHIHPPTPTRPPTVFLVFTFLTQFTHPLTISPIDHCCHIPNPLPVVFGVFPAAAAAVAVVHHTVPLAVISMRERNSSIDQEHQRGSSSRSTMPFAASRTSQQQHSRVATNGRAVAVCAGHAGVPDEVARHHEHAVAAGQCCAAMVQSIAAPVDAVWSLVRRFDQPQRYKRFIRSCHLVDGDGAEVGSVRELLLVSGLPAESSRERLEIRDDERRVISFRVLGGDHRLANYRSVTTVHEAAPSQDGRPLTMVVESYVVDVPPGNTVEETRIFVDTIVRCNLQSLEGTVIRQLEIAAMPHDDNQN

**Barley ABA Receptors**

>HvPYL1

MEQQPVAAAPAAEPEVPAGLGLTAAEYAQLLPTVEAYHRYAVGPGQCSSLVAQRIEAPPAAVWAIVRRFDCPQVYKHFIRSCALRPDPEAGDDLRPGRLREVSVISGLPASTSTERLDLLDDARRAFGFTITGGEHRLRNYRSVTTVSELSPAAPAEICTVVLESYVVDVPDGNSEEDTRLFADTVVRLNLQKLKSVAEANAAAAAAAPPAE

>HvPYL2

MEAHMERALREGVTEAERAALEGTVRAHHTFPGRAPGATCTSLVAQRVAAPVRAVWPIVRSFGNPQRYKHFVRTCALAAGDGASVGSVREVTVVSGLPASTSTERLEILDDDRHILSFSVVGGEHRLRNYRSVTSVTEFQPGPYCVVVESYVVDVPDGNTEEDTRMFTDTVVKLNLQKLASVAEESGAAPGSRRRD

>HvPYL3

PQRTQVGLLLVNCTLARERKERERKGESMEHHMESALRQGLTEPERRELEGVVEEHHTFPGRASGTCTSLVTQRVQAPLAAVWDIVRGFANPQRYKHFIKSCALAAGDGATVGSVREVTVVSGLPASTSTERLEILDDDRHILSFCVVGGEHRLRNYRSVTSVTEFTDQPSGPSYCVVVESYVVDVPEGNTEEDTRMFTDTVVKLNLQKLAAIATTTSSPPPLDGQS

>HvPYL4

MPYAAARPSPQQHSRISAACKALVAQGAAVPGEVARHHEHAAGAGQCCSAVVQAIAAPVEAVWSVVRRFDRPQAYKRFIKSCRLVDGDGGAVGSVREVRVVSGLPGTSSRERLEILDDERRVLSFRIVGGEHRLANYRSVTTVNEVASTVAGAPRVTLVVESYVVDVPPGNTGDETRMFVDTIVRCNLQSLARTAEQLALAAPRVN

>HvPYL5

MPYAAARPSPQQHSRISAACKALVAQGAAVPGEVARHHEHAAGAGQCCSAVVQAIAAPVEAVWSVVRRFDRPQAYKRFIKSCRLVDGDGGAVGSVREVRVVSGLPGTSSRERLEILDDERRVLSFRIVGGEHRLANYRSVTTVNEVASTVAGAPRVTLVVESYVVDVPPGNTGDETRMFVDTIVRCNLQSLARTAEQLALAAPRVN

>HvPYL6

MPYTASRPSAPQRARVAAGGGWKAAAHAASCGAVPGEVARHHEHAAGAGQCCSAVVQAIEAPVGAVWAVVRRFDRPQAYKHFIRSCRLVDGDGGAVGSVREVRVVSGLPATTSRERLEILDDERRVLSFRVVGGEHRLSNYRSVTTVHETASAGGAVVVESYVVDVPPGNTDDETRTFVDTIVRCNLQSLARTAQQLALAA

>HvPYL7

MPCIPASSPSIQHHNHHHRVLAGVGVGVGMGCGAEAVVAAAGTAGMRCGEHDCEVPAEVARHHEHAEPGSGQCCSAVVQHVAAPAAAVWSVVRRFDQPQAYKRFVRSCALVAGDGGVGTLREVHVVSGLPAASSRERLEILDDESHVLSFRVVGGEHRLKNYLSVTTVHPSPAAPSSATVVVESYVVDVPAGNTIDDTRVFIDTIVKCNLQSLAKTAEKLAAVS

>HvPYL8

MDGGSSGVGADGIWRPWDEHTMLCLKEMEYVRRFHQHELGANQCTSFIAKHIKAPLQTVWSVVRRFDKPQVFKPFVEKCVMQGNIEPGCVREVTVKSGLPAKWSIERLELLDDNEHILRVKFIDGNHPLKNYSSILTVHHEVIDGHPGALVIESFVVDVPEENTENEIFYLVGNFLKVNHKLLADVSEGRIDGRALN

>HvPYL9

MRSFSNPQRYKHFVRTSALVAGDEASVGNAREDTVVSGLPAFTSSERLEILDNGRHILSFSIVDGEYCLRNYRSVSSITEFQSGPYCAVVKSYVVPDGWRHSAQQGKNLSGGFSQQDGAALIFLHFIFIPQIQLCSHPNQCGNVFNIWELDHKYYSKTGSTPKLGDFSVFSFLLQFSSYLLPLVSASKLFGQIFYSLLMYPHSISDAALKYDSLRCPLPLPVTMSLLLLQKMAKRSSVQAKHTISEYGKAGDRKNITSNAPSGPLLCFLYSWLHSTQDLFSKHVSCLDL

**Sorghum ABA Receptors**

>SbPYL1

MVESPNPNSPSRPLCIKYTRAPARHFSPPLPFSSLIISANPIEPKAMDKQGAGGDVEVPAGLGLTAAEYEQLRSTVDAHHRYAVGEGQCSSLLAQRIQAPPAAVWAIVRRFDCPQVYKHFIRSCALRPDPEAGDALRPGRLREVSVISGLPASTSTERLDLLDDAARVFGFSITGGEHRLRNYRSVTTVSELADPGICTVVLESYVVDVPDGNTEDDTRLFADTVIRLNLQKLKSVAEANAAAAASFVSVVPPPEPEE

>SbPYL2

MEPHMETALRQGGLSELEQRELEPVVRAHHTFPGRSPGTTCTSLVTQRVDAPLSAVWPIVRGFAAPQRYKHFIKSCDLRSGDGATVGSVREVTVVSGLPASTSTERLEILDDDRHILSFRVVGGDHRLRNYRSVTSVTEFHHHHQAAAGRPYCVVVESYVVDVPEGNTEEDTRMFTDTVVKLNLQKLAAIATSSAAAAASNSST

>SbPYL3

METHVERALRATLTEAEVRALEPAVREHHTFPAGRVAAGTTTPTPTTCTSLVAQRVSAPVRAVWPIVRSFGNPQRYKHFVRTCALAAGDGASVGSVREVTVVSGLPASSSTERLEVLDDDRHILSFRVVGGDHRLRNYRSVTSVTEFQPGPYCVVVESYAVDVPEGNTAEDTRMFTDTVVRLNLQKLAAVAEESAAAAAAGNRR

>SbPYL4

MPCLQASSSPGSMPHQHHGRVLAGVGCAAEVAAAAVAATSPAAGMRCGAHDGEVPAEAARHHEHAAPGPGRCCSAVVQHVAAPASAVWSVVRRFDQPQAYKRFVRSCALLAGDGGVGTLREVRVVSGLPAASSRERLEVLDDESHVLSFRVVGGEHRLQNYLSVTTVHPSPAAPDAATVVVESYVVDVPPGNTPEDTRVFVDTIVKCNLQSLATTAEKLAAV

>SbPYL5

MPYTAPRPSPQQHSRVTGGGAKAAIVAASHGASCAAVPAEVARHHEHAARAGQCCSAVVQAIAAPVGAVWSVVRRFDRPQAYKHFIRSCRLVDDGGGGAGAGAGATVAVGSVREVRVVSGLPATSSRERLEILDDERRVLSFRVVGGEHRLANYRSVTTVHEAEAGAGGTVVVESYVVDVPPGNTADETRVFVDTIVRCNLQSLARTAERLALALA

>SbPYL6

MPYAAATRTSPEQHSRVVTNGRAAVACAGHAGVPAEVARHHEHTVAAGQCCSVMMRSIAAPVDAVWSLVRRFDQPQGYKGFIRSCHLVDGDGIEVGSVRELEVVTGLPAQNSRERLEIRDDERRVIGFRILGGDHRLANYRSVTTVHEAASQNGGGPLTMVVESYVVDVPQGNTVEETHIFVDTIVRCNLQSLERTVLRQQAMAAAPHNNHNHN

>SbPYL7

MVGLVGGSTARAEHVVANAGGETEYVRRLHRHAPAEHQCTSTLVKHIKAPVHLVWELVRSFDQPQRYKPFVRNCVVRGDQLEVGSVRDVNVKTGLPATTSTERLEQLDDDLHILGVKFVGGDHRLQNYSSIITVHPESIDGRPGTLVIESFVVDVPDGNTKDETCYFVEAVIKCNLKSLAEVSEQLAVEPPTSPIDQ

>SbPYL8

MVEMDGGVGVVGGGQQTPAPRRWRLADELRCDLRAMETDYVRRFHRHEPRDHQCSSAVAKHIKAPVHLVWSLVRRFDQPQLFKPFVSRCEMKGNIEIGSVREVNVKSGLPATRSTERLELLDDNEHILSVKFVGGDHRLQNYSSILTVHPEVIDGRPGTLVIESFVVDVPDGNTKDETCYFVEALLKCNLKSLAEVSERQVIKDQTEPLDR

**Brachypodium ABA Receptors**

>BdPYL1
MEPQQQPDAAAAAGAGAGEPEVPAGLGLTAAEYAQLRPTVEAYHLYAVGQGQCSSLLAQRIEAPAAAVWAIVRRFDCPQVYKHFIRNCALRPDPNAGAGEDDGELRPGRLREVSVISGLPASTSTERLDLLDDARRAFGFTIIGGEHRLRNYRSVTTVSEIRAAGAAAVVLESYIVDVPEGNSEEDTRLFADTVVRLNLQKLKSVAEANAASNAPAPPPAE

>BdPYL2
MEPHMERALREALTEAERRSLAPVVAAHHTFPGSGQSSPSPSPGKKKTCTSLVTQRVDAPLAAVWAIVRGFATPQRYKHFIKSCALAAGDGATVGSVREVTVVSGLPASTSTERLEILDDDRHVLSFRVVGGEHRLRNYRSVTSVTEFSSPPPPEPEEKEKEETQDAAASSSYCVVVESYVVDVPEGNTEEDTRMFTDTVVKLNLQKLAAIATTSTSSPSASPSSPPPPQPSDGAQADH

>BdPYL3
MEAHMERALREGLTEAERASLEGAVRAHHTFPGRAATCTSLVAQRVAAPVRDVWPIVRSFGNPQRYKHFVRTCALAAGDGASVGSVREVTVVSGLPASTSTERLEILDDDRHILSFSVVGGEHRLRNYRSVTSVTEFQGQEDAGAPPYCVVLESYVVDVPPGNTEDDTRMFTDTVVKLNLQKLASVAEESGSRTRD

>BdPYL4
MPAPYSAAAAQQLQQHRPLAAAVTGSRCGEHDGTVPAEVAQHHSHPPSSSAGPWRCCSAVVQRVRAPTSAVWSVVRRFGEPQAYKSFVRSCAVVDGDGGVGTLREVRVVSGLPAASSRERLEVLDDDRRVLSFRVVGGEHRLRNYRSVTTVHPSSSSSSPAEEETVVVESYVVEVPAGNTAEDTRTFVDTIVKCNLLSLARTAEKLSAAGRCP

>BdPYL5
MPCIPASSPSSIHHHHHQRHRVLAMGCGAELAAVQGASGMARCGAHDGEVPAEVSRHHEHAAADPAGSGMRCCSAVVQHVAAPAADVWSVVRRFDQPQAYKRFVRSCALVAGDGGVGTLREVRVVSGLPAASSRERLEVLDDESHVLSFRVVGGEHRLKDYLSVTTVHPSPAAPSSATVVVESYVVDVPPGNTVEDTRVFIDTIVKCNLQSLAKTAEKLAAGGRAVS

>BdPYL6
MPYTASRSRPSPAQRSRVGGGRKGAAAAVPEEVARHHEHAAGAGQCCSAVVQESIAAPVEAVWAVVRRFDRPQAYKHFIRSCRLVDGDGGAVGSVREVRVVSGLPATSSRERLEILDDERRVLSFRVVGGEHRLSNYRSVTTVHHAETTGSTVVVESYVVDVPAGNTADETRTFVDTIVRCNLQSLARTAEQLAAAD

>BdPYL7
MPYTATRPSPPQHSRTVGAARNKAPLAVPLPAEVARYHEHAAGAGQCGSAVVQAIGAPAEAVWAVVRRFDRPQAYKRFVKSCRLVEDGGSVGVGSVREVRVVSGLPATCSRERLEVLDDERRVLSFRIVGGEHRLANYRSVTTVSEVPVAGGAGKPVSVVVESYVVDVPPGNTGDETRVFVDTIVRCNLLSLARAAEAEAQLALAPVQSPRVS

>BdPYL8
MVEIDGAVGVGGGAGGVEGARRWRLADERCDLRATESDYVRRFHPHEPRDHQCSSAVAKHIKAPVHLVWSLVRRFDQPQLFKPFVSRCEMKGNIEIGSVREVNVKSGLPATRSTERLELLDDTEHILSVKFVGGDHRLKNYSSILTVHPEVIDGRPGTLVIESFVVDVPEGNTKDETCYFVEALIKCNLKSLAEVSERLVVKDQTEPLDR

>BdPYL9
MVGLVDGSARGWRLSDEAATSGAGRGGGGGGVTAAADHMRRLHSHALGEHQCSSTLLKHIKAPVHLVWSLVRSFDQPQRYKPFVSRCVVRGGDLEIGSLREVNVKTGLPATTSTERLEQLDDEEHILSVKFVGGDHRLRNYSSIITVHPESIDGRPGTLVIESFVVDVPDGNTKDETCYFVEAVIKCNLTSLAEVSERLAVQSPTSPLEQ

**Foxtail Millet ABA Receptors**

>SiPYL1

MDQQGAGADGEVPAGLGLTAAEYEQLRSTVEAHHRYAVGAGQCSSLLAQRIHAPPAAVWAIVRRFDCPQVYKHFIRSCALRPDPEAGDNLRPGRLREVSVISGLPASTSTERLDLLDDAARVFGFSITGGEHRLRNYRSVTTVNELAGPGICTVVLESYVVDVPDGNTEDDTRLFADTVIRLNLQKLKSVAEANAAAAAAPPPEPAE

>SiPYL2

MEPHMEGALRQGLSEAEQRELESVVRAHHTFPGRAPGTCTSLVTQRVDAPLAAVWPIVRGFASPQRYKHFIKSCDLRSGDGATVGSVREVTVVSGLPASTSTERLEILDDDRHILSFRVVGGDHRLRNYRSVTSVTEFQPGPYCVVVESYVVDVPEGNTEEDTRMFTDTVVKLNLQKLAAIATSSSSSPRPSGGGGGGTDH

>SiPYL3

MEAHVERALRATLTEAEARALEGTVHEHHTFPGRSRAGAGAAAAAATCTSLVAQRVSAPVRCVWPIVRSFGNPQRYKHFVRTCALAAGDGASVGSVREVTVVSGLPASSSTERLEVLDDDRHILSFRVVGGDHRLRNYRSVTSVTEFQRQHPAGGPPYCVVVESYVVDVPEGNTEEDTRMFTDTVVRLNLQRLAAVAEESAGGGRS

>SiPYL4

MPYTAPRPSPQQHSRITGCGGGGVGKAASHGPSCAAVPGEVARHHEHAARAGQCCSAVVQAIAAPVGAVWSVVRRFDRPQAYKHFIRSCRLVDGDGGAVGSVREVRVVSGLPATSSRERLEILDDERRVLSFRVVGGEHRLANYRSVTTVHEAAAGHTVVVESYVVDVPPGNTADETRTFVDTIVRCNLQSLARTAEQLAAALA

>SiPYL5

MPYATTRTSPQQHSRITTNGRVVSMCPGHTEVPGEVARHHEHVVAAGQCCSVMVQTIAAPVDAVWSLVRRFDQPQEYKSFIRSCRLVDGDGTTVGSERELIVQSGLPANSSRDRLEILDDALRVISFKILGGEHRLSNYRSVTTVHEATSSDGPVAMVVESYVVDVPPGNTAQETCVFTNTIIRANLQNLERKVMGRLAMAAPHQNH

>SiPYL6

MRKRKDNVCAFDLDTTQRDPPSSSTSSQPCAPGQCCSAVVQAIAVPVGAVWLVVRRFDRPQAYKHFIRSCRLVDGDGGAMGSVREVRVVSGLPATSIHEQLEILDDEHRVLSFRVVGGEHRLANYRSVTTVHEAAAGHTVVVKSYVVDVPPGNTXDETRTFVDTIVRGNLQSLARTAEQLAAALA

>SiPYL7

MPCIQASSPGSMPHQHHGRVLAGVGCAAQVAAAVAASTVGMRCGAHDGEVPAEAARHHEHGAPGPGRCCSAVVQHVAAPAAAVWSVVRRFDQPQAYKRFVRSCALLAGDGGVGTLREVRVVSGLPAASSRERLEILDDESHVLSFRVVGGEHRLQNYLSVTTVHPSPAAPDAATVVVESYMVDVPPGNTPEDTRVFVDTIVRCNLQSLATTAEKLAAVST

>SiPYL8

MVEMDGAVGVGGGGQAPAPAPRLWRLVDERCDLRAMESEYVRRFHRHEPRDHQCSSAVAKHIKAPVHLVWSLVRRFDQPQLFKPFVSRCEMKGNIEIGSVREVNVKSGLPATRSTERLELLDDNEHILSVKFVGGDHRLQNYSSILTVHPEVIDGRPGTLVIESFVVDVPDGNTKDETCYFVEALLKCNLKSLAEVSENRVTGDQTEPLDR

>SiPYL9

MVGLVGGGAARGAARLGAGDPAAVANGGGEADHVRRLHRHAPADHQCTSTLVKHIKAPVHLVWELVRSFDQPQRYKPFVSRCVVRGDQLEIGSLREVNVKTGLPATTSTERLEQLDDDEHILGVKFVGGDHRLQNYSSIITVHPESIDGRPGTLVIESFVVDVPDGNTKDETCYFVEAVIKCNLTSLAEVSERLAVQSPTSPLEH
