# Supplementary material for: Genome-wide identification and characterization of ABA receptor PYL gene family in rice
Source: BMC Genomics. 2020 Sep 30;21:676. doi: 10.1186/s12864-020-07083-y (PMC7526420; doi:10.1186/s12864-020-07083-y)
Supplement: Supplementary file 5 — Additional file 5 : Table S2. Pair wise collinearity among PYLs of Arabidopsis and rice. [file 12864_2020_7083_MOESM5_ESM.docx]

**Additional file 5: Table S2.** Table depicting pair wise collinearity between PYL genes

| AtPYR1 (AT4G17870) | AtPYL1 (AT5G46790) |
| --- | --- |
| AtPYL1 (AT5G46790) | OsPYL1 (Os10g42280) |
| AtPYL4 (AT2G38310) | AtPYL5 (AT5G05440) |
| AtPYL4 (AT2G38310) | OsPYL4 (Os01g61210) |
| AtPYL4 (AT2G38310) | OsPYL5 (Os05g39580) |
| AtPYL6 (AT2G40330) | AtPYL5 (AT5G05440) |
| AtPYL5 (AT5G05440) | OsPYL4 (Os01g61210) |
| AtPYL5 (AT5G05440) | OsPYL6 (Os03g18600) |
| AtPYL5 (AT5G05440) | OsPYL5 (Os05g39580) |
| AtPYL6 (AT2G40330) | OsPYL4 (Os01g61210) |
| AtPYL6 (AT2G40330) | OsPYL6 (Os03g18600) |
| AtPYL6 (AT2G40330) | OsPYL5 (Os05g39580) |
| AtPYL9 (AT1G01360) | AtPYL7 (AT4G01026) |
| AtPYL7 (AT4G01026) | OsPYL12 (Os02g15620) |
| AtPYL10 (AT4G27920) | AtPYL8 (AT5G53160) |
| AtPYL8 (AT5G53160) | OsPYL12 (Os02g15620) |
| AtPYL13 (AT4G18620) | AtPYL11 (AT5G45860) |
| OsPYL3 (Os02g13330) | OsPYL2 (Os06g36670) |
| OsPYL4 (Os01g61210) | OsPYL5 (Os05g39580) |
| OsPYL10 (Os02g15640) | OsPYL7 (Os06g33480) |
