# Supplementary figures and images for: Genome-wide identification and characterization of ABA receptor PYL gene family in rice
Source: BMC Genomics. 2020 Sep 30;21:676. doi: 10.1186/s12864-020-07083-y (PMC7526420; doi:10.1186/s12864-020-07083-y)

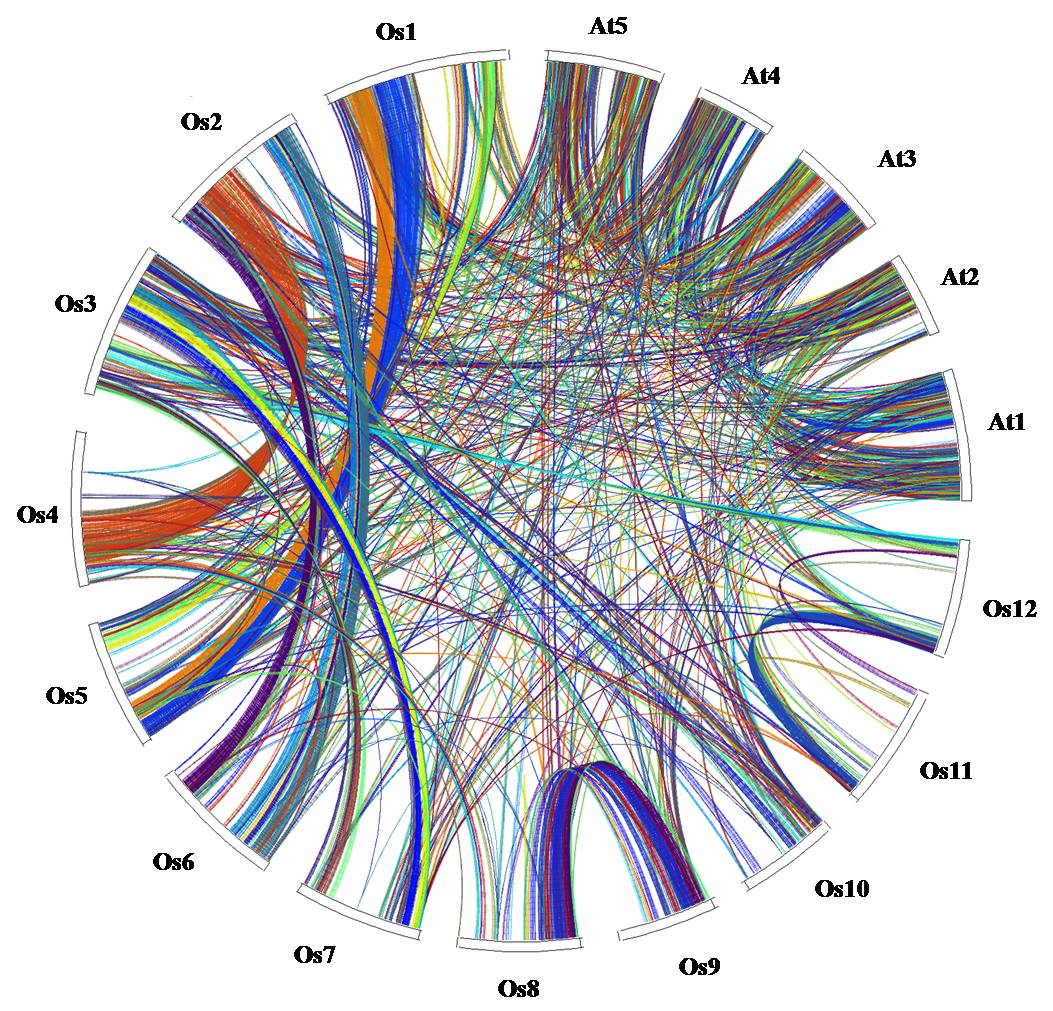

Supplement: Supplementary file 6 — Additional file 6 : Figure S1.Synteny blocks between Arabidopsis and rice at genomic level. [file 12864_2020_7083_MOESM6_ESM.jpg]

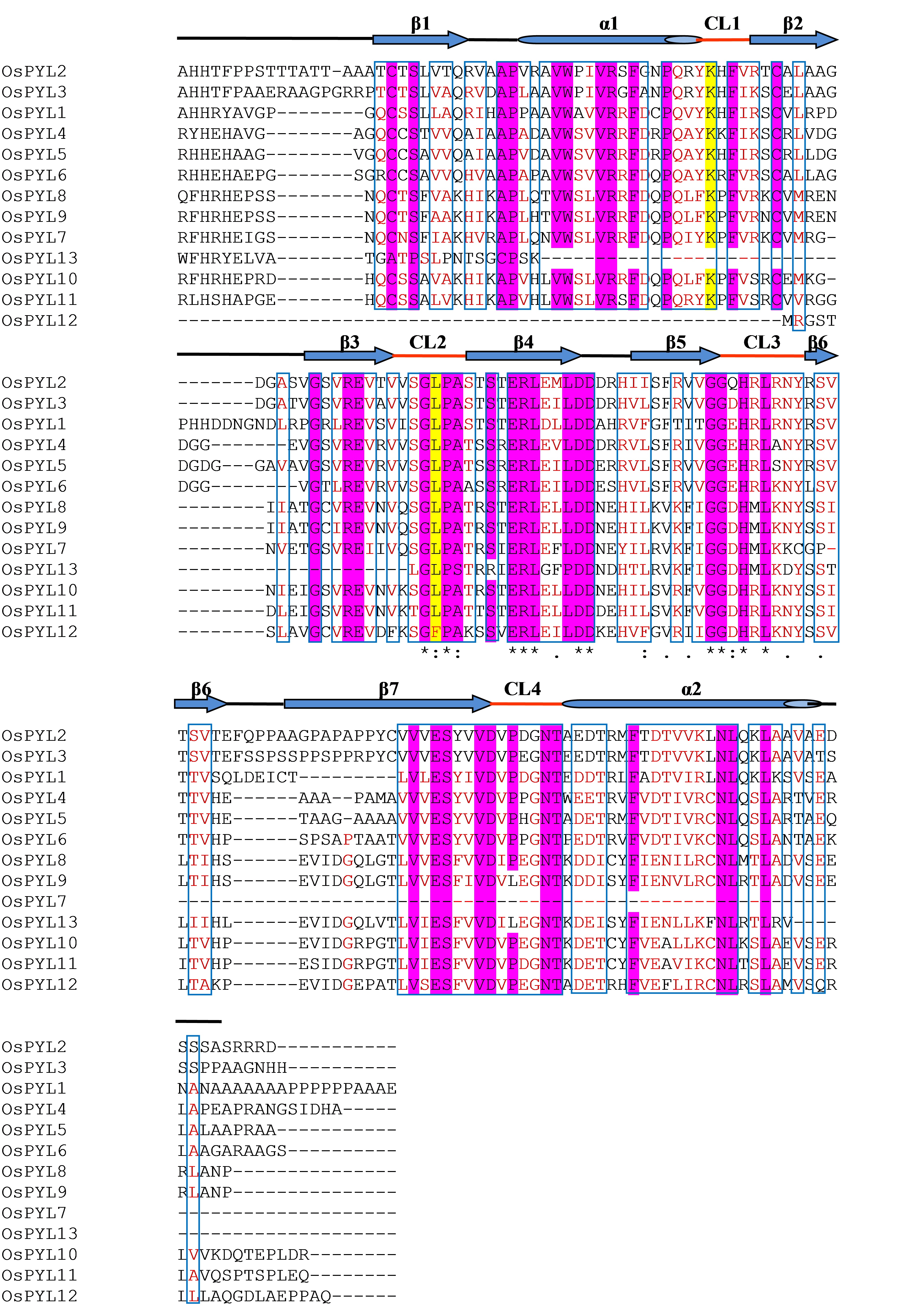

Supplement: Supplementary file 9 — Additional file 9 : Figure S2. Sequence alignment of 13 OsPYL proteins depicting four conserved loops CL1–CL4. [file 12864_2020_7083_MOESM9_ESM.jpg]

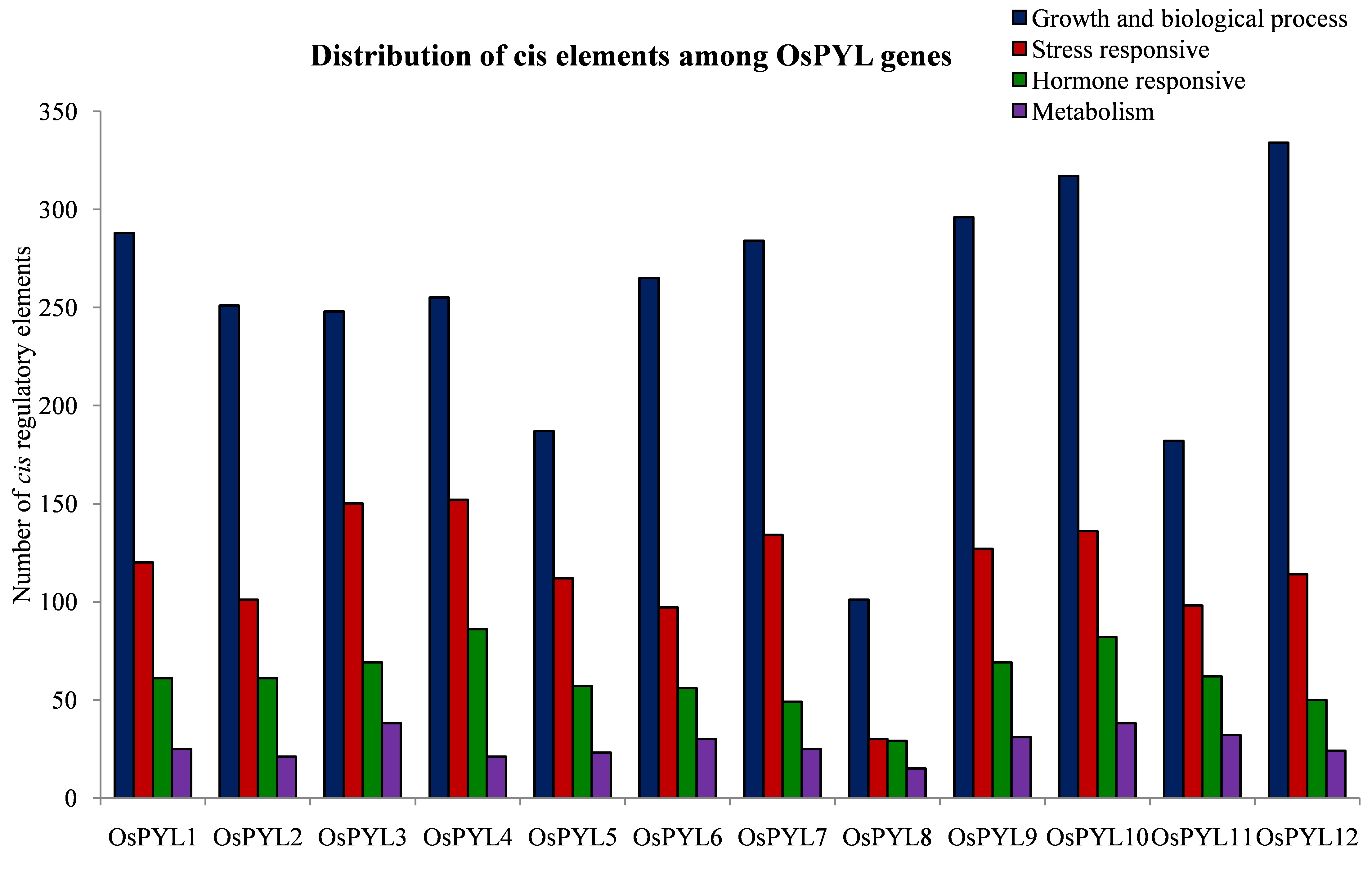

Supplement: Supplementary file 11 — Additional file 11 : Figure S3. Frequency and distribution of identified CRE in individual OsPYL promoter. [file 12864_2020_7083_MOESM11_ESM.jpg]
