# Supplementary material for: Genome-wide identification and characterization of ABA receptor PYL gene family in rice
Source: BMC Genomics. 2020 Sep 30;21:676. doi: 10.1186/s12864-020-07083-y (PMC7526420; doi:10.1186/s12864-020-07083-y)
Supplement: Supplementary file 12 — Additional file 12 : Table S3. List of homologous gene pair between Arabidopsis and rice. [file 12864_2020_7083_MOESM12_ESM.docx]

**Additional File 12 Table S3.** Homologous gene pairs identified from collinearity analysis among PYLs from *A. thaliana*, *B. distachyon*, *O. sativa*, *Z. mays*, *S. bicolor* and *H. vulgare* at genome wide scale

| **Source** | **Target** |
| --- | --- |
| AT4G17870 | AT5G46790 |
| AT5G46790 | Os10g42280 |
| AT2G38310 | AT5G05440 |
| AT2G38310 | BdiBd21-3.2G0295300 |
| AT2G38310 | HORVU1Hr1G070420 |
| AT2G38310 | Os01g61210 |
| AT2G38310 | Os05g39580 |
| AT2G38310 | Sobic.003G342000 |
| AT2G38310 | Sobic.009G170700 |
| AT2G40330 | AT5G05440 |
| AT5G05440 | BdiBd21-3.1G0877900 |
| AT5G05440 | BdiBd21-3.2G0688900 |
| AT5G05440 | BdiBd21-3.2G0295300 |
| AT5G05440 | HORVU1Hr1G070420 |
| AT5G05440 | Os01g61210 |
| AT5G05440 | Os03g18600 |
| AT5G05440 | Os05g39580 |
| AT5G05440 | Sobic.003G342000 |
| AT5G05440 | Sobic.009G170700 |
| AT5G05440 | GRMZM2G169695 |
| AT5G05440 | GRMZM2G057959 |
| AT2G40330 | BdiBd21-3.1G0220600 |
| AT2G40330 | BdiBd21-3.1G0877900 |
| AT2G40330 | BdiBd21-3.2G0688900 |
| AT2G40330 | BdiBd21-3.2G0295300 |
| AT2G40330 | HORVU1Hr1G070420 |
| AT2G40330 | HORVU3Hr1G088100 |
| AT2G40330 | HORVU4Hr1G055220 |
| AT2G40330 | Os01g61210 |
| AT2G40330 | Os03g18600 |
| AT2G40330 | Os05g39580 |
| AT2G40330 | Sobic.001G403300 |
| AT2G40330 | Sobic.003G342000 |
| AT2G40330 | Sobic.009G170700 |
| AT2G40330 | GRMZM2G141382 |
| AT1G01360 | AT4G01026 |
| AT4G01026 | BdiBd21-3.3G0129900 |
| AT4G01026 | Os02g15620 |
| AT4G01026 | Sobic.004G113800 |
| AT4G27920 | AT5G53160 |
| AT5G53160 | BdiBd21-3.3G0129900 |
| AT5G53160 | Os02g15620 |
| AT5G53160 | Sobic.004G113800 |
| AT4G18620 | AT5G45860 |
| BdiBd21-3.3G0446400 | HORVU1Hr1G050110 |
| BdiBd21-3.3G0446400 | Os10g42280 |
| BdiBd21-3.3G0446400 | Sobic.001G289100 |
| BdiBd21-3.3G0446400 | GRMZM2G134731 |
| BdiBd21-3.1G0512800 | BdiBd21-3.3G0115400 |
| BdiBd21-3.3G0115400 | HORVU7Hr1G088140 |
| BdiBd21-3.3G0115400 | Os02g13330 |
| BdiBd21-3.3G0115400 | Os06g36670 |
| BdiBd21-3.3G0115400 | Sobic.010G169100 |
| BdiBd21-3.3G0115400 | Sobic.004G097800 |
| BdiBd21-3.3G0115400 | GRMZM2G154987 |
| BdiBd21-3.1G0512800 | HORVU7Hr1G088140 |
| BdiBd21-3.1G0512800 | Os02g13330 |
| BdiBd21-3.1G0512800 | Os06g36670 |
| BdiBd21-3.1G0512800 | Sobic.010G169100 |
| BdiBd21-3.1G0512800 | Sobic.004G097800 |
| BdiBd21-3.1G0220600 | BdiBd21-3.1G0877900 |
| BdiBd21-3.1G0220600 | HORVU2Hr1G014670 |
| BdiBd21-3.1G0220600 | HORVU4Hr1G055220 |
| BdiBd21-3.1G0220600 | Os03g18600 |
| BdiBd21-3.1G0220600 | Sobic.001G403300 |
| BdiBd21-3.1G0220600 | GRMZM2G144224 |
| BdiBd21-3.1G0220600 | GRMZM2G057959 |
| BdiBd21-3.1G0877900 | HORVU4Hr1G055220 |
| BdiBd21-3.1G0877900 | Os03g18600 |
| BdiBd21-3.1G0877900 | Sobic.001G403300 |
| BdiBd21-3.1G0877900 | GRMZM2G144224 |
| BdiBd21-3.1G0877900 | GRMZM2G057959 |
| BdiBd21-3.2G0295300 | BdiBd21-3.2G0688900 |
| BdiBd21-3.2G0295300 | HORVU1Hr1G070420 |
| BdiBd21-3.2G0295300 | HORVU3Hr1G088100 |
| BdiBd21-3.2G0295300 | Os01g61210 |
| BdiBd21-3.2G0295300 | Os05g39580 |
| BdiBd21-3.2G0295300 | Sobic.003G342000 |
| BdiBd21-3.2G0295300 | Sobic.009G170700 |
| BdiBd21-3.2G0295300 | GRMZM2G141382 |
| BdiBd21-3.2G0295300 | GRMZM2G169695 |
| BdiBd21-3.2G0688900 | HORVU1Hr1G070420 |
| BdiBd21-3.2G0688900 | HORVU3Hr1G088100 |
| BdiBd21-3.2G0688900 | Os01g61210 |
| BdiBd21-3.2G0688900 | Os05g39580 |
| BdiBd21-3.2G0688900 | Sobic.001G403300 |
| BdiBd21-3.2G0688900 | Sobic.003G342000 |
| BdiBd21-3.2G0688900 | Sobic.009G170700 |
| BdiBd21-3.2G0688900 | GRMZM2G141382 |
| BdiBd21-3.2G0688900 | GRMZM2G169695 |
| BdiBd21-3.3G0129900 | HORVU7Hr1G079270 |
| BdiBd21-3.3G0129900 | Os02g15620 |
| BdiBd21-3.3G0129900 | Os06g33480 |
| BdiBd21-3.3G0129900 | Sobic.004G113800 |
| BdiBd21-3.3G0129900 | GRMZM2G048733 |
| BdiBd21-3.3G0129900 | GRMZM2G063882 |
| AT4G18620 | BdiBd21-3.2G0404900 |
| BdiBd21-3.2G0404900 | HORVU1Hr1G030390 |
| BdiBd21-3.2G0404900 | Os05g12260 |
| BdiBd21-3.2G0404900 | Sobic.009G080200 |
| BdiBd21-3.2G0404900 | GRMZM2G133631 |
| BdiBd21-3.2G0404900 | GRMZM2G165567 |
| HORVU1Hr1G050110 | Os10g42280 |
| HORVU1Hr1G050110 | Sobic.001G289100 |
| HORVU1Hr1G050110 | GRMZM2G134731 |
| HORVU1Hr1G070420 | HORVU3Hr1G088100 |
| HORVU3Hr1G088100 | Os01g61210 |
| HORVU3Hr1G088100 | Os05g39580 |
| HORVU3Hr1G088100 | Sobic.003G342000 |
| HORVU3Hr1G088100 | Sobic.009G170700 |
| HORVU3Hr1G088100 | GRMZM2G141382 |
| HORVU3Hr1G088100 | GRMZM2G169695 |
| HORVU7Hr1G088140 | Os02g13330 |
| HORVU7Hr1G088140 | Os06g36670 |
| HORVU7Hr1G088140 | Sobic.010G169100 |
| HORVU7Hr1G088140 | Sobic.004G097800 |
| HORVU7Hr1G088140 | GRMZM2G154987 |
| HORVU1Hr1G070420 | Os01g61210 |
| HORVU1Hr1G070420 | Os05g39580 |
| HORVU1Hr1G070420 | Sobic.003G342000 |
| HORVU1Hr1G070420 | Sobic.009G170700 |
| HORVU1Hr1G070420 | GRMZM2G141382 |
| HORVU1Hr1G070420 | GRMZM2G169695 |
| HORVU4Hr1G055220 | Os03g18600 |
| HORVU4Hr1G055220 | Sobic.001G403300 |
| HORVU4Hr1G055220 | GRMZM2G144224 |
| HORVU4Hr1G055220 | GRMZM2G057959 |
| HORVU7Hr1G079270 | Os02g15620 |
| HORVU7Hr1G079270 | Os06g33480 |
| HORVU7Hr1G079270 | Sobic.004G113800 |
| HORVU7Hr1G079270 | GRMZM2G048733 |
| HORVU7Hr1G079270 | GRMZM2G063882 |
| Os10g42280 | Sobic.001G289100 |
| Os10g42280 | GRMZM2G134731 |
| Os02g13330 | Os06g36670 |
| Os06g36670 | Sobic.010G169100 |
| Os06g36670 | Sobic.004G097800 |
| Os06g36670 | GRMZM2G154987 |
| Os02g13330 | Sobic.010G169100 |
| Os02g13330 | Sobic.004G097800 |
| Os02g13330 | GRMZM2G154987 |
| Os01g61210 | Os05g39580 |
| Os01g61210 | Sobic.003G342000 |
| Os01g61210 | Sobic.009G170700 |
| Os01g61210 | GRMZM2G141382 |
| Os01g61210 | GRMZM2G169695 |
| Os05g39580 | Sobic.003G342000 |
| Os05g39580 | Sobic.009G170700 |
| Os05g39580 | GRMZM2G141382 |
| Os05g39580 | GRMZM2G169695 |
| Os03g18600 | Sobic.001G403300 |
| Os03g18600 | GRMZM2G144224 |
| Os03g18600 | GRMZM2G057959 |
| Os02g15640 | Os06g33480 |
| Os06g33480 | Sobic.004G113800 |
| Os06g33480 | GRMZM2G048733 |
| Os06g33480 | GRMZM2G063882 |
| Os05g12260 | Sobic.009G080200 |
| Os05g12260 | GRMZM2G165567 |
| Os02g15620 | Sobic.004G113800 |
| Os02g15620 | GRMZM2G048733 |
| Os02g15620 | GRMZM2G063882 |
| Sobic.010G169100 | Sobic.004G097800 |
| Sobic.004G097800 | GRMZM2G154987 |
| Sobic.010G169100 | GRMZM2G154987 |
| Sobic.001G403300 | Sobic.003G342000 |
| Sobic.001G403300 | GRMZM2G144224 |
| Sobic.001G403300 | GRMZM2G057959 |
| Sobic.003G342000 | Sobic.009G170700 |
| Sobic.009G170700 | GRMZM2G141382 |
| Sobic.009G170700 | GRMZM2G169695 |
| Sobic.003G342000 | GRMZM2G141382 |
| Sobic.003G342000 | GRMZM2G169695 |
| Sobic.009G080200 | GRMZM2G133631 |
| Sobic.009G080200 | GRMZM2G165567 |
| Sobic.004G113800 | GRMZM2G048733 |
| Sobic.004G113800 | GRMZM2G063882 |
| GRMZM2G144224 | GRMZM2G057959 |
| GRMZM2G141382 | GRMZM2G169695 |
| GRMZM2G048733 | GRMZM2G063882 |
| GRMZM2G141382 | GRMZM2G169695 |
