# Supplementary material for: Genome-wide identification and characterization of ABA receptor PYL gene family in rice
Source: BMC Genomics. 2020 Sep 30;21:676. doi: 10.1186/s12864-020-07083-y (PMC7526420; doi:10.1186/s12864-020-07083-y)
Supplement: Supplementary file 13 — Additional file 13 : Table S4. List of primers used for q-RT expression analysis of 13 OsPYL genes. [file 12864_2020_7083_MOESM13_ESM.docx]

**Additional File 13 Table S1:** Primers used for quantitative RT-PCR

| **Gene** | **Sequence (5’-3’)** | |
| --- | --- | --- |
|  | **Forward** | **Reverse** |
| OsPYL1 | GAGTCCTACATCGTCGACGTC | GAAGTGAAATTGCAGAGAGGTC |
| OsPYL2 | CACCTCCGTCACCGAGTTCCA | ATTGACGAGGCCTCTCCTAGTCG |
| OsPYL3 | CTCGAGATCCTCGACGACGAC | CACCATGATCAATCACTAATGGTG |
| OsPYL4 | CTCGGTGACCACCGTCCACGA | TGCATCATGCATGATCGATCGATC |
| OsPYL5 | GAGCACCGCCTCTCCAACTAC | GAGAGGGAGAGCTAGCTATGATC |
| OsPYL6 | GAGAGCCACGTCCTCAGCTTC | CCGAAGAAACACACATCCATC |
| OsPYL7 | GAGATGAACAGTGGCGCTGGT | CTAACGTGCTTCGCAATGAAG |
| OsPYL8 | CATCCATCCTAACCATCCACTC | ACCAAGAAAGCAGAGTGAGATC |
| OsPYL9 | GAAGAATTACTCATCCATCCTGAC | AATCAACTAAGAAAGCAGGGTG |
| OsPYL10 | CAAGGATGAGACATGCTACTTCGT | GAACCTAAGGGCTCCATTGGAG |
| OsPYL11 | CTCATCCATCGTAACTGTCCATC | GTGATGACCCTACTGTTCAAGTG |
| OsPYL12 | CAAGAATTACTCGTCAGTGTTGAC | GTCTAAAATCCAGTCATTGTGCAG |
| OsPYL13 | ACCGCTATGAGCTCGTTGCAA | ATCAAGGTGGATGAGTAGTC |
| OsUBQ5 | GAAGCACAAGCACAAGAAGGTG | CTGGTTGTAGACGTAGGTGAG |
